# A nomogram to predict mortality in patients with severe fever with thrombocytopenia syndrome

**DOI:** 10.1038/s41598-024-60923-9

**Published:** 2024-05-09

**Authors:** Kun Fang, Xuezhen Song, Jinshuang Bo

**Affiliations:** Department of Transfusion Medicine, Weihai Municipal Hospital, No. 70 of Heping Road, WeihaiShandong, 264200 China

**Keywords:** Diseases, Medical research, Pathogenesis, Risk factors, Signs and symptoms

## Abstract

Severe fever with thrombocytopenia syndrome (SFTS) is an acute infectious disease caused by a novel Bunyavirus infection with low population immunity and high mortality rate. Lacking specific therapies, the treatment measures vary with the severity of the disease, therefore, a case control study involved 394 SFTS patients was taken to determine risk factors for mortality. Comparative clinical data from the first 24 h after admission was collected through the electronic medical record system. Independent risk factors for death of SFTS were identified through univariate and multivariate binary logistic regression analyses. The results of the logistic regression were visualized using a nomogram which was created by downloading RMS package in the R program. In our study, four independent mortality risk factors were identified: advanced age(mean 70.45 ± 7.76 years), MODS, elevated APTT, and D-dimer. The AUC of the nomogram was 0.873 (0.832, 0.915), and the model passes the calibration test namely Unreliability test with P = 0.958, showing that the model's predictive ability is excellent. The nomogram to determine the risk of death in SFTS efficiently provide a basis for clinical decision-making for treatment.

## Introduction

Severe Fever with Thrombocytopenia Syndrome (SFTS) is an acute infectious disease caused by a tick-borne Bunyavirus infection, characterized by fever with thrombocytopenia, accompanied by fatigue, obvious gastrointestinal symptoms (poor appetite, nausea, vomiting, and diarrhea), headache, myalgia and additional manifestations. A subset of patients may experience severe and swiftly advancing illness, leading to potential fatalities from multiple organ dysfunction syndrome. Initially identified in 2009^1^, SFTS has seen a rising number of cases in China, with successive reports of infections in Japan and Korea^2,3^. People began to realize that it was a tick-mediated virus disease after Xuejie Yu et al. reported the isolation of the virus from ticks^1,4^. Related to the tick habitat, the disease is at a higher risk of infection in residents and workers living in hilly, mountainous and forested areas, as well as in tourists visiting such areas for outdoor activities. As research deepened, it was found that pets, livestock and wild animals can also harbor this virus^5^ and human-to-human transmission is possible^6,7^. Due to its low population immunity and high mortality rate, the Chinese government had attached great importance to this disease, in collaboration with experts, formulated the Severe fever with Thrombocytopenia Syndrome Prevention and Control Guidelines (2010 version)^8^ to regulate the diagnosis and treatment of SFTS. SFTSV was listed as a kind of priority pathogen by WHO in 2018 for its potential harm to public health without sufficient countermeasures^9^.

At present, the challenge is that there are no conclusive randomized controlled trials proving the effectiveness of antiviral treatment measures^10,11^, although ribavirin and favipiravir are both clinically employed. Consequently, SFTS management primarily involves symptomatic supportive therapy. For example, physical cooling for high fever, pharmacological antipyretics if necessary, plasma and platelet transfusions for significant bleeding or low platelet count (less than 30 × 10^9^/L), and granulocyte colony-stimulating factor for severe neutrophil depression (less than 1 × 10^9^/L). When there are secondary bacterial and fungal infections, sensitive antibiotics and antifungals should be selected^12^. In addition, there are steroids, plasma exchange, and intravenous immunoglobulins (IVIg), etc., but they are still controversial^13–15^.

The treatment measures taken varies with the severity of the disease, and clinical outcomes also vary. Therefore, it is important to determine the severity of the disease, i.e., the risk of death. We designed this case–control study to compare the clinical information between the death and survival groups using confirmed SFTS cases in our hospital in order to screen independent risk factors by univariate and multivariate binary logistic regression. Then we utilized these factors to plot a nomogram, an emerging method assisting the receiving physicians to calculate the predicted mortality of SFTS patients and adopt different interventions for different risk groups, aiming to improve clinical outcomes and reduce mortality. Additionally, it offers a population stratification method for the next intervention studies.

## Methods

### Patients

We retrospectively studied 467 patients infected by severe fever with thrombocytopenia syndrome virus (SFTSV), given that viral RNA was detected in serum via reverse transcriptase polymerase chain reaction (RT-PCR) in Weihai Municipal Hospital which is a tertiary hospital in Shandong Province, China from 2012–2021, of which 394 patients presented with a temperature greater than 37.3 °C and platelet count below 100 × 10^9^/L were included in the study. This study was approved by the Ethics Committee of Weihai Municipal Hospital and complied with the principles of the Declaration of Helsinki. Informed consent was waived because of the retrospective nature of our study.

### Methods

The study subjects were categorized into two groups: those who survived and those who succumbed to SFTS. The latter group includes patients who died during their SFTS treatment. We collected clinical data completed within 24 h of admission, encompassing epidemiological and demographic details, clinical symptoms, physical examination findings, and laboratory test results. This data was gathered via the hospital's electronic medical record system by trained staff. Subsequently, we analyzed patients' general characteristics, underlying health conditions, symptoms, signs, and complications, alongside viral load, blood routine, biochemistry, electrolytes and coagulation function tests (Supplementary Information).

### Statistical methods

All data were analyzed by SPSS 25.0 (IBM, Armonk, NY, USA). Continuous variables are expressed as mean ± standard deviation if they conform to normality, otherwise they are expressed as median with interquartile ranges. Categorical variables are represented by frequencies with percentages. Independent *t*-test or Mann–Whitney *U* test was taken for comparisons between two groups of continuous variables, but when it came to two rates, the chi-square test or Fisher’s exact test was selected. Independent risk factors for death of SFTS were derived by univariate and multivariate binary logistic regression.

The results of the binary logistic regression were visualized using a nomogram, created by downloading the RMS package in the R version 4.2.1 (The R Project for Statistical Computing, http://www.r-project.org) and employing the ‘nomagram’ function. Discrimination and calibration were used to evaluate the model, with discrimination degree measured by the area under receiver operating characteristics (ROC) curve. It is generally considered to be < 0.60 for poor distinction, 0.60–0.75 for distinction with some value, and > 0.75 for superior distinction. Boot resampling was used to obtains the area under curve (AUC) of internal validation, concurrently, the resampling ROC curve with confidence interval was drawn. Furthermore, a calibration curve representing the calibration degree was drawn. The closer the calibration curve aligns with the standard curve, the better the calibration of the prediction model is. The maximum value of Youden Index, defined as specificity plus sensitivity minus one, was employed as the cut-off value of risk classification for death. Bilateral P values below 0.05 were set up as a statistical significance level in all statistical tests.

## Results

### General characteristics

**Figure 1:**
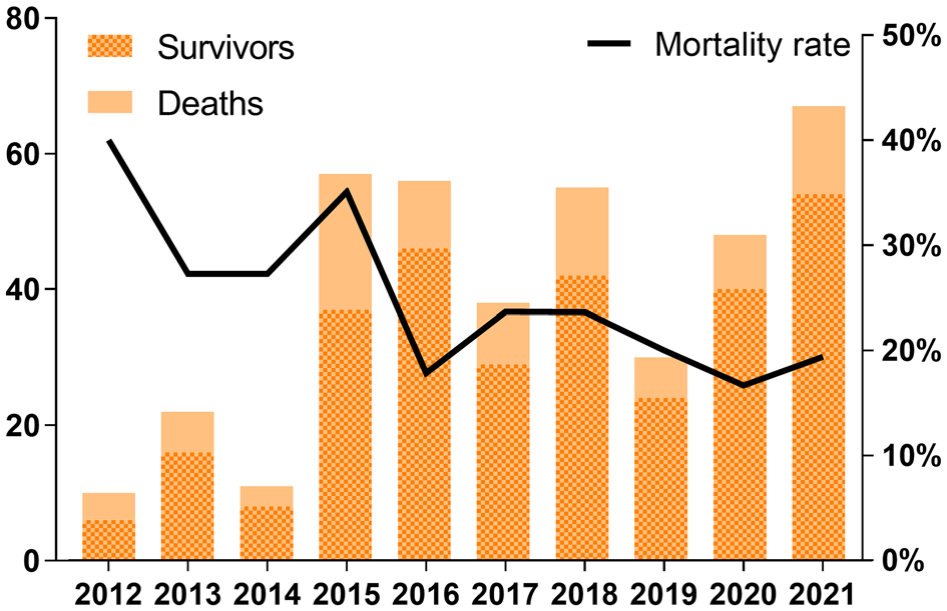
The number of deaths vs. Survivors and the mortality rate of the 394 confirmed severe fever with thrombocytopenia syndrome (SFTS) from 2012 to 2021.

**Figure 2:**
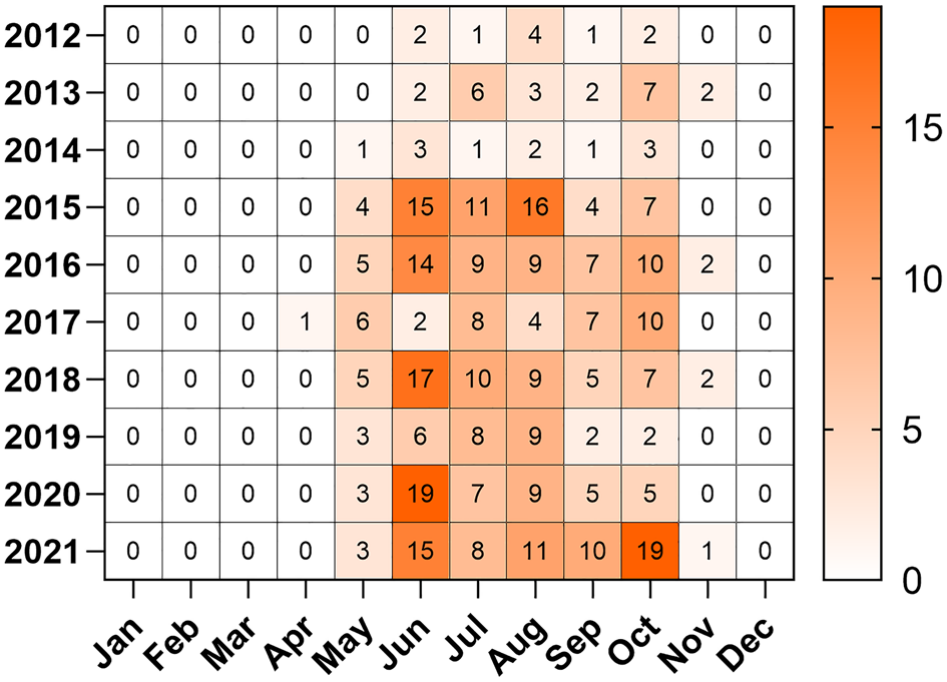
Heat map of severe fever with thrombocytopenia syndrome (SFTS) onset by month from 2012 to 2021.

A total of 394 patients diagnosed with SFTS from 2012 to 2021 were from Weihai City and its subordinate counties, Shandong Province, with a mortality rate of 23.35%. Notably, while the annual number of cases exhibited an increasing trend, the mortality rate decreased after peaking in 2012 and 2015, stabilizing at approximately 20% (Fig. 1). The incidence of SFTS occurred from April to November, mainly prevalent in May to October (Fig. 2).

The average age of the 394 patients was 65.68 ± 10.14 years. In the survival group, the average age was 64.22 ± 10.34 years, compared to 70.45 ± 7.76 years in the mortality group. This age difference was statistically significant (P < 0.001) (Table 1). However, no statistical difference was found in gender distribution. Farmers accounted for 33.2% of the study population, 13.2% had a history of tick bites, and a minority of patients had contact with animals one week before the onset of the disease. Nevertheless, none of these factors showed a statistically significant difference when comparison between the two groups was performed.

**Table 1:** General characteristics compared between the survival and death group. Significant values are in bold.

Variables	All (n = 394)	Survival (n = 302)	Death (n = 92)	P
General characteristics, n (%)				
Age (year)	65.68 ± 10.14	64.22 ± 10.34	70.45 ± 7.76	**< 0.001**
Gender				
Male	189 (48.0)	139 (46.0)	50 (54.3)	0.162
Female	205 (52.0)	163 (54.0)	42 (45.7)	
Farmer	131 (33.2)	104 (34.4)	27 (29.3)	0.364
Tick bite	52 (13.2)	37 (12.3)	15 (16.3)	0.315
Field activities	24 (6.1)	19 (6.3)	5 (5.4)	0.764
History of animal contact	11 (2.8)	10 (3.3)	1 (1.1)	0.440
Onset of illness to admission (day)	5 (4.7)	5 (4.7)	5 (4.7)	0.848

### Clinical features

The median body temperature was 38.7 (38.4, 39.0) °C in both groups and was not statistically different. The same was true for other general symptoms. Once the central nervous system was affected, the result was often fatal. When examining central nervous system symptoms, we found no statistically significant difference in the occurrence of headache (13.9% vs. 7.6%) and dizziness (22.8% vs. 18.5%) between the survival and death groups. However, consciousness disturbance (20.9% vs. 40.2%, P < 0.001) and seizure (1.0% vs. 5.4%, P = 0.026) had significant differences (Table 2). In addition, myalgia was observed in 31.5% of patients in the survival group, which was significantly greater than 18.5% in the death group (Table 2). Muscle tremor, on the other hand, did not exhibit a statistically significant difference between the two groups.

**Table 2:** Clinical characteristics compared between the survival and death group.

Variables	All (n = 394)	Survival (n = 302)	Death (n = 92)	P
Underlying diseases, n (%)				
Hypertension	111 (28.2)	84 (27.8)	27 (29.3)	0.775
Diabetes	68 (17.3)	55 (18.2)	13 (14.1)	0.364
Cardiovascular diseases	33 (8.4)	24 (7.9)	9 (9.8)	0.578
Respiratory system diseases	31 (7.9)	9 (9.8)	22 (7.3)	0.436
Digestive system diseases	48 (12.2)	35 (11.6)	13 (14.1)	0.514
Urological diseases	6 (1.5)	5 (1.7)	1 (1.1)	1.000
Symptoms, n (%)				
Fatigue	287 (72.8)	225 (74.5)	62 (67.4)	0.179
Chill	200 (50.8)	160 (53.0)	40 (43.5)	0.110
Anorexia	190 (48.2)	142 (47.0)	48 (52.2)	0.386
Nausea	160 (40.6)	129 (42.7)	31 (33.7)	0.123
Vomiting	116 (29.4)	89 (29.5)	27 (29.3)	0.982
Abdominal pain	49 (12.4)	36 (11.9)	13 (14.1)	0.574
Diarrhea	163 (41.4)	122 (40.4)	41 (44.6)	0.477
Cough/expectoration	55 (14.0)	39 (12.9)	16 (17.4)	0.278
Headache	49 (12.4)	42 (13.9)	7 (7.6)	0.109
Dizzy	86 (21.8)	69 (22.8)	17 (18.5)	0.374
Consciousness disturbance	100 (25.4)	63 (20.9)	37 (40.2)	**< 0.001**
Seizures/cramp	8 (2.0)	3 (1.0)	5 (5.4)	**0.026**
Myalgia	112 (28.4)	95 (31.5)	17 (18.5)	**0.016**
Muscle tremor	100 (25.4)	73 (24.2)	27 (29.3)	0.318
Signs, n (%)				
Temperature (°C)	38.7 (38.4, 39.0)	38.7 (38.4, 39.0)	38.7 (38.4, 39.0)	0.881
Acute ill-looking	18 (4.6)	11 (3.6)	7 (7.6)	0.190
Gingival bleeding/ecchymosis	26 (6.6)	16 (5.3)	10 (10.9)	0.059
Lymphadenopathy	187 (47.5)	147 (48.7)	40 (43.5)	0.382
Abdominal tenderness	58 (14.7)	46 (15.2)	12 (13.0)	0.604
Percussion pain in liver and kidney region	27 (6.9)	22 (7.3)	5 (5.4)	0.539
Lung rales	47 (11.9)	37 (12.3)	10 (10.9)	0.720
Complications, n (%)				
Bloodstream infection	18 (4.6)	13 (4.3)	5 (5.4)	0.866
SIRS	15 (3.8)	9 (3.0)	6 (6.5)	0.214
Septic shock	46 (11.7)	31 (10.3)	15 (16.3)	0.114
Pneumonia	132 (33.5)	105 (34.8)	27 (29.3)	0.335
Respiratory failure	37 (9.4)	29 (9.6)	8 (8.7)	0.794
Electrolyte disturbance	61 (15.5)	47 (15.6)	14 (15.2)	0.936
Hypoproteinemia	47 (11.9)	40 (13.2)	7 (7.6)	0.144
Acute liver function injury	40 (10.2)	36 (11.9)	4 (4.3)	**0.035**
Acute renal injury	18 (4.6)	15 (5.0)	3 (3.3)	0.688
Acute nervous system injury	4 (1.0)	2 (0.7)	2 (2.2)	0.234
Gastrointestinal bleeding	30 (7.6)	21 (7.0)	9 (9.8)	0.370
Coagulation disorders/thrombosis	18 (4.6)	14 (4.6)	4 (4.3)	1.000
Heart failure/arrhythmia	65 (16.5)	47 (15.6)	18 (19.6)	0.365
MODS	132 (33.5)	78 (25.8)	54 (58.7)	**< 0.001**

*SIRS* systemic inflammatory response syndrome, *MODS* multiple organ dysfunction syndrome.

Significant values are in bold.

After physical examination, lymphadenopathy (47.5%) was the most common sign of SFTS. Notably, the proportion of patients displaying acute ill-looking (7.6% vs. 3.6%) and experiencing gingival bleeding or ecchymosis (10.9% vs. 5.3%) was higher in the deceased group than in the surviving group respectively. Conversely, the proportion of patients with percussion pain in the liver and kidney area (7.3% vs. 5.4%) was slightly higher in the surviving group than in the deceased group, respectively, although these were not statistically significant differences.

The most prevalent complications were multiple organ dysfunction syndrome (MODS) (33.5%) and pneumonia (33.5%). Obviously, there was a significantly higher incidence of MODS in the case group compared to the control group (58.7% vs. 25.8%, P < 0.001) (Table 2). However, there was no statistical difference in the percentage of pneumonia when comparing the two groups. Additionally, several other complications did not exhibit statistically significant differences.

### Laboratory tests

Viral load was significantly different between the survival and death groups (P < 0.001). The survival group predominantly had a viral load of < 103, whereas the death group had mostly viral loads above 103, indicating a statistically significant distinction (Table 3). Leukocyte count and lymphocyte count, neutrophil count, erythrocyte count, and hemoglobin were not statistically significant differences. However, platelet count was significantly lower in the death group than in the survival group (P < 0.001) (Table 3).

**Table 3:** Laboratory parameters compared between the survival and death group.

Variables	All (n = 394)	Survival (n = 302)	Death (n = 92)	P
Laboratory parameters				
Viral load (TCID 50/mL)*				**< 0.001**
< 10^3^	232 (58.9)	208 (68.9)	24 (26.1)	
10^3^–10^6^	134 (34.0)	90 (29.8)	44 (47.8)	
> 10^6^	28 (7.1)	4 (1.3)	24 (26.1)	
Leukocytes (10^9^/L)	2.61 (1.69, 4.70)	2.67 (1.62, 4.66)	2.50 (1.75, 5.00)	0.847
Neutrophils (10^9^/L)	1.63 (1.03, 3.38)	1.60 (1.03, 3.73)	1.67 (1.04, 2.68)	0.944
Lymphocyte (10^9^/L)	0.61 (0.39, 0.96)	0.62 (0.40, 0.96)	0.55 (0.34, 0.97)	0.142
Erythrocyte (10^12^/L)	4.44 ± 0.57	4.43 ± 0.53	4.46 ± 0.71	0.737
Hemoglobin (g/L)	134.39 ± 18.27	134.36 ± 17.08	134.47 ± 21.81	0.967
Platelets (10^9^/L)	51 (34, 66)	53 (36, 68)	40 (30, 57)	**< 0.001**
ALT (U/L)	66 (40, 124)	62 (38, 110)	95 (51, 164)	**< 0.001**
AST (U/L)	149.0 (73.3, 296.6)	125.4 (65.0, 245.6)	294.2 (137.0, 488.4)	**< 0.001**
CK (U/L)	543 (230, 1292)	428 (209, 912)	1017 (450, 2259)	**< 0.001**
CK-MB (U/L)	33.9 (19.9, 57.1)	28.2 (18.4, 46.4)	61.2 (31.2, 109.8)	**< 0.001**
LDH (U/L)	631 (347, 955)	552 (322, 847)	904 (567, 1737)	**< 0.001**
α-HBDH (U/L)	426 (253, 692)	380 (240, 605)	645 (364, 1071)	**< 0.001**
K (mmol/L)	3.81 (3.42, 4.14)	3.77 (3.40, 4.06)	4.03 (3.68, 4.39)	**< 0.001**
Na (mmol/L)	134.4 (131.0, 138.0)	134.0 (131.0, 138.0)	135.2 (131.0, 138.6)	0.429
Cl (mmol/L)	99 (96, 103)	99 (96, 103)	100 (96, 104)	0.536
BUN (mmol/L)	5.0 (3.8, 7.3)	4.6 (3.4, 6.7)	6.1 (4.7, 10.6)	**< 0.001**
sCr (mmol/L)	70.0 (58.0, 88.4)	66.7 (55.0, 80.4)	87.5 (67.3, 114.9)	**< 0.001**
PT (s)	13.0 (12.2, 14.9)	12.8 (12.1, 14.5)	14.2 (12.7, 15.8)	**< 0.001**
APTT (s)	43.4 (37.6, 51.3)	41.5 (37.0, 48.1)	51.9 (44.3, 66.3)	**< 0.001**
Fibrinogen (g/L)	2.01 (1.72, 2.31)	2.07 (1.78, 2.34)	1.81 (1.48, 2.19)	**< 0.001**
TT (s)	19.4 (18.3, 20.7)	19.1 (18.1, 20.2)	20.6 (19.4, 23.2)	**< 0.001**
D-dimer (μg/L)	3.52 (1.74, 8.13)	2.90 (1.34, 5.73)	8.78 (3.67, 17.04)	**< 0.001**

*The differences were statistically significant between surviving patients and deceased patients in the viral load < 10^3^ group compared with the 10^3^–10^6^ group (p < 0.001), in the < 10^3^ group compared with the > 10^6^ group (p < 0.001), and in the 10^3^–10^6^ group compared with the > 10^6^ group (p < 0.001).

*ALT* alanine aminotransferase, *AST* aspartate aminotransferase, *CK* creatinine kinase, *CK-MB* creatinine kinase myocardial b fraction, *LDH* lactate dehydrogenase, *α-HBDH* α-Hydroxybutyrate dehydrogenase, *K* potassium, *Na* sodium, *Cl* chloride, *BUN* Blood urea nitrogen, *sCr* serum creatinine, *PT* prothrombin time, *APTT* activated partial thromboplastin time, *TT* thrombin time.

Significant values are in bold.

Serum biochemical parameters, including alanine aminotransferase (ALT), aspartate aminotransferase (AST), creatinine kinase (CK), creatinine kinase myocardial b fraction (CK-MB), lactate dehydrogenase (LDH), and α-Hydroxybutyrate dehydrogenase (α-HBDH) were statistically higher in the death group (P < 0.001) (Table 3).

In terms of electrolytes, blood potassium was significantly higher in the deaths compared to the survivors (P < 0.001) (Table 3), but the serum potassium level in mortality cases do not appear clinically higher.

Moreover, urea nitrogen and creatinine levels were also significantly higher in the mortality group than in the survival group (P < 0.001) (Table 3). Coagulation indexes, including prothrombin time (PT), activated partial thromboplastin time (APTT), thrombin time (TT), fibrinogen, and D-dimer were also statistically different between the two groups (P < 0.001) (Table 3).

### Risk factors for death

Univariate binary logistics regression revealed several factors associated with an increased risk of a fatal outcome in SFTS patients. These risk factors included advanced age, consciousness disturbance, seizures/cramp, myalgia, MODS, acute liver injury, high viral load, low platelet count, elevated levels of ALT, AST, CK, CK-MB, LDH, α-HBDH, K, urea nitrogen, creatinine, prolonged APTT, TT, reduced fibrinogen, and raised D-dimer. The independent risk factors came out to be advanced age, MODS, elevated APTT and D-dimer by multivariate analysis (Table 4).

**Table 4:** Univariate and multivariate analysis of factors associated with fatal outcome among patients with SFTS.

Variables	Univariate analysis					Multivariate analysis				
	B	P	OR	95% CI for OR		B	P	OR	95% CI for OR	
				Lower	Upper				Lower	Upper
Age	0.070	< 0.001	1.073	1.044	1.103	0.074	**< 0.001**	1.077	1.035	1.119
Consciousness disturbance	0.937	< 0.001	2.552	1.547	4.211	0.524	0.141	1.688	0.841	3.390
Seizures/cramp	1.745	0.018	5.728	1.342	24.447	1.305	0.221	3.689	0.457	29.787
Myalgia	− 0.705	0.017	0.494	0.277	0.882	− 0.306	0.429	0.736	0.345	1.573
MODS	1.406	< 0.001	4.081	2.504	6.651	1.159	**0.001**	3.186	1.653	6.141
Acute liver function injury	− 1.091	0.044	0.336	0.116	0.970	− 0.944	0.209	0.389	0.089	1.699
Viral load (TCID 50/mL)	1.736	< 0.001	5.672	3.688	8.724	0.000	0.677	1.000	1.000	1.000
Platelets (10^9^/L)	− 0.025	< 0.001	0.975	0.963	0.987	− 0.014	0.129	0.987	0.969	1.004
ALT (U/L)	0.004	< 0.001	1.004	1.002	1.006	0.000	0.799	1.000	0.996	1.003
AST (U/L)	0.003	< 0.001	1.003	1.002	1.004	0.000	0.901	1.000	0.998	1.002
CK (U/L)	0.000	0.000	1.000	1.000	1.001	0.000	0.747	1.000	1.000	1.000
CK-MB (U/L)	0.020	< 0.001	1.020	1.014	1.027	0.004	0.433	1.004	0.993	1.015
LDH (U/L)	0.001	< 0.001	1.001	1.001	1.002	0.001	0.054	1.001	1.000	1.003
α-HBDH (U/L)	0.002	< 0.001	1.002	1.001	1.002	− 0.001	0.372	0.999	0.997	1.001
BUN (mmol/L)	0.100	0.002	1.106	1.052	1.162	− 0.015	0.769	0.986	0.895	1.086
sCr (mmol/L)	0.008	0.002	1.008	1.003	1.013	0.000	0.936	1.000	0.994	1.007
PT (s)	− 0.005	0.304	0.995	0.987	1.004	− 0.005	0.466	0.995	0.980	1.009
APTT (s)	0.080	< 0.001	1.083	1.057	1.109	0.036	**0.017**	1.037	1.007	1.068
Fibrinogen (g/L)	− 1.532	< 0.001	0.216	0.120	0.389	0.015	0.967	1.015	0.496	2.078
TT (s)	0.424	< 0.001	1.528	1.342	1.739	0.067	0.427	1.069	0.907	1.261
D-dimer (μg/L)	0.044	< 0.001	1.045	1.022	1.069	0.027	**0.004**	1.027	1.008	1.046

*MODS* multiple organ dysfunction syndrome, *ALT* alanine aminotransferase, *AST* aspartate aminotransferase, *CK* creatinine kinase, *CK-MB* creatinine kinase myocardial b fraction, *LDH* lactate dehydrogenase, *ɑ-HBDH* ɑ-Hydroxybutyrate dehydrogenase, *BUN* Blood urea nitrogen, *sCr* serum creatinine, *PT* prothrombin time, *APTT* activated partial thromboplastin time, *TT* thrombin time. Significant values are in bold.

### Nomogram

**Figure 3:**
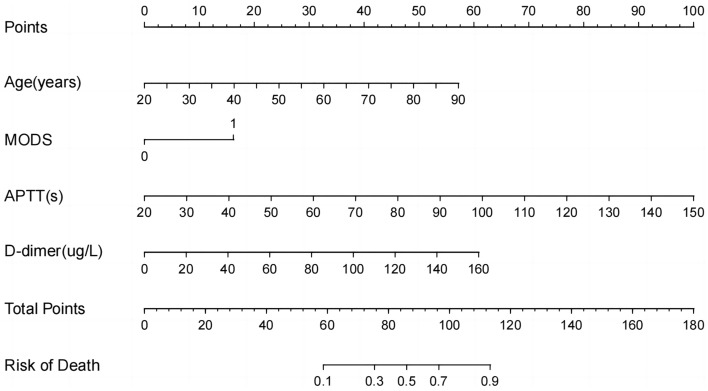
The nomogram for predicting mortality rate of patients with severe fever with thrombocytopenia syndrome (SFTS). The nomogram includes the individual scores, i.e. Point (first row), which can be derived by making a vertical line upward at each variable, and the total score, i.e. Total Point (last row), which represents the sum of the individual scores taken together, and the predicted death can be obtained by drawing a vertical line downward. *MODS* multiple organ dysfunction syndrome, *APTT* activated partial thromboplastin time.

The four independent risk factors derived from multivariate binary logistic regression analysis were plotted in a nomogram by integration (Fig. 3). They were drawn on the same plane at a certain scale, with each risk factor corresponding to a line segment, the length of which depicted the magnitude of the factor’s contribution to the ultimate outcome. Each line segment featured a scale, indicating the range of values for each variable. The nomogram comprised the individual scores, denoted as “Point” (first row), which could be derived by making a vertical line upward from each variable. The total score, referred to as “Total Point” (last row), represented the cumulative sum of the individual scores. Then, the predicted death can be obtained by drawing a vertical line downward.

**Figure 4:**
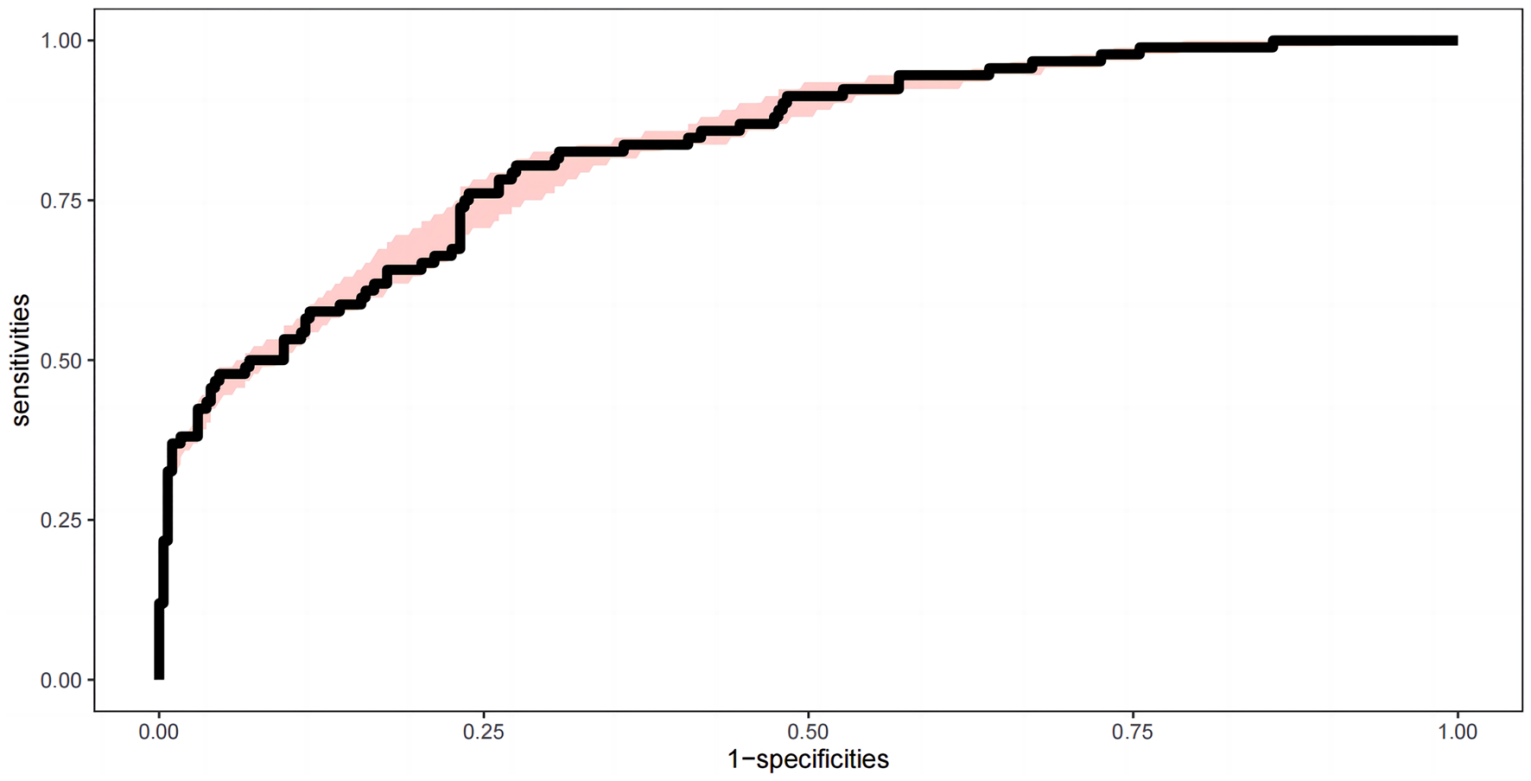
The receiver operating characteristics (ROC) curve of the nomogram for predicting death. The area under curve (AUC) is 0.873 (0.832, 0.915). The pink shading is the confidence interval.

**Figure 5:**
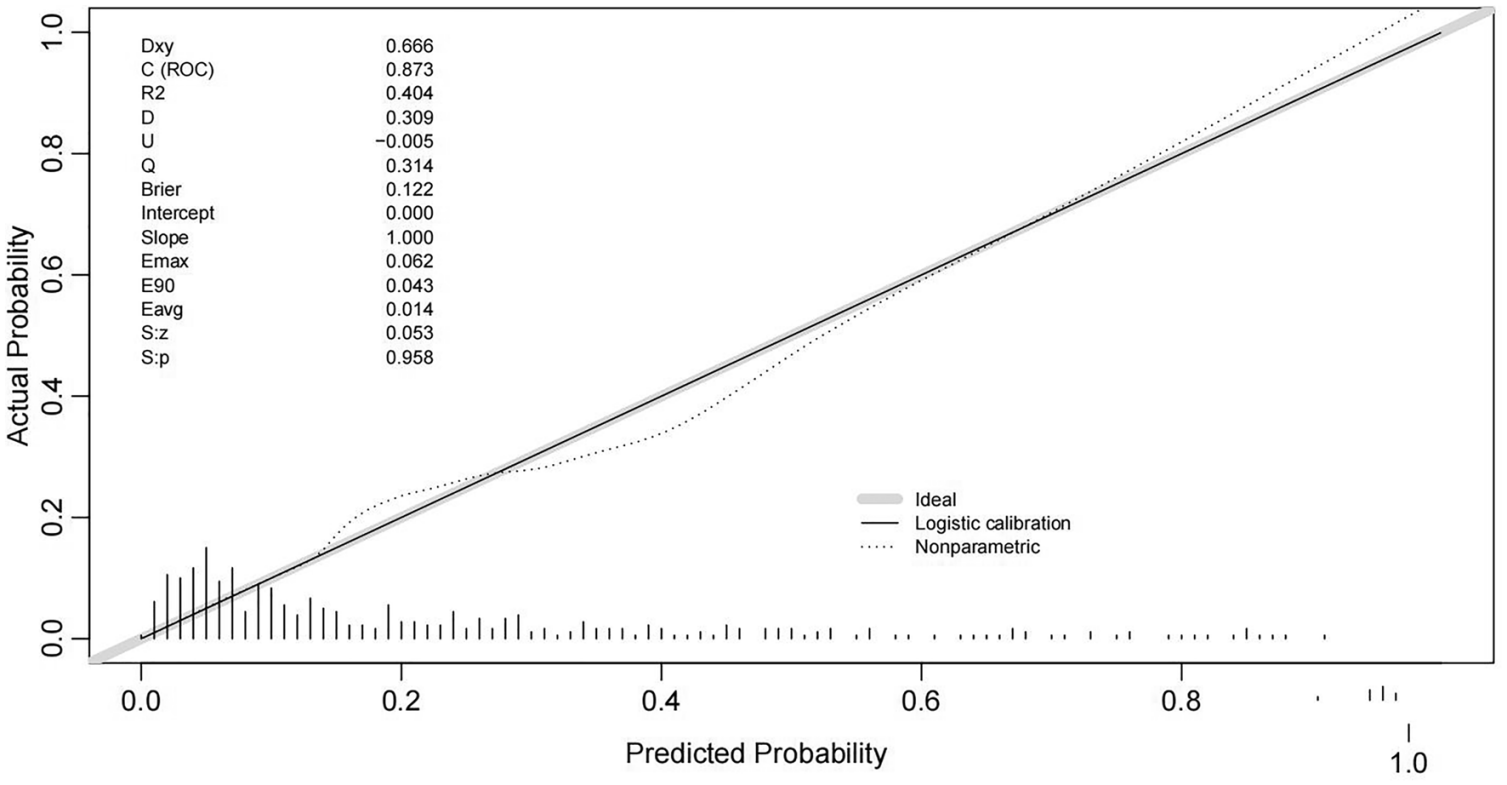
The calibration curve of the nomogram for predicting death.

The AUC was 0.873 (0.832, 0.915), showing a good fit, and the ROC curve with confidence interval was generated (Fig. 4). The results calculated by R indicated that the model passes the calibration test namely Unreliability test with P = 0.958, further affirming the model's effectiveness in predicting outcomes (Fig. 5). The maximum Jorden index was worked out to be 0.606, corresponding to a total score of 91.

## Discussion

All 394 subjects in this study were residents of Weihai City and its surrounding county-level cities located on the Jiaodong Peninsula which is one of the four geographical clusters^16^ where SFTS cases are concentrated from May to October. The cases in our study were in line with its epidemiological characteristics. However, there were also sporadic cases reported in April and November, indicating that the epidemic peak may occur earlier and is more prolonged, which may be related to the increased ambient temperature due to global warming. The phenomenon of global climate warming particularly the increased winter temperature leads to earlier plants growth, initiating an early start of spring ploughing and providing conducive conditions for tick activity. Fang declared in his study that the incidence of SFTS was associated with the area of vegetation- rich lands significantly^17^. A Korean study expounded that ambient temperature ≥ 20 °C was an independent risk factor for patients with positive SFTSV RT-PCR results^18^.

Additionally, only 13.2% of the individuals in our study recalled of a clear history of tick bites and a mere 2.8% had a history of animal contact in the week prior to the onset of the disease. This predicts that others may have been infected through different routes of transmission. It has been proved that the evidence of human-to-animal or human-to-human transmission of SFTSV existed^6,19,20^. The geographical spread of SFTS has expanded beyond Korea and Japan with reported cases in other countries such as Vietnam and Pakistan^21,22^. Remarkably, cases of suspected SFTSV infection have been reported in the United States, a distant overseas location, as early as 2009, called “Heartland virus” at that time^23^.

In this study, SFTS carries a high mortality rate, as high as 23.35% over the decade from 2012 to 2021, which is higher than the 13.04% reported by Enqing You et al.^24^ and lower than the 32.1% reported by Wonsup Oh et al.^25^. As a consequence, a nomogram becomes imperative as it can assist clinicians in rapidly assessing the risk of death and enable timely interventions to reduce the overall mortality rate.

During the construction of the nomogram, we have some findings that can offers new insights into the disease’s pathophysiology.

First of all, we found that mortality was independently associated with advanced age, which aligns with Nie’s findings^26^. Possible reason is that older individuals often experience a natural decline in their immune system function and overall resistance, rendering them more susceptible to viral infection. Additionally, elderly individuals might have a higher likelihood of exposure to ticks through engagement in agricultural activities, which can further exacerbate the disease via dysregulation of host immunocyte and uncontrolled inflammatory reaction^27^. Although there was no significant difference in the underlying disease between the survival and death groups in this study, it's possible that the elderly individuals may have been living with underlying conditions that made it challenging to correct disturbances of the body’s internal environment, leading to death.

We only found significant differences between the death group and the survival group among patients with consciousness disturbance, seizures, and myalgia, although SFTSV infection causes a wide spectrum of clinical symptoms and signs. CNS involvement may arise directly from the neurotropic nature of the virus or indirectly from elevated levels of cytokines resulting in a spectrum of neurological symptoms^28,29^. Regarding myalgia, while our study did not uncover a specific mechanism, it is possible that the virus exhibits muscular tropism, but no supporting reports were found. Although there was no significantly different signs determined on physical examination in our study, previous research by Zhipeng Zu and Feng He suggested that ecchymosis, gingival bleeding and melena were independent risk factors for predicting death^30,31^. These observations may be linked to coagulation disorders, therefore, extra attention should be paid to patients presenting with neurological symptoms or bleeding symptoms.

In the present study, acute liver injury is a significant complication of SFTS.

An autopsy case report revealed the presence of the virus in vital organs such as heart, liver, spleen and bone marrow^32^, suggesting that SFTSV exhibits pantropism which serves as the basis for its ability to infect multiple organs. SFTSV triggers hyperinflammatory response, resulting in a damage in various tissues, cells and further multiple organ systems of human body^33^. As our study found, MODS is an independent risk factor for death, a conclusion that is consistent with findings from a meta-analysis^34^.

A noteworthy discovery from our laboratory tests is that significantly higher viral loads were present in the death group, which consist with a study by Korean scholars^35^. A prospective observational study conducted by Li’s team confirmed viral load as a strong predictor of fatal outcome^36^. The pathological mechanism of SFTSV infection is complex and not elucidated. It involves a cascade of responses from immune cells, immune mediators, inflammasomes, and signaling pathways^37^. However, there is no doubt that cytokine storm plays an pivotal role in disease progression and high viral load can trigger cytokine storm in vivo. Zhong's study demonstrated that deceased patients exhibited high levels of cytokines such as interleukin-6, interleukin-8, interleukin-10, granulocyte colony stimulating factor, interferon-α, which was bound up with high viral load^38^. The release of excessive cytokine can further destroy body tissues and organs, accelerating disease progression. Finally, uncontrollable viremia determined the outcome of death^39^. Hence, Gai advocated that viral load during the MODS phase is a key to predicting the disease outcome as he found that survivors are capable of clearing the virus while conversely those who succumb to the disease still harbor a high viral load^34^.

As anticipated, in our study nearly all laboratory findings were related to fatal outcome in SFTS patients. Elevated ALT and AST implied liver injury, mounted CK-MB, LDH, α-HBDH pointed to myocardial injury, increased urea nitrogen and creatinine indicated acute renal failure, while raised blood potassium were suggestive of disrupted homeostasis. Additionally, decreased platelets, prolonged PT, APTT, TT, along with lowered fib and raised D-dimer suggested impaired coagulation or secondary hyperfibrinolysis. All of theses damages could potentially contribute to patient mortality. It is worth mentioning that thrombocytopenia may result from the adhesion of SFTSV to platelets, subsequently triggering macrophage phagocytosis in the spleen^39^. We found that prolonged APTT and high concentration of D-dimer could be used as independent risk factors for predicting death in SFTS, which have been previously observed in deceased SFTS patients^40^.

The nomogram was plotted based on four independent risk factors associated with death obtained from multivariate binary logistic regression. That were advanced age, MODS, prolonged APTT, elevated D-dimer. The AUC used to measure the discrimination of the prognostic nomogram is 0.873 (0.832, 0.915), which shows a high predictive accuracy for mortality outcomes. The calibration curve further illustrated the predicted probability of nomogram matched up to the actual probability very nicely. Developed using data from patients treated at Weihai Municipal Hospital, this nomogram is particularly put into use for incoming patients at this facility. The prognostic factors it uses are readily available, allowing for the rapid assessment of mortality risk. This facilitates the prompt arrangement corresponding treatment measures to reduced mortality.

Our study was meticulously designed with data collection carried out by medical personnel uniformly trained to ensure consistency and accuracy. The data analysis was conducted using SPSS software, while the nomogram was developed utilizing the R Project. However, this study is Imperfect for lacking external validation, so accumulated future cases are necessary. Additionally, a prospective cohort study is needed to strengthen our findings.

At present, plasma exchange is controversial as a new option to deal with severe viral infections^41^. Inspired by this, we propose a study utilizing our nomogram for risk stratification to determine the appropriateness of plasma exchange therapy. Actually, benefited from the results of the nomogram, a prospective cohort study was designed in January 2022, the objects of which will be segmented into two categories based on the cut-off value of 0.606, a low-risk group and a high-risk group. Distinct clinical treatment strategies including plasma exchange will be implemented for each group. Before that, an external validation for predicted probability of the nomogram will be performed.

## Conclusion

In summary, SFTS has been exhibiting an increasing incidence annually, accompanied by a notably high mortality rate. Our study revealed that the death group exhibited older age and was more prone to consciousness disturbance, seizures/cramp and MODS than the surviving group. Viral load in the deceased patients together with ALT, AST, CK-MB, LDH, α-HBDH and D-dimer were also at a higher level, as well as PT, APTT and TT more prolonged, while fibrinogen lower than in the survival patients. Through multivariate binary logistic regression, we identified four independent mortality risk factors: advanced age, prolonged APTT, elevated D-dimer. Utilizing these factors, we plot a nomogram to calculate the predicted SFTS mortality with good effects. This tool not only aids in clinical decision-making but also laid the methodological groundwork for further research.

### Supplementary Information

Supplementary Information.

## Data Availability

The data that support the findings of this study are available from the supplementary information file.
