# Supplementary material for: A nomogram to predict mortality in patients with severe fever with thrombocytopenia syndrome
Source: Sci Rep. 2024 May 9;14:10627. doi: 10.1038/s41598-024-60923-9 (PMC11081946; doi:10.1038/s41598-024-60923-9)
Supplement: Supplementary file 1 — Supplementary Information. [file 41598_2024_60923_MOESM1_ESM.pdf]

A Nomogram to Predict Mortality in Patients with Severe Fever with Thrombocytopenia Syndrome

Kun Fang*, Xuezhen Song, Jinshuang Bo
Department of Transfusion Medicine, Weihai Municipal Hospital, 264200Weihai, China.

*Corresponding Author:Kun Fang, Attending Physician, Department of Transfusion Medicine, Weihai Municipal Hospital, No.70 of Heping Road, 264200Weihai, Shandong, China.
TEL:+86-15606312986, E-mail:fangkunno1@126.com

Age，y	date	In-hos	Male=1	Farmer	Tick b	Field	contra	Onset	Hypert	Diabet	Cardia	Respir
67	2012/6/13	1	0	0	0	1	0	3	0	0	0	0
41	2012/6/16	0	1	0	0	0	0	5	0	0	0	0
45	2012/7/13	0	0	0	0	1	0	5	0	0	0	0
69	2012/8/1	1	1	1	0	0	0	7	0	0	0	0
48	2012/8/8	0	0	1	1	0	0	8	0	0	0	0
45	2012/8/8	0	1	1	0	0	0	5	0	0	0	0
76	2012/8/13	0	1	1	0	0	0	6	0	0	0	0
72	2012/9/10	1	0	1	1	0	0	10	1	0	0	0
72	2012/10/3	1	0	1	0	0	0	7	0	0	0	0
64	2012/10/9	0	1	0	0	0	0	5	0	0	0	0
68	2013/6/14	1	1	0	1	0	0	5	1	1	0	1
78	2013/6/22	0	0	1	0	0	1	9	0	0	0	0
65	2013/7/1	0	0	0	0	0	0	6	1	1	0	0
73	2013/7/1	0	0	0	0	0	0	10	0	0	0	0
81	2013/7/3	1	1	0	0	0	0	3	1	0	0	1
66	2013/7/8	0	1	1	0	0	1	3	0	0	0	0
51	2013/7/17	0	0	1	0	0	0	10	0	0	0	0
52	2013/7/22	0	0	1	0	0	0	7	0	0	0	0
80	2013/8/9	0	1	0	0	0	0	5	0	0	0	0
69	2013/8/9	0	1	0	1	0	0	5	0	0	0	0
64	2013/8/21	0	1	0	0	0	0	7	1	0	0	0
52	2013/9/9	0	0	0	1	0	0	5	0	0	0	0
78	2013/9/30	1	0	1	1	0	0	7	0	0	0	0
79	2013/10/9	0	1	1	0	0	0	7	0	0	0	0
70	2013/10/16	1	0	1	0	0	0	4	0	0	0	0
68	2013/10/23	1	1	1	0	0	0	6	0	0	0	0
51	2013/10/23	0	1	0	1	0	0	6	0	0	0	0
59	2013/10/24	0	0	1	0	1	0	10	0	0	0	0
53	2013/10/30	0	1	1	0	1	0	3	0	0	0	0
64	2013/10/30	0	0	1	0	0	0	7	0	0	0	0
51	2013/11/24	0	0	0	0	0	1	10	0	0	0	0
65	2013/11/24	1	1	0	0	0	0	6	1	0	1	0
70	2014/5/26	1	0	0	0	0	0	5	0	0	0	0
71	2014/6/3	0	0	1	0	0	0	7	0	0	0	0
71	2014/6/3	0	0	1	0	0	0	10	0	0	0	0
53	2014/6/16	0	0	1	0	0	0	7	0	0	0	0
72	2014/7/21	0	0	1	0	0	0	7	1	1	0	0
67	2014/8/19	0	1	1	0	1	0	7	0	0	0	0
50	2014/8/25	1	1	0	0	1	0	9	0	0	0	0
56	2014/9/22	1	0	0	0	0	0	2	0	0	0	0
48	2014/10/8	0	0	0	0	0	0	4	0	0	0	0
36	2014/10/13	0	1	0	0	0	0	4	0	0	0	0
40	2014/10/13	0	1	0	0	0	0	4	0	0	0	0
65	2015/5/18	0	0	0	0	0	0	5	0	1	0	0
65	2015/5/22	1	1	0	1	0	0	3	0	0	0	0
65	2015/5/25	0	1	0	0	0	0	7	0	0	0	0
63	2015/5/25	1	0	0	0	0	0	6	1	0	1	0
82	2015/6/1	0	0	0	0	0	0	5	0	0	0	0
61	2015/6/1	0	0	0	0	1	0	7	0	0	0	0
50	2015/6/9	0	0	1	0	0	0	7	1	0	0	0

56	2015/6/9	1	1	1	0	0	0	5	0	0	0	0
81	2015/6/9	0	1	0	0	0	0	3	1	0	0	1
78	2015/6/15	1	1	1	0	0	0	6	0	0	0	0
75	2015/6/15	0	1	0	1	0	0	7	0	0	0	0
58	2015/6/15	0	0	1	1	0	0	5	0	0	0	1
71	2015/6/15	0	0	0	0	0	0	6	0	0	0	0
67	2015/6/15	0	1	1	0	0	0	7	0	0	0	1
68	2015/6/15	0	1	0	0	0	0	12	1	0	0	0
62	2015/6/23	0	0	0	0	0	0	3	0	0	0	0
68	2015/6/22	0	0	1	0	0	0	4	1	0	0	0
76	2015/6/22	0	1	1	0	0	0	3	0	0	0	0
73	2015/6/29	0	0	1	0	0	0	2	0	0	0	0
65	2015/7/6	0	1	0	0	0	0	6	1	0	1	0
68	2015/7/6	0	0	1	0	0	0	4	1	0	0	0
64	2015/7/6	1	1	0	0	0	0	5	1	0	0	0
78	2015/7/6	1	1	0	0	0	0	6	0	0	0	1
73	2015/7/13	0	1	0	0	0	0	7	1	1	0	0
49	2015/7/13	0	0	0	0	0	0	3	0	0	0	0
52	2015/7/13	1	0	0	0	0	0	9	0	0	0	0
50	2015/7/20	0	0	0	1	0	0	6	0	0	0	0
78	2015/7/20	1	1	1	0	0	0	4	0	1	0	0
64	2015/7/20	0	0	0	0	0	0	4	1	1	1	0
57	2015/7/27	0	1	0	0	0	0	4	0	0	0	0
75	2015/8/3	0	1	0	0	0	0	5	0	0	0	0
71	2015/8/3	0	0	1	0	0	0	5	0	0	0	0
77	2015/8/3	0	1	1	0	0	0	5	0	0	0	0
44	2015/8/10	0	1	0	0	0	0	10	0	0	0	0
69	2015/8/10	0	1	0	0	0	0	2	0	0	0	0
41	2015/8/10	0	1	1	0	0	0	5	1	0	0	0
51	2015/8/10	0	1	0	0	1	0	4	0	0	0	0
57	2015/8/17	1	1	0	0	0	0	5	0	0	0	0
82	2015/8/17	1	1	0	0	0	0	7	0	0	0	0
70	2015/8/17	1	1	0	1	0	0	10	0	0	0	0
72	2015/8/24	1	0	0	0	0	0	4	0	0	0	0
70	2015/8/24	0	0	0	1	0	0	7	0	0	1	0
64	2015/8/24	0	1	0	1	0	0	3	0	0	0	0
59	2015/8/24	0	1	1	0	0	0	6	0	0	0	0
63	2015/8/31	0	0	0	0	0	0	5	0	0	0	0
60	2015/8/31	1	1	1	0	0	0	7	0	0	0	0
56	2015/9/14	1	0	0	1	0	0	5	0	1	0	0
79	2015/9/14	0	1	0	0	0	0	4	0	0	0	1
64	2015/9/14	1	0	1	1	0	0	10	0	0	0	1
65	2015/9/28	1	0	1	0	0	0	2	0	0	0	0
68	2015/10/7	0	0	0	0	0	0	6	1	0	0	0
79	2015/10/7	1	0	1	0	0	0	4	0	0	0	0
65	2015/10/7	1	0	1	0	0	0	3	1	0	0	0
66	2015/10/7	1	0	0	0	0	0	1	1	0	0	0
65	2015/10/7	0	0	1	0	0	0	10	0	0	0	0
77	2015/10/7	1	1	1	0	0	0	1	0	0	0	0
63	2015/10/7	0	1	1	0	0	0	6	0	0	0	0
63	2016/5/16	0	1	0	0	0	1	7	0	0	0	0

63	2016/5/16	0	1	0	0	0	1	14	0	0	0	0
71	2016/5/23	1	1	0	0	0	0	3	0	0	0	0
64	2016/5/30	0	0	1	0	0	0	3	1	0	0	0
67	2016/5/30	0	1	1	0	0	0	7	1	0	0	0
42	2016/6/6	0	0	0	0	0	0	10	0	0	0	0
60	2016/6/6	0	0	0	0	0	0	5	0	0	0	0
56	2016/6/6	1	0	0	0	0	1	4	0	0	0	1
50	2016/6/13	0	1	0	0	0	0	6	0	0	0	0
58	2016/6/13	0	0	0	0	1	0	4	1	1	0	0
64	2016/6/13	0	1	0	0	0	0	7	1	0	0	0
82	2016/6/13	0	1	0	0	0	0	2	1	1	0	0
81	2016/6/13	0	1	1	0	0	0	7	0	0	0	0
63	2016/6/13	0	0	0	0	0	0	6	0	0	0	0
49	2016/6/20	0	0	0	0	0	0	5	0	0	0	0
75	2016/6/20	1	0	0	0	0	0	4	0	0	0	0
55	2016/6/20	0	0	1	0	0	0	7	1	0	0	0
86	2016/6/20	0	1	1	0	0	1	3	0	0	0	1
54	2016/6/22	0	0	1	0	0	0	8	0	0	0	0
82	2016/7/4	1	0	0	0	1	0	7	0	0	0	0
60	2016/7/4	0	0	0	0	0	0	4	0	0	0	0
62	2016/7/11	0	0	0	0	0	0	5	0	0	0	0
70	2016/7/11	1	0	0	0	0	0	7	0	0	0	0
70	2016/7/11	1	0	0	0	0	0	7	1	1	0	0
63	2016/7/11	0	0	1	0	0	0	6	0	0	0	0
71	2016/7/18	0	1	1	0	0	0	7	1	0	0	0
60	2016/7/18	0	1	0	0	0	0	4	1	0	0	0
68	2016/7/25	0	1	0	0	0	0	2	1	1	1	0
68	2016/8/1	0	1	0	0	0	0	5	0	0	0	0
52	2016/8/1	0	0	1	0	0	0	4	0	0	0	0
78	2016/8/8	1	1	0	0	0	0	7	0	0	1	0
59	2016/8/15	0	1	0	0	0	0	6	0	0	0	0
64	2016/8/15	0	1	0	0	0	0	4	1	0	0	0
74	2016/8/15	0	0	0	0	0	0	3	0	0	0	0
64	2016/8/23	0	0	1	1	0	0	7	1	1	0	0
72	2016/8/23	0	0	0	0	0	0	10	1	1	0	0
82	2016/8/30	1	0	0	0	0	0	6	0	0	0	0
67	2016/9/6	0	1	0	0	0	0	5	1	1	1	0
61	2016/9/6	0	0	0	0	0	0	7	1	0	0	1
59	2016/9/6	0	1	0	0	0	0	3	0	0	0	0
46	2016/9/12	0	0	0	0	0	0	6	0	0	0	0
60	2016/9/12	0	1	0	0	0	0	4	0	0	1	0
59	2016/9/19	0	0	0	0	0	0	7	1	0	0	0
66	2016/9/27	0	1	0	0	0	0	6	1	0	0	0
51	2016/10/11	0	1	0	0	0	1	6	0	0	0	0
39	2016/10/11	0	0	0	0	0	1	2	0	0	0	0
57	2016/10/11	0	0	0	0	0	0	5	0	0	0	0
73	2016/10/11	1	0	0	0	0	0	7	0	1	1	0
64	2016/10/17	1	1	0	0	0	0	5	0	0	0	0
50	2016/10/17	0	1	0	0	0	0	6	0	0	0	0
62	2016/10/17	0	0	1	0	0	0	5	0	0	1	0
49	2016/10/24	0	0	1	0	0	0	3	1	0	0	0

50	2016/10/31	0	1	0	0	0	0	7	0	0	0	0
70	2016/10/31	0	1	1	0	0	0	5	0	1	0	0
60	2016/11/7	0	0	1	0	0	0	6	0	0	0	1
65	2016/11/7	0	1	0	0	1	0	4	0	1	0	1
54	2017/4/24	0	1	0	0	0	0	6	0	0	0	0
51	2017/5/1	0	0	0	0	0	0	7	0	0	0	0
63	2017/5/23	0	0	0	1	0	0	10	1	0	0	0
76	2017/5/23	0	1	0	0	0	0	4	0	0	0	1
66	2017/5/30	0	0	0	0	0	0	11	1	0	1	0
68	2017/5/30	0	1	0	0	0	0	7	0	0	0	0
75	2017/5/30	0	1	0	0	0	0	5	0	0	0	0
67	2017/6/5	0	0	1	0	0	0	4	0	0	1	0
80	2017/6/19	1	0	0	0	0	0	2	1	0	0	0
68	2017/7/3	0	1	0	0	0	0	5	0	0	0	0
64	2017/7/3	0	0	0	0	0	0	7	0	0	1	1
70	2017/7/11	0	1	0	0	0	0	6	0	0	1	0
61	2017/7/17	0	1	0	0	0	0	7	0	0	0	0
60	2017/7/17	0	1	0	0	0	0	6	1	0	0	0
71	2017/7/17	0	1	0	0	0	0	7	0	0	1	0
43	2017/7/24	0	0	1	0	0	0	4	0	0	0	0
60	2017/7/31	0	0	0	0	0	0	3	0	0	0	0
61	2017/8/7	0	0	1	0	0	0	5	1	1	0	0
62	2017/8/7	0	1	0	0	0	0	10	0	0	0	0
76	2017/8/15	1	0	0	0	0	0	1	1	0	0	0
55	2017/8/15	0	0	0	0	0	0	6	0	1	0	0
82	2017/9/4	1	0	0	0	0	0	3	1	1	0	0
72	2017/9/4	0	0	0	0	0	0	6	0	0	0	0
59	2017/9/4	1	0	0	0	0	0	2	0	0	0	1
70	2017/9/11	1	1	0	1	0	0	6	1	0	0	0
66	2017/9/11	0	0	0	0	0	0	5	0	0	0	0
67	2017/9/11	1	1	0	0	0	0	4	1	1	0	0
70	2017/9/18	0	0	0	0	0	0	8	1	0	0	0
63	2017/10/2	0	0	0	0	0	0	5	1	0	0	0
74	2017/10/2	0	0	0	0	0	0	4	0	1	0	0
68	2017/10/2	0	1	0	0	0	0	5	0	1	0	0
65	2017/10/2	1	0	0	0	0	0	6	0	0	0	0
65	2017/10/9	0	1	0	0	0	0	7	0	0	0	0
54	2017/10/16	0	0	0	0	0	0	4	0	0	0	0
82	2017/10/16	1	1	1	0	0	0	10	0	0	0	0
54	2017/10/23	0	0	0	0	0	0	3	0	0	0	0
71	2017/10/23	1	1	0	0	0	0	7	1	1	1	0
68	2017/10/23	0	1	0	0	0	0	1	0	0	0	0
70	2018/5/14	0	0	1	1	0	0	7	0	0	0	0
46	2018/5/14	0	0	1	1	0	0	5	0	0	0	0
77	2018/5/28	0	0	1	0	0	0	5	0	1	0	0
76	2018/5/28	0	0	1	0	0	0	3	1	0	0	0
65	2018/5/28	0	0	1	1	0	0	2	1	1	0	0
88	2018/6/4	0	1	1	0	0	0	3	0	0	0	0
67	2018/6/4	1	0	0	0	0	0	2	0	0	1	0
53	2018/6/11	0	0	1	0	0	0	5	0	1	0	0
62	2018/6/11	0	1	0	0	0	0	5	0	0	0	0

61	2018/6/11	0	0	0	1	0	0	5	0	0	0	0
72	2018/6/11	0	0	0	0	0	0	7	0	1	0	0
54	2018/6/14	0	0	1	0	0	0	5	0	0	0	0
87	2018/6/14	1	1	1	0	1	0	2	0	0	0	0
72	2018/6/18	0	1	1	0	0	0	2	0	0	0	0
67	2018/6/18	0	0	1	0	0	0	4	0	0	0	0
61	2018/6/18	0	0	0	0	0	0	4	1	1	0	0
68	2018/6/21	0	1	0	0	0	1	9	0	0	0	0
71	2018/6/21	0	0	1	0	0	0	6	1	1	0	0
82	2018/6/25	0	1	0	0	0	0	2	0	0	1	0
76	2018/6/28	0	0	1	1	0	0	10	0	0	0	0
60	2018/6/28	0	0	1	0	0	0	5	1	1	0	0
56	2018/6/28	0	1	0	0	1	0	5	1	0	0	0
66	2018/7/2	0	0	1	0	0	0	7	1	0	0	0
66	2018/7/2	1	1	0	0	0	0	6	0	1	0	0
57	2018/7/2	0	0	0	0	0	0	7	0	1	1	1
68	2018/7/19	0	1	1	0	0	0	4	1	0	0	0
61	2018/7/23	0	1	1	0	0	0	6	0	0	0	0
68	2018/7/23	0	0	1	1	0	0	4	0	0	0	0
60	2018/7/26	0	0	1	0	0	0	5	0	1	0	0
68	2018/7/30	1	1	0	0	0	0	4	0	0	0	1
58	2018/7/30	0	1	1	1	0	0	4	0	1	1	0
74	2018/7/30	0	0	1	0	0	0	5	0	0	0	0
73	2018/8/2	1	1	1	0	0	0	7	0	0	0	0
68	2018/8/2	0	1	0	0	0	0	3	0	0	1	0
48	2018/8/6	0	0	1	0	0	0	5	0	0	0	0
63	2018/8/16	1	1	1	1	0	0	4	0	0	0	0
55	2018/8/20	0	0	0	0	0	0	7	1	0	1	0
67	2018/8/20	1	1	1	0	0	0	4	0	0	0	0
63	2018/8/27	0	0	1	0	1	0	2	0	0	0	0
76	2018/8/27	1	1	0	0	0	0	8	0	0	0	1
70	2018/8/30	1	1	0	0	0	0	6	0	0	0	0
63	2018/9/6	0	0	1	1	0	0	3	0	0	0	0
55	2018/9/10	0	1	0	0	0	0	2	0	0	0	0
80	2018/9/14	1	0	0	0	0	0	6	1	1	0	0
47	2018/9/27	0	1	0	0	0	0	4	0	0	0	0
63	2018/9/30	1	1	0	0	0	0	7	1	0	0	0
58	2018/10/11	0	0	1	0	0	0	6	0	0	0	0
56	2018/10/11	0	1	0	0	0	0	1	1	1	0	0
73	2018/10/15	0	0	1	0	0	0	4	0	0	0	0
70	2018/10/15	1	1	1	0	0	0	5	0	0	0	0
62	2018/10/15	0	0	1	0	0	0	6	0	0	0	0
61	2018/10/18	0	1	1	0	1	0	10	0	1	0	0
74	2018/10/29	1	1	0	0	0	0	7	0	0	0	0
58	2018/11/1	0	0	0	1	0	0	4	0	0	0	0
48	2018/11/12	0	0	0	0	0	0	7	0	1	0	0
65	2019/5/13	0	0	0	0	0	0	4	1	0	0	0
68	2019/5/16	0	1	0	0	0	0	5	0	0	0	0
73	2019/5/27	0	1	1	0	0	0	5	0	1	0	0
61	2019/6/17	0	0	1	1	0	0	8	0	0	0	0
56	2019/6/22	0	0	1	0	0	0	2	0	0	0	1

68	2019/6/22	1	1	0	0	0	0	5	0	0	0	0
75	2019/6/26	0	0	1	0	0	0	5	0	0	0	0
81	2019/6/26	0	0	1	0	0	0	3	1	0	1	0
80	2019/6/29	1	0	1	0	0	0	7	1	1	0	0
82	2019/7/1	0	1	0	0	0	0	2	0	0	0	0
74	2019/7/1	1	1	1	0	0	0	3	1	0	0	0
54	2019/7/4	0	0	1	1	0	0	4	1	0	0	0
56	2019/7/4	0	0	1	0	0	0	5	1	0	0	0
56	2019/7/4	0	0	0	0	0	0	3	0	0	0	0
54	2019/7/8	0	0	1	0	0	0	5	0	0	0	0
70	2019/7/15	0	1	1	0	0	0	10	0	0	0	0
68	2019/7/18	1	1	1	0	0	0	5	1	0	0	1
63	2019/8/8	0	1	0	0	0	0	4	0	0	0	0
53	2019/8/10	0	0	0	1	0	0	5	0	0	0	0
64	2019/8/12	0	0	1	0	0	0	4	1	1	0	0
77	2019/8/15	1	1	0	1	0	0	3	0	0	0	0
63	2019/8/17	0	1	0	0	0	0	4	0	0	0	0
54	2019/8/19	0	1	1	1	0	0	3	0	0	0	0
63	2019/8/19	0	1	1	1	0	0	5	0	0	0	0
83	2019/8/19	0	1	1	0	0	0	5	1	0	0	0
71	2019/8/31	0	0	0	0	0	0	2	1	1	0	0
68	2019/9/12	0	0	1	0	0	0	4	1	0	0	0
72	2019/9/19	1	0	1	1	0	0	6	1	0	0	0
68	2019/10/12	0	0	1	0	0	0	3	1	1	0	0
50	2019/10/28	0	0	0	0	0	0	4	0	0	0	0
67	2020/5/4	0	0	0	0	0	0	4	1	1	0	0
68	2020/5/25	0	0	1	0	0	0	5	1	0	0	0
57	2020/5/29	0	1	0	0	0	0	4	0	0	0	0
57	2020/6/5	0	0	0	0	0	0	7	0	0	0	0
57	2020/6/5	1	0	0	1	0	0	1	0	0	0	0
57	2020/6/8	0	1	0	0	0	0	5	0	0	0	0
24	2020/6/10	0	1	0	0	0	0	4	0	0	0	0
57	2020/6/10	0	0	1	0	0	0	5	0	0	0	0
78	2020/6/12	1	1	0	1	0	0	8	0	0	0	0
83	2020/6/15	1	1	0	0	0	0	2	1	0	0	0
66	2020/6/15	0	1	0	0	0	0	4	0	0	0	0
68	2020/6/15	1	1	0	0	0	0	5	1	0	0	0
81	2020/6/17	0	1	1	0	0	0	6	0	0	0	0
80	2020/6/19	0	1	0	0	0	0	5	0	0	0	0
77	2020/6/19	0	1	1	0	0	0	4	0	0	0	0
66	2020/6/22	0	1	1	0	0	0	4	0	0	0	0
84	2020/6/22	0	1	0	0	0	0	3	0	0	0	0
73	2020/6/22	0	0	0	0	0	0	3	1	0	0	0
68	2020/6/22	0	0	0	0	0	0	5	1	0	1	0
73	2020/6/24	0	0	0	0	0	0	3	1	0	0	0
72	2020/6/26	1	0	0	0	0	0	7	0	1	0	0
65	2020/6/29	0	1	1	0	0	0	3	0	0	1	1
59	2020/7/1	0	0	1	0	0	0	6	0	0	0	0
71	2020/7/10	0	0	0	0	0	0	6	0	0	0	0
66	2020/7/17	0	1	0	0	0	0	5	0	0	0	1
72	2020/7/20	0	0	0	0	0	0	1	1	0	0	0

66	2020/7/20	1	0	0	0	0	0	7	0	0	0	0
56	2020/7/27	0	1	0	0	0	0	4	0	0	0	1
63	2020/7/29	0	0	1	0	0	0	8	0	0	0	0
65	2020/8/3	0	0	0	0	0	0	5	1	1	0	0
85	2020/8/7	0	0	1	0	0	0	1	0	0	0	0
88	2020/8/10	0	1	0	0	0	0	5	0	0	0	1
60	2020/8/14	0	0	0	0	0	0	8	0	0	0	0
69	2020/8/17	0	1	0	0	0	0	5	1	0	0	0
57	2020/8/19	0	1	0	1	0	0	4	0	1	1	0
69	2020/8/21	0	1	0	0	0	0	3	1	1	0	0
71	2020/8/24	1	1	0	0	0	0	4	0	0	1	0
73	2020/8/31	0	1	1	0	0	0	7	1	1	0	0
50	2020/9/11	0	1	1	0	0	0	4	1	0	0	0
83	2020/9/16	0	0	0	1	0	0	2	0	0	0	0
63	2020/9/21	1	1	0	1	0	0	4	1	1	0	0
76	2020/9/26	0	1	0	0	0	0	8	0	0	0	0
61	2020/9/28	0	0	0	0	0	0	8	0	0	0	0
70	2020/10/5	0	1	0	0	0	0	7	0	0	0	1
63	2020/10/5	0	0	0	0	0	0	2	0	0	0	0
70	2020/10/12	0	1	0	0	0	0	7	1	1	0	0
71	2020/10/12	0	1	0	0	0	0	7	0	0	1	1
63	2020/10/19	0	0	0	0	0	0	3	0	1	0	0
69	2021-05-26	0	0	0	0	0	0	6	0	1	0	0
73	2021-05-26	0	1	0	0	0	0	5	0	0	0	0
79	2021-05-31	0	1	0	0	0	0	5	0	0	0	0
73	2021-06-16	0	0	0	0	1	0	6	1	1	0	0
77	2021-06-16	1	0	0	0	0	0	5	0	0	0	0
70	2021-06-17	1	1	0	0	0	0	3	0	0	0	0
73	2021-06-17	1	1	0	0	0	0	5	0	0	0	0
83	2021-06-17	0	0	1	0	1	0	5	1	1	0	0
62	2021-06-18	0	0	0	0	0	0	5	0	0	0	0
82	2021-06-19	1	1	1	0	0	0	4	0	0	0	0
65	2021-06-19	0	1	0	0	0	0	4	1	0	0	0
79	2021-06-22	0	0	1	0	0	0	2	0	0	0	0
82	2021-06-25	1	0	0	0	0	0	6	1	0	0	0
67	2021-06-26	0	0	0	0	0	0	5	0	1	0	0
74	2021-06-26	1	0	0	1	0	0	8	1	0	0	0
77	2021-06-28	0	0	0	0	0	0	2	0	0	0	0
68	2021-06-28	0	1	0	0	0	0	6	0	0	1	0
63	2021-06-30	0	1	0	1	0	0	5	1	1	0	0
58	2021-07-02	0	0	0	0	1	0	7	0	0	0	0
71	2021-07-02	0	1	0	0	0	0	4	0	0	0	0
78	2021-07-10	0	0	1	0	0	0	6	1	1	0	0
67	2021-07-24	1	1	0	0	0	0	5	0	0	0	0
78	2021-07-27	0	0	0	0	0	0	6	1	1	0	0
62	2021-07-28	0	0	0	1	0	0	4	0	0	0	1
69	2021-07-28	0	1	0	0	0	0	10	0	0	0	1
70	2021-07-28	0	1	0	1	1	0	4	0	0	0	1
83	2021-08-02	0	0	0	0	1	0	7	0	0	0	0
76	2021-08-03	1	1	0	0	0	0	7	0	0	1	0
66	2021-08-09	0	0	0	1	0	0	5	0	0	0	0

65	2021-08-10	0	0	0	0	0	0	6	0	1	0	0
79	2021-08-13	1	0	1	0	0	0	8	0	0	0	0
68	2021-08-15	1	0	0	0	0	0	5	0	0	0	0
63	2021-08-15	0	0	0	0	0	0	6	0	0	0	0
75	2021-08-16	1	0	0	0	0	0	6	0	0	0	0
65	2021-08-18	1	1	0	0	1	0	7	1	0	1	0
80	2021-08-24	0	1	0	0	0	0	8	0	0	0	0
52	2021-08-31	0	0	0	1	0	0	3	1	0	0	0
82	2021-09-02	0	0	0	1	1	0	8	0	0	0	0
41	2021-09-05	0	1	0	0	0	1	10	0	0	0	0
70	2021-09-08	0	1	0	0	0	0	14	0	0	0	0
69	2021-09-09	0	1	0	1	0	0	3	0	0	0	0
77	2021-09-11	0	0	0	1	0	0	4	1	0	0	0
66	2021-09-14	0	0	0	1	1	0	3	1	0	0	0
50	2021-09-14	0	1	0	0	0	0	3	0	0	0	0
77	2021-09-14	0	1	0	0	0	0	3	0	0	0	0
57	2021-09-27	0	0	0	0	0	0	10	0	0	0	0
74	2021-09-30	0	0	0	0	0	0	3	0	0	0	0
58	2021-10-01	0	0	0	0	1	0	7	0	0	0	0
70	2021-10-04	0	1	0	1	0	0	6	0	0	0	0
77	2021-10-05	0	1	0	0	0	0	7	0	1	0	0
69	2021-10-10	0	0	0	0	0	0	6	1	1	0	0
63	2021-10-10	0	0	0	0	0	0	5	0	0	0	0
59	2021-10-10	0	1	0	0	0	0	7	1	0	0	0
70	2021-10-11	0	0	0	0	0	0	7	0	0	1	0
70	2021-10-13	0	1	1	0	0	0	3	0	0	0	0
76	2021-10-15	0	1	0	0	0	0	6	0	0	0	0
65	2021-10-16	0	1	0	0	0	0	5	0	0	0	0
64	2021-10-16	0	1	0	0	0	0	7	0	0	0	0
62	2021-10-16	0	0	0	0	0	0	7	1	1	0	1
78	2021-10-19	0	1	0	0	0	0	3	1	1	0	0
79	2021-10-22	0	0	0	0	0	0	3	1	1	0	0
66	2021-10-22	1	0	0	0	0	0	6	0	0	0	0
63	2021-10-23	0	0	0	0	0	0	6	1	0	0	0
44	2021-10-24	0	1	0	0	0	0	5	1	1	0	0
51	2021-10-25	0	1	0	0	0	0	8	0	0	0	0
52	2021-10-29	0	0	0	0	0	0	4	0	0	0	0
65	2021-11-14	0	1	1	0	0	0	5	1	0	0	0

Digest	Urolog	In-hos	Tempra	Fever	Fatigu	Chill	Anorex	Nausea	Vomiti	Abdomi	Diarrh	Headac
0	0	1	38.6	1	1	1	0	0	0	1	1	0
0	0	0	38.4	1	1	1	0	1	0	0	1	0
0	0	0	39	1	1	0	0	1	1	1	0	0
0	0	1	39	1	0	0	0	1	1	0	1	0
1	1	0	38	1	1	0	0	1	1	1	0	0
0	0	0	39	1	1	0	0	1	1	1	0	1
0	0	0	38	1	1	0	1	1	1	0	0	0
0	0	1	39	1	0	0	0	1	1	0	0	0
0	0	1	38.5	1	0	0	0	0	0	0	0	0
0	0	0	38.6	1	1	1	0	0	0	0	0	0
0	0	1	39	1	0	0	0	0	0	0	1	0
0	0	0	39	1	0	0	0	0	0	0	0	0
0	0	0	37.7	1	0	0	0	0	0	0	0	0
0	0	0	38.9	1	1	0	0	0	0	0	0	0
0	0	1	38.5	1	1	0	0	0	0	0	0	0
0	0	0	38.5	1	1	0	0	0	0	0	0	0
0	0	0	39.5	1	1	0	0	0	0	0	1	0
0	0	0	39	1	0	0	0	1	1	0	0	0
0	1	0	38.1	1	1	0	0	1	1	0	0	0
0	0	0	39	1	1	0	1	0	0	0	0	0
0	1	0	38.1	1	1	1	1	0	0	0	0	0
0	0	0	39.8	1	1	1	1	0	0	1	1	0
0	0	1	38.8	1	1	0	0	0	0	0	0	0
0	0	0	39	1	1	0	1	1	1	1	1	0
0	0	1	38.8	1	0	0	1	0	0	0	0	0
0	0	1	39	1	0	0	1	0	0	0	0	0
0	0	0	39.2	1	1	1	1	1	0	0	1	0
0	0	0	38	1	1	0	1	0	0	0	1	0
0	0	0	39	1	0	1	0	0	0	0	1	0
0	0	0	39.5	1	1	1	1	0	0	0	0	0
1	0	0	38.9	1	1	1	0	1	1	0	0	0
0	0	1	38.5	1	1	0	0	0	0	0	1	1
0	0	1	39	1	1	1	1	1	0	0	0	0
0	0	0	38.5	1	0	1	0	0	0	0	0	0
0	0	0	39	1	0	1	0	1	1	1	1	0
0	0	0	38.4	1	1	1	0	1	1	0	1	1
0	0	0	39	1	1	1	1	1	0	0	0	0
1	0	0	38.5	1	1	1	1	0	0	0	0	0
0	0	1	39	1	1	1	1	0	0	0	1	0
0	0	1	39.5	1	1	1	1	1	1	1	1	1
0	0	0	39.2	1	1	1	1	0	1	1	0	0
0	0	0	39	1	0	1	0	0	0	0	0	0
0	0	0	39	1	1	1	1	1	0	0	1	0
0	0	0	38.5	1	1	0	1	1	0	0	1	0
0	0	1	40	1	0	1	0	1	1	1	1	0
0	0	0	38.6	1	1	0	0	0	0	1	1	0
0	0	1	39	1	0	0	0	0	0	0	1	0
0	0	0	38.5	1	1	1	1	0	0	0	0	0
0	0	0	38.8	1	1	1	0	0	0	0	0	0
0	0	0	38.8	1	0	1	0	1	0	0	0	0

0	0	1	40	1	1	1	0	0	0	0	1	0
0	0	0	38.5	1	1	1	0	0	0	0	0	0
0	0	1	39	1	1	0	0	0	0	0	0	0
0	0	0	39	1	0	1	0	0	0	0	0	0
0	0	0	38.5	1	1	0	1	1	0	0	1	0
0	0	0	39	1	1	1	1	0	0	0	0	0
1	0	0	39	1	1	1	1	0	0	0	0	0
0	0	0	39	1	0	1	0	0	0	0	0	0
0	0	0	39.1	1	1	0	1	0	0	0	0	1
0	0	0	39	1	0	1	0	1	1	0	0	1
0	0	0	38.6	1	1	1	1	0	0	0	0	0
0	0	0	38.3	1	1	1	1	0	0	0	1	0
1	0	0	38.7	1	0	0	0	0	0	1	1	0
0	0	0	38.8	1	1	1	0	1	0	0	1	0
0	0	1	39	1	0	0	0	0	0	0	0	0
0	0	1	38.5	1	1	1	0	0	0	0	0	0
1	0	0	39	1	1	1	0	0	0	0	0	0
0	0	0	38.6	1	1	1	1	1	1	0	1	0
0	0	1	38.5	1	0	1	0	1	1	0	0	0
0	0	0	39	1	0	0	0	0	0	0	0	1
0	0	1	38	1	1	0	1	1	0	0	0	0
0	0	0	38.5	1	0	1	0	0	0	0	0	1
1	0	0	38.4	1	1	1	1	0	0	0	1	1
0	0	0	38.5	1	1	1	1	1	1	0	0	0
0	0	0	39	1	1	0	1	1	0	0	0	0
0	0	0	38	1	1	0	1	1	0	0	0	0
0	0	0	39.5	1	1	1	0	0	0	0	1	1
0	0	0	38.7	1	1	0	1	1	1	0	1	0
0	0	0	39.4	1	1	1	1	0	0	0	0	0
0	0	0	39.5	1	1	1	0	0	0	0	0	1
1	0	1	39.6	1	0	1	0	1	0	1	1	0
0	0	1	38.5	1	1	0	1	1	0	0	0	0
0	0	1	38.7	1	1	0	1	0	0	0	1	0
1	0	1	38.4	1	0	0	0	0	0	1	0	0
0	0	0	38.6	1	1	0	1	0	0	0	1	0
0	0	0	39.5	1	1	1	1	0	0	0	0	0
1	0	0	38.5	1	0	0	1	0	1	0	1	0
1	0	0	39	1	1	0	0	1	1	0	1	0
0	0	1	39	1	1	1	1	0	0	0	1	0
0	0	1	38.5	1	1	1	1	0	0	0	0	0
1	0	0	39.5	1	1	1	1	1	1	0	0	0
0	0	1	38.5	1	0	1	0	1	1	0	0	0
0	0	1	39.3	1	1	0	1	1	0	0	1	0
0	0	0	39.2	1	1	1	0	0	0	0	0	0
0	0	1	38.6	1	1	1	1	1	0	0	0	0
0	0	1	39.1	1	1	0	1	0	1	0	1	0
0	0	1	38.6	1	1	1	1	1	1	0	0	0
0	0	0	38	1	1	0	1	0	0	0	0	0
0	0	1	38.3	1	1	0	1	0	0	0	0	0
0	0	0	39	1	1	1	0	1	1	0	0	0
0	0	0	38.1	1	1	1	1	0	0	0	0	0

0	0	0	38	1	0	0	0	1	1	0	1	0
0	0	1	38.6	1	1	1	1	0	0	0	1	0
0	0	0	39	1	1	1	1	1	0	0	1	0
0	0	0	38	1	1	1	1	1	0	0	0	0
0	0	0	38.9	1	0	0	0	0	0	0	0	0
0	0	0	38.6	1	1	0	1	0	1	0	0	0
0	0	1	39	1	1	1	1	0	0	0	1	0
0	0	0	39	1	1	0	1	0	0	0	1	0
0	0	0	38.5	1	0	1	0	1	1	0	0	0
0	0	0	38.5	1	0	0	0	0	0	0	1	0
0	0	0	39	1	0	0	0	0	0	0	0	0
0	0	0	38.5	1	0	0	0	1	1	0	0	0
0	0	0	38.3	1	1	1	1	1	0	0	1	0
0	0	0	39	1	1	1	1	1	0	0	0	0
1	0	1	38.9	1	1	0	1	1	0	0	0	0
0	0	0	39	1	1	1	1	1	0	1	0	1
0	0	0	38.2	1	0	1	0	0	0	0	1	1
0	0	0	38.5	1	1	0	1	0	0	0	1	0
0	0	1	38.5	1	0	1	0	1	1	1	0	0
0	0	0	39.4	1	1	1	1	0	0	1	1	0
0	0	0	39.7	1	1	1	0	1	0	1	1	0
0	0	1	38.4	1	1	0	1	1	1	0	0	0
0	0	1	38.6	1	1	1	1	1	1	1	1	0
0	0	0	38.9	1	0	1	0	0	1	0	0	1
0	0	0	38.5	1	1	0	0	0	0	0	1	0
0	0	0	39	1	1	0	1	0	0	0	0	0
0	0	0	38.6	1	0	0	0	0	0	0	1	0
0	0	0	38.6	1	1	0	1	0	0	0	0	0
0	0	0	38.3	1	1	0	1	0	0	0	0	0
0	0	1	39	1	1	1	1	0	0	0	0	0
0	0	0	38.5	1	1	0	1	0	0	0	1	0
0	0	0	39.5	1	0	0	0	0	0	1	1	0
0	0	0	39.5	1	1	0	0	0	0	0	0	0
0	0	0	38.2	1	1	1	0	1	1	0	0	0
0	1	0	39	1	0	0	0	0	0	0	0	0
0	0	1	38.3	1	0	0	0	0	0	0	1	0
0	0	0	38.5	1	1	0	1	0	0	1	0	0
0	0	0	38.7	1	1	1	0	1	1	0	1	0
0	0	0	39	1	1	1	1	0	0	0	0	0
0	0	0	37.8	1	1	0	1	0	0	0	0	0
0	0	0	38	1	1	1	0	0	0	1	1	0
0	0	0	38.6	1	1	0	1	1	0	0	0	0
0	0	0	39	1	0	0	0	1	1	0	1	0
0	0	0	39.3	1	1	1	1	1	0	0	1	0
0	0	0	39	1	1	1	1	0	0	0	0	1
0	0	0	39.2	1	0	1	0	1	0	0	0	0
0	0	1	38.4	1	0	0	0	0	0	0	0	0
0	0	1	38.9	1	1	1	0	0	0	0	1	0
0	0	0	39	1	1	1	1	0	0	0	0	0
0	0	0	38.2	1	1	0	1	0	0	1	0	0
0	0	0	39	1	0	1	0	0	0	0	0	1

0	0	0	37.8	1	1	1	1	1	1	0	0	0
0	0	0	38.6	1	0	1	0	1	1	0	1	0
0	0	0	38	1	0	1	0	1	0	0	0	0
0	0	0	38.5	1	0	1	0	0	0	0	0	1
0	0	0	38	1	1	1	0	0	0	1	1	0
0	0	0	38.5	1	1	1	0	0	0	0	1	1
0	0	0	40	1	0	0	0	0	0	0	0	0
0	0	0	38.5	1	1	0	0	1	1	0	0	0
0	0	0	38.3	1	1	0	1	1	0	0	0	0
0	0	0	39	1	1	0	1	1	1	0	0	1
0	0	0	39	1	1	1	0	1	1	0	1	0
0	0	0	38.5	1	0	1	0	0	0	0	0	0
0	0	1	39	1	0	0	0	0	0	0	1	1
1	0	0	38.6	1	1	0	1	0	0	0	0	0
0	0	0	39	1	0	0	0	0	0	1	1	0
0	0	0	39	1	1	0	0	0	0	0	1	0
0	0	0	39	1	1	1	0	1	0	0	1	0
1	0	0	39	1	1	1	0	1	1	0	1	0
0	0	0	38.9	1	1	1	0	1	1	0	0	0
0	0	0	38.5	1	1	0	0	0	0	0	0	0
0	0	0	38	1	0	0	0	0	0	0	1	0
0	0	0	39.9	1	1	0	0	0	0	0	0	0
0	0	0	39	1	1	0	1	1	1	1	1	0
0	0	1	38.2	1	1	1	0	1	0	1	1	0
0	0	0	39	1	1	0	1	0	0	0	0	0
0	0	1	38.7	1	1	1	1	0	0	0	1	0
0	0	0	39	1	0	1	0	1	1	0	1	0
0	0	1	39	1	1	1	0	1	1	0	0	0
0	0	1	38.4	1	0	0	0	0	0	0	1	0
0	0	0	40	1	1	1	1	1	1	0	1	0
0	0	1	38.7	1	1	0	0	0	0	0	0	0
0	0	0	39.5	1	1	1	0	0	0	0	0	1
0	0	0	38.7	1	1	1	1	1	1	0	0	0
0	0	0	39.5	1	0	1	0	0	0	0	1	0
0	0	0	38.5	1	0	0	0	1	0	0	1	1
1	0	1	39	1	0	1	0	0	0	0	0	0
1	0	0	39	1	0	1	0	1	1	0	0	0
1	0	0	38.1	1	1	0	0	0	0	0	0	0
0	0	1	38.1	1	1	1	0	0	0	0	1	0
0	0	0	39	1	1	1	0	0	0	0	0	0
0	0	1	38.6	1	1	0	1	0	0	0	0	0
1	0	0	38	1	0	1	0	1	1	1	1	0
1	0	0	38.2	1	1	0	0	1	1	0	0	0
0	0	0	39	1	1	1	0	1	1	0	0	0
0	0	0	39.4	1	1	1	0	0	0	0	0	0
0	0	0	38.5	1	1	1	0	1	1	0	0	0
0	0	0	39	1	1	1	1	1	0	0	0	0
0	0	0	39	1	1	1	0	0	0	0	0	0
0	0	1	38.2	1	1	0	1	1	0	0	0	0
0	0	0	39	1	1	0	0	0	1	0	0	1
0	0	0	39	1	1	0	0	1	1	0	1	1

0	0	0	38.3	1	1	0	0	0	0	0	1	0
0	0	0	38.5	1	0	0	0	0	0	0	1	1
0	0	0	39	1	1	1	0	0	0	0	0	0
0	0	1	39	1	0	1	0	0	1	0	1	0
0	0	0	38.8	1	1	0	0	1	1	0	1	0
0	0	0	38.7	1	1	1	0	0	0	1	0	1
0	0	0	38.5	1	1	1	0	1	0	0	0	0
0	0	0	38.3	1	1	1	0	0	0	0	0	0
0	0	0	38.4	1	1	1	0	1	1	0	0	0
1	0	0	38.6	1	1	0	1	0	0	0	1	0
0	0	0	38	1	1	1	0	1	1	0	1	0
0	0	0	38.5	1	0	1	0	0	0	0	1	0
0	0	0	38.7	1	1	0	0	0	0	0	0	0
0	0	0	38	1	1	0	0	1	0	0	0	0
0	0	1	40	1	0	0	0	0	0	0	0	0
1	0	0	39.5	1	1	1	1	1	0	0	1	0
0	0	0	38.2	1	1	1	1	1	0	0	1	0
0	0	0	39.5	1	1	0	1	1	0	0	1	0
0	0	0	39	1	1	1	0	0	0	0	1	0
0	0	0	39	1	1	1	0	1	1	0	1	0
0	0	1	38.5	1	1	1	1	0	0	1	1	0
0	0	0	39.2	1	1	0	0	1	1	0	0	0
1	0	0	39	1	1	0	0	1	1	0	1	0
0	0	1	39.5	1	1	1	1	0	0	0	0	0
0	0	0	38.5	1	1	0	1	1	1	0	1	1
0	0	0	38.2	1	1	0	1	0	0	0	1	0
1	0	1	38	1	1	0	1	0	0	0	0	1
0	0	0	38	1	1	0	1	0	0	0	0	0
0	0	1	39	1	1	0	0	1	1	0	0	0
0	0	0	37.9	1	0	0	0	1	0	0	0	0
1	0	1	38.3	1	0	1	0	0	1	0	1	0
0	0	1	38.5	1	1	0	0	0	0	0	1	0
0	0	0	38.5	1	1	1	1	0	0	0	0	1
0	0	0	39	1	1	1	1	0	0	0	1	0
1	0	1	39.5	1	1	1	1	0	1	0	1	0
0	0	0	39.6	1	1	1	1	0	0	0	0	0
0	0	1	38.5	1	0	1	0	0	0	0	1	0
0	0	0	38.5	1	1	1	1	1	0	0	0	0
1	0	0	38.6	1	0	0	0	1	1	1	1	0
0	0	0	38.7	1	1	1	1	1	1	0	0	1
1	0	1	39	1	0	1	0	1	0	0	1	0
0	0	0	39.4	1	1	1	1	1	0	0	1	0
0	0	0	39.5	1	0	1	0	0	0	0	1	0
0	0	1	38.7	1	1	0	0	1	1	0	0	0
0	0	0	38.5	1	0	1	1	0	0	1	0	0
0	0	0	38.6	1	1	0	1	0	0	0	0	0
0	0	0	38.5	1	1	0	1	1	0	0	0	1
1	0	0	39.2	1	1	1	1	1	1	0	0	1
1	0	0	38.5	1	0	0	0	1	1	0	0	0
0	0	0	38	1	1	1	0	1	0	0	1	0
0	0	0	39	1	1	1	0	0	0	0	0	0

1	0	1	38.4	1	1	0	1	1	0	0	0	0
0	0	0	38.8	1	1	0	1	1	1	0	0	1
0	0	0	38.8	1	1	1	0	0	0	1	0	0
1	0	1	39.5	1	1	1	1	1	1	0	0	0
0	0	0	38.5	1	1	1	1	0	0	0	1	0
0	0	1	39	1	1	1	0	0	0	1	0	0
1	0	0	39.5	1	1	1	0	1	1	0	0	0
0	0	0	38.5	1	0	1	0	1	0	1	0	0
0	0	0	40	1	1	0	1	0	0	0	0	0
0	0	0	39.5	1	1	1	1	1	1	1	0	0
0	0	0	39	1	1	1	1	1	0	0	0	0
0	0	1	37.8	1	1	0	0	0	0	0	1	0
0	0	0	38.5	1	1	0	1	0	0	0	0	0
0	0	0	39.5	1	1	1	0	1	1	0	1	0
0	0	0	38.5	1	0	0	0	0	0	0	1	0
0	0	1	38.2	1	1	1	1	0	0	0	0	0
0	0	0	39	1	0	0	0	1	1	0	1	0
0	0	0	38	1	1	0	0	0	0	0	0	0
0	0	0	39.5	1	0	1	0	1	1	0	1	0
0	0	0	38	1	1	0	0	0	0	0	0	0
0	0	0	38.2	1	1	0	1	1	1	0	0	0
1	0	0	38.1	1	1	0	0	0	0	0	0	0
1	0	1	39	1	0	0	0	1	1	0	0	0
1	0	0	38.5	1	1	0	0	0	0	0	0	0
0	0	0	37.8	1	1	1	0	1	1	0	1	0
0	0	0	39	1	1	0	1	1	1	0	1	1
0	0	0	38.2	1	0	1	0	0	0	0	0	0
0	0	0	39	1	0	1	0	0	0	0	0	1
0	0	0	39	1	0	1	1	0	0	0	0	1
0	0	1	39	1	1	0	1	1	1	0	0	1
0	0	0	38.9	1	1	0	1	0	0	0	1	0
0	0	0	39.4	1	0	0	0	0	0	0	0	0
1	0	0	38.8	1	1	0	1	1	0	0	0	0
0	0	1	38	1	1	1	1	0	0	0	0	0
0	0	1	38.9	1	1	0	1	0	0	0	0	0
0	0	0	38.3	1	0	0	0	1	0	1	0	0
0	0	1	38	1	0	0	1	0	0	0	0	0
0	0	0	39.4	1	1	0	0	0	0	0	0	0
1	0	0	38.3	1	1	0	0	0	0	0	1	0
0	0	0	38.4	1	1	0	1	0	0	0	1	0
0	0	0	40	1	0	1	0	1	1	0	0	0
0	0	0	38	1	1	1	1	1	1	0	0	1
0	0	0	37.8	1	0	1	0	0	0	0	1	0
0	0	0	39	1	0	0	0	0	0	1	0	1
0	0	0	38.8	1	0	1	0	1	1	0	0	1
0	0	1	38.4	1	1	0	1	1	1	0	0	0
0	0	0	38	1	1	0	1	1	1	0	1	0
0	0	0	37.8	1	1	0	1	1	0	1	1	0
1	0	0	38.6	1	1	1	1	1	0	0	1	0
0	0	0	39.6	1	0	1	0	0	0	0	1	1
0	0	0	37.8	0	1	1	1	0	0	0	0	0

0	0	1	38	1	1	0	1	1	1	0	1	0
0	0	0	38.8	1	0	0	0	1	1	1	0	0
0	0	0	38.5	1	1	1	0	0	0	1	1	0
0	0	0	39.5	1	1	1	1	1	0	0	1	1
0	0	0	38	1	1	0	0	1	1	0	1	0
0	0	0	38.2	1	0	1	0	0	0	0	0	1
0	0	0	38	1	0	1	0	0	0	0	1	0
1	0	0	39	1	1	1	1	0	0	0	1	1
0	0	0	38	1	1	1	0	0	0	0	1	0
0	0	0	38.9	1	1	1	1	1	1	0	1	0
0	0	1	37.7	1	1	1	1	0	0	0	0	1
1	0	0	38.2	1	1	1	1	0	0	0	1	0
1	0	0	38.9	1	1	1	1	0	0	0	0	0
1	0	0	39.2	1	0	0	0	1	0	0	0	0
1	0	1	38.4	1	1	1	1	0	0	0	0	0
0	0	0	39.4	1	0	1	0	1	0	0	1	0
0	0	0	38.2	1	1	1	1	1	0	0	0	0
0	0	0	37.7	1	1	1	0	0	0	0	0	0
0	0	0	38.6	1	1	0	1	1	1	0	0	0
0	0	0	38.5	1	0	1	0	0	0	1	1	0
0	0	0	38	1	1	0	0	0	0	0	0	0
1	0	0	40	1	1	0	1	0	0	0	0	0
0	0	0	39.5	1	0	0	0	0	0	0	0	0
0	0	0	38	1	1	0	1	1	1	0	1	0
0	0	0	39	1	1	1	0	0	0	0	1	1
0	1	0	39	1	1	1	0	0	0	0	1	0
0	1	1	38	1	1	0	0	0	1	0	1	0
0	0	1	39	1	1	1	0	1	0	0	0	0
1	0	1	38.3	1	0	1	0	0	1	0	0	0
0	0	0	38.5	1	1	0	1	1	1	0	0	0
0	0	0	38.6	1	1	0	1	0	0	0	0	0
0	0	1	38	1	1	0	1	0	0	1	1	0
0	0	0	37.8	1	1	0	1	0	0	0	0	0
0	0	0	39.2	1	1	1	1	0	0	0	1	0
0	0	1	38	1	0	0	0	1	1	0	1	0
0	0	0	39	1	1	0	0	1	1	0	0	0
0	0	1	39.1	1	1	0	1	0	1	0	1	0
0	0	0	38.6	1	0	1	1	0	1	0	1	0
0	0	0	38.8	1	1	0	1	0	0	0	0	0
0	0	0	37.8	1	0	0	0	1	1	0	0	0
0	0	0	39	1	0	1	0	0	0	0	1	0
0	0	0	38.5	1	0	0	0	1	1	0	0	0
0	0	0	37.6	1	1	0	0	0	0	0	1	0
0	0	1	39.3	1	1	0	1	0	0	0	0	0
0	0	0	38	1	1	0	1	1	1	0	0	0
0	0	0	38.8	1	1	0	1	1	1	1	1	0
0	0	0	38.8	1	1	0	1	0	0	0	0	0
0	0	0	38.5	1	1	0	0	0	0	0	0	0
0	0	0	38.9	1	1	0	1	1	1	0	0	0
0	0	1	38.8	1	0	0	1	0	0	0	1	0
0	0	0	38	1	1	0	1	1	1	0	0	0

0	0	0	38.2	1	1	0	1	0	0	0	1	0
0	0	1	39	1	1	0	1	0	0	0	0	1
0	0	1	39	1	1	0	1	0	0	0	1	0
0	0	0	39	1	0	0	1	0	0	0	0	0
0	0	1	39.2	1	0	0	1	0	1	1	1	0
0	0	1	38	1	1	0	1	0	0	0	1	0
0	0	0	37.8	1	1	1	1	0	0	1	1	0
0	0	0	39	1	1	0	0	0	0	0	1	0
0	0	0	39	1	1	0	1	0	0	0	0	0
0	0	0	38	1	1	0	0	0	0	0	0	1
0	0	0	38.8	1	1	1	0	0	0	0	1	0
0	0	0	38.5	1	1	0	1	1	1	0	0	0
0	0	0	38.8	1	1	1	1	0	0	0	0	0
0	0	0	39	1	1	0	0	1	1	0	0	0
0	0	0	39	1	1	1	1	0	0	0	0	0
0	0	0	38.2	1	1	0	1	1	0	0	0	0
0	0	0	38.3	1	1	1	0	0	0	0	0	0
1	0	0	39	1	1	1	1	0	0	0	0	0
0	0	0	38.5	1	1	1	1	0	0	0	0	0
0	0	0	38	1	1	0	1	0	0	0	1	0
0	0	0	38.5	1	1	1	1	0	0	0	1	0
0	0	0	38.9	1	0	1	1	0	0	1	0	0
1	0	0	38.5	1	0	1	1	0	0	0	0	0
0	0	0	38.6	1	1	0	1	0	0	0	1	0
0	0	0	38	1	1	1	1	0	0	0	0	0
0	0	0	38.7	1	0	1	0	0	0	0	0	0
0	0	0	39	1	1	0	1	1	1	0	1	0
0	0	0	38	1	1	0	1	0	0	0	1	0
0	0	0	39.5	1	1	0	1	0	0	0	1	0
0	0	0	39.5	1	1	0	0	1	1	1	1	0
0	0	0	38.2	1	1	0	1	0	0	0	0	0
0	0	0	39	1	1	1	1	1	0	0	0	1
0	0	1	38	1	1	0	1	0	0	1	0	0
0	0	0	39	1	1	1	1	1	1	0	1	0
0	0	0	38.5	1	1	1	0	0	0	0	0	0
0	0	0	38.8	1	1	1	1	0	0	0	1	0
0	0	0	39	1	1	1	1	0	0	0	0	0
0	0	0	38	1	1	0	1	1	1	0	1	0

Dizzy	Mental	Seizur	Myalgi	Muscle	Cough/	bleedi	Lympha	Acute	Lung r	Abdomi	Percus	Bloods
1	1	1	0	0	0	1	0	1	0	0	0	0
0	1	0	1	0	0	0	0	1	0	0	0	0
0	0	0	1	0	0	0	0	1	0	0	0	0
0	0	1	0	0	0	1	0	0	0	0	0	0
0	0	0	0	0	0	1	0	0	0	0	0	0
1	1	0	1	0	0	1	0	0	0	0	0	0
0	0	0	0	0	0	0	0	0	0	0	0	0
1	1	0	0	1	1	1	1	1	0	0	0	0
0	1	0	0	1	1	1	1	0	0	0	0	0
0	0	0	1	1	1	1	1	0	0	0	0	0
0	1	0	0	1	0	0	0	0	0	0	0	0
0	1	0	0	0	0	0	0	0	0	0	0	0
1	0	0	0	0	0	0	0	0	0	0	0	1
0	0	0	0	0	0	0	0	0	0	0	0	0
0	1	0	0	1	0	0	0	0	0	0	0	0
0	1	0	0	0	1	0	0	0	0	0	0	0
0	0	0	0	1	0	0	0	0	0	0	0	0
1	0	0	0	0	0	0	0	0	0	0	0	0
0	0	0	0	0	0	0	0	0	0	0	0	0
0	1	0	0	0	0	0	0	0	0	0	0	0
0	0	0	0	0	0	0	0	0	0	0	0	0
1	0	0	0	0	0	1	0	0	0	0	0	0
1	0	0	0	0	0	0	1	0	0	0	0	0
0	1	0	0	0	0	0	0	0	0	0	0	0
0	0	0	0	0	0	0	1	0	0	0	0	0
0	1	0	0	0	0	0	0	0	0	0	0	0
0	0	0	0	0	0	0	0	0	0	0	0	0
0	0	0	0	1	1	0	1	0	0	0	0	0
1	0	0	0	0	0	0	1	0	0	0	0	0
0	0	0	1	0	0	0	0	0	0	0	0	0
0	0	0	1	0	1	0	0	0	0	0	0	0
0	0	0	1	0	0	0	0	0	0	0	0	0
0	0	0	0	0	0	0	0	0	0	0	0	0
0	0	0	1	0	0	0	0	0	0	0	0	0
0	0	0	0	1	0	0	0	0	0	0	0	0
0	0	0	1	0	0	0	1	0	0	0	0	0
0	0	0	0	1	0	0	1	0	0	0	0	0
0	0	0	0	0	0	0	0	0	0	0	0	0
0	0	0	0	0	0	0	1	0	0	0	0	0
1	0	0	0	0	0	1	0	1	0	0	0	0
0	0	0	1	0	0	0	0	0	0	0	0	0
0	0	0	0	0	0	0	0	0	0	0	0	0
0	0	0	1	0	0	0	0	0	0	0	0	0
0	1	0	0	1	0	1	0	0	0	0	0	0
1	0	0	0	0	0	0	0	1	0	0	0	0
0	0	0	0	0	0	0	1	0	0	0	0	0
0	1	1	0	0	0	0	0	0	0	0	0	0
0	1	0	1	0	1	0	0	0	0	0	0	0
0	0	0	0	0	0	0	1	0	0	0	0	0
0	0	0	1	0	1	0	0	0	0	0	0	0

0	0	0	1	0	0	0	1	0	0	0	0	0
0	0	0	0	0	1	0	0	0	0	0	0	0
0	1	0	0	1	1	0	1	0	1	0	0	0
0	0	0	0	0	0	0	1	1	0	0	0	0
0	1	0	1	0	0	0	0	0	0	0	0	0
0	0	0	1	0	0	0	0	0	0	0	0	0
0	0	0	0	0	0	0	0	0	0	0	0	0
0	0	0	1	0	1	0	1	0	0	0	0	0
0	0	0	1	0	0	0	0	0	0	0	0	0
1	0	0	1	0	1	0	1	0	0	0	0	0
1	0	0	0	0	0	0	0	0	1	0	0	0
1	0	0	0	1	1	0	1	0	1	0	0	0
0	0	0	0	0	0	0	1	0	0	0	0	0
0	1	0	0	0	0	0	1	0	1	0	0	0
0	1	0	0	0	1	0	0	0	0	0	0	0
0	1	0	0	0	1	0	0	0	0	0	0	0
0	0	0	1	0	0	0	0	0	1	0	0	0
0	0	0	0	0	0	0	1	0	0	0	0	0
0	0	0	0	0	0	0	1	0	1	0	0	0
0	0	0	1	0	1	0	1	0	1	0	0	0
1	0	0	0	1	1	0	0	0	1	0	0	0
1	0	0	0	0	0	0	0	0	1	1	0	0
0	0	0	1	0	0	0	1	0	1	0	0	0
0	0	0	1	0	0	0	1	0	0	0	0	0
1	0	0	0	0	0	0	0	0	0	0	0	0
1	0	0	0	0	0	0	0	0	0	0	0	0
0	0	0	1	0	0	0	1	0	0	0	0	0
0	1	0	1	1	0	0	0	0	0	0	0	0
1	0	0	1	0	0	0	0	0	0	0	0	0
1	0	0	1	0	0	0	1	0	1	0	0	0
0	0	0	0	0	0	0	1	0	0	0	0	0
0	0	0	0	0	0	0	0	0	0	0	0	0
0	1	0	0	0	0	0	1	0	1	0	0	0
0	0	0	1	0	0	0	0	0	0	0	0	0
1	0	0	0	0	0	0	1	0	0	0	0	0
0	0	0	1	0	0	0	1	0	0	0	0	0
0	0	0	0	1	0	0	1	0	0	0	0	0
1	0	0	1	0	0	0	1	0	1	0	0	0
0	1	0	0	0	0	0	1	0	1	0	0	0
0	0	0	0	0	0	0	1	0	0	0	0	1
0	0	0	1	0	0	1	1	0	1	0	0	1
0	0	0	1	0	1	0	1	0	1	0	0	0
0	1	0	0	1	0	1	1	0	0	0	0	0
0	0	0	1	0	0	0	1	0	1	0	0	0
0	1	0	0	0	0	0	0	0	0	0	0	0
0	1	0	0	1	1	0	1	0	0	0	0	0
0	0	0	0	0	0	0	0	0	0	0	0	0
0	0	0	0	0	0	0	1	0	1	0	0	0
0	0	0	1	1	0	0	1	0	0	0	0	0
0	1	0	1	0	0	0	1	0	0	0	0	0
1	0	0	1	0	0	0	1	0	1	0	0	0

0	0	0	0	1	0	0	1	0	0	0	0	0
0	1	0	0	0	0	0	1	0	0	0	0	0
0	0	0	0	1	0	0	1	0	0	0	0	0
0	1	0	0	1	0	0	0	0	1	0	0	0
0	1	0	0	1	0	1	1	0	0	0	0	0
1	0	0	1	1	0	0	1	0	1	0	0	0
0	0	0	0	0	0	0	0	0	0	0	0	0
0	0	0	0	0	0	0	1	0	0	0	0	0
0	0	0	1	0	0	0	0	1	0	0	0	0
0	1	0	0	1	0	0	0	0	0	0	0	0
0	0	0	0	0	0	0	0	0	0	0	0	0
0	0	0	0	1	0	0	1	0	0	0	0	0
0	0	0	0	1	0	0	1	0	0	0	0	0
1	0	0	0	0	1	0	1	0	1	0	0	0
0	0	0	0	0	0	1	1	0	0	0	0	0
1	0	0	0	1	0	0	1	0	0	0	0	0
0	1	0	1	0	1	0	0	0	1	0	0	0
0	0	0	0	1	0	0	1	0	1	0	0	0
0	0	0	0	0	0	0	0	0	0	0	0	0
0	0	0	1	0	0	0	1	0	0	0	0	0
0	0	0	0	1	0	0	1	0	0	1	0	0
0	1	0	0	1	0	0	0	0	0	0	0	0
0	1	0	1	1	0	0	0	0	0	0	0	0
0	0	0	0	0	0	0	0	0	0	0	0	0
0	0	0	0	0	0	0	0	0	0	0	0	0
0	0	0	0	1	0	0	1	0	1	0	0	0
0	0	0	0	0	0	0	0	0	0	0	0	0
0	0	0	1	0	0	0	1	0	1	0	0	0
0	0	0	1	0	0	0	1	0	0	0	0	0
0	1	0	0	1	0	0	1	0	0	0	0	0
0	0	0	1	0	0	0	0	0	0	0	0	0
1	0	0	0	0	0	0	1	0	1	0	0	0
0	0	0	0	0	0	0	1	0	0	0	0	0
0	0	0	1	0	0	0	0	0	0	1	0	0
0	0	0	0	0	0	0	0	0	0	0	0	0
0	0	0	0	1	0	0	1	0	0	0	1	0
0	1	0	0	0	0	0	1	0	0	0	0	0
0	1	0	0	1	0	0	1	0	0	1	1	0
0	0	0	1	0	0	0	1	0	1	0	0	0
0	0	0	1	0	0	1	1	0	0	0	1	0
0	1	0	0	1	0	0	1	0	0	0	0	0
1	0	0	0	0	0	0	1	0	0	0	0	0
0	0	0	0	0	0	0	0	0	0	0	0	0
1	0	0	1	0	0	0	1	0	0	0	0	0
0	0	0	0	0	0	0	1	0	0	0	0	0
0	0	0	1	0	0	0	1	0	0	0	0	0
0	1	0	0	0	1	0	1	0	1	1	0	0
1	0	0	0	1	0	0	1	0	0	0	0	0
0	0	0	0	0	0	0	0	0	0	0	0	0
0	0	0	1	0	0	0	1	0	0	0	1	0
1	0	0	1	0	1	0	1	0	1	0	0	0

0	0	0	1	0	0	0	1	0	0	0	0	0
0	0	0	0	0	0	0	0	0	0	0	0	0
0	0	0	0	0	0	0	0	0	0	1	0	0
1	1	0	1	0	1	0	0	0	0	0	0	0
0	1	0	1	0	1	0	1	0	0	0	0	0
0	0	0	1	0	0	0	1	0	0	0	0	1
0	0	0	0	0	0	0	0	0	0	0	0	0
0	0	0	1	1	0	0	1	0	0	1	1	0
0	1	0	1	1	0	0	1	0	0	0	0	0
0	0	0	0	0	0	0	1	0	1	0	0	0
1	1	0	1	1	0	0	1	0	0	0	0	0
0	0	0	0	0	0	0	0	0	1	0	0	0
1	0	0	0	0	0	0	0	0	0	0	0	0
0	0	0	0	0	0	0	1	0	0	0	0	0
0	0	0	1	0	0	0	1	0	0	0	0	0
0	0	0	0	0	0	0	1	0	0	0	0	0
1	0	0	0	0	0	0	1	0	0	0	0	0
0	0	0	0	0	0	0	0	0	0	0	0	0
0	0	0	0	1	0	0	1	0	0	0	1	0
0	0	0	0	1	0	0	0	0	0	0	0	0
0	0	0	0	0	0	0	0	0	0	1	0	0
0	0	0	0	1	0	0	0	0	0	0	0	0
1	0	0	0	0	0	0	0	0	1	0	0	0
0	0	0	1	0	0	0	0	0	0	1	1	0
1	1	0	0	1	0	0	1	0	0	0	0	0
0	1	0	0	0	0	0	1	0	0	0	0	0
0	0	0	0	0	0	0	0	0	0	0	0	1
0	0	0	0	0	0	1	1	0	0	0	0	0
0	0	0	0	0	0	0	0	0	0	0	0	0
0	0	0	0	0	0	0	0	0	0	0	0	0
0	0	0	0	0	0	0	0	0	0	0	0	0
0	0	0	1	0	0	0	0	0	0	0	0	0
1	0	0	1	0	0	0	1	0	1	0	0	0
0	0	0	0	0	0	0	0	0	0	0	0	0
1	0	0	0	0	0	0	0	0	0	1	0	0
0	1	0	1	1	0	0	0	0	0	0	0	0
0	0	0	0	0	0	0	1	0	0	0	0	0
0	0	0	0	0	0	0	1	0	0	0	0	0
1	1	0	0	0	0	0	1	0	0	0	0	0
0	0	0	1	0	0	0	1	0	0	0	0	0
0	0	0	0	0	0	0	1	0	0	0	0	0
0	0	0	0	1	0	0	1	0	0	1	0	0
1	0	0	0	1	0	0	1	0	0	0	0	0
0	1	0	0	0	0	0	0	1	0	1	1	0
0	0	0	0	0	0	0	0	0	0	1	1	0
0	0	0	0	0	0	0	0	0	0	0	0	0
1	0	0	0	0	0	0	1	0	0	0	0	0
0	0	0	1	0	0	0	0	0	0	0	0	0
0	0	1	1	0	0	0	0	0	0	0	0	1
1	0	0	0	1	0	0	1	0	0	1	1	0
0	0	0	0	0	0	0	1	0	0	0	0	0

0	0	0	0	1	0	0	1	0	0	1	0	0
1	0	0	0	0	0	0	1	0	1	1	1	0
0	0	0	1	0	0	0	0	0	0	0	0	0
0	0	0	0	0	0	0	0	0	0	1	0	0
1	0	0	0	0	0	0	1	1	1	0	0	0
0	0	0	1	1	0	0	1	0	0	0	0	0
0	0	0	0	0	0	1	0	0	0	0	0	0
0	0	0	0	0	0	0	0	0	0	1	1	0
0	0	0	1	0	0	0	0	0	0	0	0	0
0	1	0	0	0	1	0	1	0	0	0	0	0
0	0	0	0	0	1	0	1	0	0	0	0	0
0	1	0	0	1	0	0	1	0	0	0	0	0
0	0	0	0	0	1	0	1	0	1	0	0	0
0	0	0	1	0	0	0	1	0	0	0	0	0
1	0	0	0	0	0	0	0	0	1	0	0	0
1	1	0	0	0	0	0	1	0	0	0	0	0
0	0	0	1	0	0	0	1	0	1	0	0	0
0	0	0	0	1	0	0	1	0	0	0	0	0
0	0	0	1	0	1	0	1	1	0	0	0	0
0	0	0	0	0	0	0	0	0	0	1	0	0
0	1	0	0	0	0	0	1	0	0	1	0	0
1	0	0	0	0	1	0	0	0	0	0	0	0
0	0	0	0	0	0	0	0	0	1	1	0	0
0	0	0	0	0	0	0	1	0	0	0	0	0
0	1	0	0	1	0	0	1	1	0	0	1	1
0	1	0	0	1	0	0	1	0	0	0	1	0
0	0	0	0	0	0	0	0	0	0	0	0	1
0	0	1	0	0	0	0	0	0	0	0	0	0
0	0	0	1	0	0	0	1	0	0	0	0	0
0	0	0	1	0	0	0	1	0	0	1	0	0
0	0	0	0	0	0	0	0	0	0	1	0	0
0	1	0	0	1	0	0	1	0	0	0	1	0
1	0	0	0	0	0	0	1	0	0	0	0	0
0	0	0	0	1	1	0	1	0	0	0	0	0
0	0	0	0	0	1	0	1	0	0	1	0	1
0	0	0	0	1	0	0	0	0	0	0	0	0
0	0	0	0	0	0	0	1	1	0	0	0	0
0	0	0	1	1	0	0	0	0	0	0	0	0
1	0	0	0	0	0	1	1	0	0	1	0	1
0	0	0	0	0	0	0	1	0	0	1	1	0
0	0	0	0	0	1	0	1	0	0	0	0	0
0	0	0	0	0	0	0	0	0	0	0	0	1
0	0	0	0	0	0	0	0	0	1	0	0	0
1	0	0	1	0	0	0	0	1	0	0	0	0
0	1	0	0	1	1	0	1	0	1	0	0	0
0	1	0	0	1	0	0	1	0	0	0	0	0
0	0	0	1	0	0	0	1	0	0	1	0	0
0	0	0	0	0	0	0	1	0	0	1	0	0
1	0	0	0	0	0	0	0	0	0	0	0	0
0	0	0	0	0	0	0	1	0	0	0	0	0
1	0	0	0	0	1	0	1	0	0	0	0	0

0	1	0	0	0	0	0	0	0	0	0	0	0
0	0	0	1	1	0	0	1	0	0	0	0	0
0	0	0	0	0	1	0	1	0	0	0	0	0
0	0	0	0	0	0	0	1	0	0	0	0	0
0	0	0	0	0	0	0	1	0	0	0	0	0
0	0	0	0	0	0	0	0	0	0	1	0	0
0	0	0	0	1	0	0	0	0	0	0	0	0
0	0	0	0	0	0	0	1	0	0	1	0	0
0	1	0	0	1	0	0	1	0	0	0	0	0
0	0	0	0	0	0	0	0	0	0	1	0	0
0	1	0	1	0	1	0	0	0	0	0	0	0
1	0	0	0	0	0	0	1	0	0	0	0	0
0	1	0	0	0	0	0	0	0	0	0	0	0
1	0	0	0	1	1	0	0	0	0	1	0	0
0	1	0	0	1	1	0	1	0	0	0	0	0
0	0	0	0	0	0	0	1	0	0	0	0	0
0	0	0	0	0	0	0	0	0	0	0	1	0
0	1	0	0	1	0	0	0	0	0	1	1	1
1	0	0	0	1	0	0	0	0	0	0	0	0
0	1	0	0	0	0	0	0	0	0	0	0	0
1	1	0	0	0	1	0	0	0	0	1	1	0
1	0	0	0	0	0	0	0	0	0	0	0	0
0	0	1	1	0	0	0	0	0	0	0	0	0
1	0	0	0	0	0	0	1	0	0	1	0	0
1	0	0	0	1	0	0	1	0	0	0	0	0
1	0	0	0	0	1	0	0	0	0	0	0	0
1	0	0	0	0	0	0	1	0	0	0	0	0
1	0	0	0	1	0	0	1	0	0	0	0	1
0	0	0	1	0	0	0	1	0	1	1	0	0
1	0	0	0	0	0	0	1	1	1	1	0	0
0	0	0	1	0	0	0	1	1	0	0	0	0
0	1	0	0	0	0	0	1	0	0	0	0	1
0	0	0	0	1	0	0	1	0	0	0	0	0
0	0	0	1	1	0	0	1	0	0	1	1	1
0	0	0	1	1	0	0	0	0	0	0	0	0
1	0	0	0	0	0	0	0	0	0	1	0	0
0	1	0	0	1	0	0	0	0	0	0	0	0
0	0	0	0	0	0	0	1	0	0	0	0	0
0	0	0	1	0	0	0	1	0	0	0	0	0
1	1	0	0	0	0	0	0	0	0	1	1	0
0	0	0	1	0	0	0	0	0	0	0	0	0
1	0	0	0	1	0	0	0	0	0	1	0	0
0	0	0	0	0	0	0	0	0	0	0	0	0
1	0	0	1	0	0	0	1	0	0	1	0	0
1	0	0	0	0	0	0	0	0	0	0	0	0
0	1	0	0	0	1	0	0	0	0	0	0	0
0	0	0	0	0	0	0	0	0	0	0	0	0
1	0	0	0	0	0	0	1	0	0	1	0	0
0	0	0	0	0	0	0	1	0	0	0	0	0
1	0	0	1	0	0	0	1	0	0	0	0	0
0	0	0	0	0	0	0	0	0	0	0	0	0

0	0	0	1	0	0	0	1	0	0	0	0	0
1	0	0	0	0	0	0	0	0	0	1	0	0
0	0	0	1	0	0	0	0	0	1	0	0	0
1	0	0	1	0	0	1	0	0	0	0	0	0
0	1	0	0	0	0	0	0	0	0	0	0	0
0	0	0	0	0	0	0	1	0	0	0	0	0
0	0	0	0	0	0	0	1	0	0	0	0	0
1	0	0	0	0	1	0	1	0	0	1	1	0
0	0	0	0	0	0	0	0	0	0	0	0	0
0	0	0	1	0	0	0	0	0	0	0	0	0
1	0	0	0	0	0	0	0	0	0	0	0	0
0	0	0	0	0	0	0	1	0	1	0	0	0
0	0	0	1	1	0	0	1	0	0	1	1	0
0	0	0	1	1	0	0	1	0	0	1	1	0
0	0	0	1	1	0	0	0	0	1	0	0	0
0	0	0	0	0	0	0	1	0	0	0	0	0
0	0	0	1	0	0	0	1	0	0	0	0	1
1	0	0	1	0	0	0	1	0	0	0	1	0
0	0	0	0	0	0	0	1	0	0	0	0	0
0	0	0	1	0	0	0	1	0	0	0	1	0
0	1	1	0	0	0	0	0	0	0	0	0	0
0	1	0	0	0	1	0	1	0	0	0	0	0
0	0	0	0	0	1	0	1	0	0	0	0	0
0	1	0	0	1	0	0	0	0	0	0	0	0
0	0	0	1	0	1	1	0	1	0	0	0	0
0	1	0	0	1	0	0	1	0	0	0	0	0
1	1	0	0	0	1	0	0	0	0	0	0	0
0	0	0	0	0	1	0	0	0	0	0	0	0
0	1	0	0	1	1	1	0	0	0	0	0	0
0	0	0	0	0	1	0	0	1	0	0	0	0
0	1	0	0	1	1	0	0	0	0	0	0	0
0	0	0	0	1	0	0	0	0	0	1	0	0
0	0	0	0	0	0	0	0	0	0	0	0	0
0	1	0	0	0	0	0	1	0	0	0	0	0
0	0	0	0	1	0	0	0	0	0	0	0	0
0	0	0	0	0	0	0	0	0	0	0	0	0
0	1	0	0	1	0	0	1	0	0	0	0	0
0	0	0	1	0	0	0	0	0	0	0	0	0
1	1	0	0	0	0	0	0	0	0	0	0	0
0	1	0	0	0	0	0	0	0	0	0	0	0
0	0	0	0	0	0	0	1	0	0	0	0	0
0	1	0	1	0	0	0	0	0	0	0	0	0
0	0	0	0	0	0	0	0	0	0	0	0	0
0	1	0	0	0	1	0	0	0	0	0	0	0
0	1	0	0	0	1	0	1	0	0	0	0	0
0	0	0	0	0	0	1	1	0	0	1	0	0
0	1	0	0	0	1	0	0	0	0	0	0	0
1	1	0	0	0	0	0	1	0	0	0	0	0
0	1	0	0	0	0	0	1	0	0	0	0	0
0	1	0	0	1	0	1	0	0	0	0	0	0
0	1	0	0	1	0	0	1	0	0	0	0	0

0	0	1	0	0	0	0	1	0	0	1	0	0
1	1	0	0	1	0	0	0	0	0	0	0	0
1	1	0	0	1	0	0	0	0	0	1	1	0
0	1	0	0	0	0	1	0	0	0	0	0	0
0	1	0	0	0	0	0	0	0	0	1	0	0
0	1	0	0	0	0	0	0	0	0	0	0	0
0	1	0	0	1	0	1	0	0	0	1	0	1
0	0	0	1	0	0	0	0	0	0	1	0	0
0	0	0	1	0	0	0	0	0	0	1	0	0
1	1	0	0	1	0	0	0	0	0	0	0	0
0	0	0	0	1	0	0	0	0	0	0	0	0
0	0	0	0	0	0	0	0	0	0	0	0	0
0	0	0	1	0	0	0	0	0	0	0	0	0
1	1	0	0	0	0	0	0	0	0	0	0	1
0	1	0	0	0	0	0	0	0	0	0	0	0
0	0	0	0	0	0	1	0	0	0	0	0	0
0	0	0	0	0	0	0	0	0	0	0	0	0
0	0	0	0	1	0	0	0	0	0	0	0	0
0	1	0	0	1	0	0	0	0	0	0	0	0
0	0	0	1	1	0	0	0	0	0	0	0	0
0	0	0	0	1	0	0	0	0	0	0	0	0
0	0	0	0	0	0	0	0	0	0	0	0	0
1	0	0	0	1	0	0	0	0	0	0	0	0
0	1	0	0	1	0	0	0	0	0	0	0	0
1	0	0	0	1	1	0	0	0	0	0	0	0
0	0	0	0	0	0	0	0	0	0	0	0	0
0	0	0	0	1	0	0	0	0	0	0	0	0
0	0	0	1	0	0	0	0	0	0	0	0	0
0	0	0	1	0	0	0	1	0	0	0	0	0
0	0	0	0	0	0	0	0	0	0	1	0	0
0	0	0	0	0	0	0	0	0	0	0	0	0
1	0	0	1	0	0	0	0	0	0	0	0	0
0	0	0	1	0	0	0	0	0	0	0	0	0
1	0	0	0	0	0	0	0	0	0	1	0	0
0	0	0	1	0	0	0	0	0	0	0	0	0
0	0	0	0	1	0	0	0	0	0	1	0	0
0	0	0	1	0	0	0	0	0	0	0	0	0
0	0	0	1	0	0	0	0	0	0	0	0	0

SIRS	MODS	Shock	Respir	Pneumo	electr	Hypopr	Acute	Acute	Acute	Gastro	Coagul	Heart
0	1	0	0	0	0	0	0	0	0	0	0	0
0	1	0	0	0	0	0	0	0	0	0	0	0
1	0	1	0	1	1	0	0	0	0	0	0	0
0	1	0	0	0	0	0	0	0	0	0	0	0
0	0	0	0	0	0	0	0	0	0	1	0	0
1	0	0	0	0	0	0	0	0	0	0	0	0
0	0	0	0	0	0	0	0	0	0	0	0	0
0	1	0	0	0	0	0	0	0	0	0	0	0
0	1	0	0	1	0	0	0	0	0	0	0	0
1	0	0	0	1	0	0	0	0	0	0	0	0
0	1	0	0	0	0	0	0	0	0	0	0	1
0	1	0	0	0	0	0	0	0	0	0	0	0
0	1	0	0	1	0	0	0	0	0	0	0	0
0	1	0	0	0	1	1	0	0	0	0	0	0
0	1	0	0	1	0	0	0	0	0	0	0	0
0	1	0	1	1	0	1	0	0	0	0	0	1
1	0	0	0	0	0	0	0	0	0	0	0	0
0	0	0	0	0	0	0	0	0	0	0	0	0
0	0	0	0	0	0	0	0	0	0	0	0	0
0	0	0	0	0	0	0	0	0	0	0	0	0
0	1	0	0	1	0	0	0	0	0	0	0	0
0	0	0	0	0	0	0	0	0	0	0	0	0
0	1	1	0	0	0	0	0	0	0	0	0	0
0	0	0	0	1	0	1	0	0	0	0	0	0
0	1	0	0	0	0	0	0	0	0	0	0	0
0	1	0	0	0	0	0	0	0	0	0	0	0
0	0	0	0	0	0	0	0	0	0	0	0	0
0	1	0	0	1	0	1	0	0	0	0	0	0
0	0	0	0	0	0	0	0	0	0	0	0	0
1	0	0	0	0	1	0	0	0	0	0	0	0
0	0	0	0	0	1	0	0	0	0	0	0	0
1	1	0	0	0	0	0	0	0	0	0	0	0
0	1	0	0	1	0	0	0	0	0	0	0	0
0	1	0	1	0	1	1	0	0	0	0	0	0
0	0	0	0	0	0	0	0	0	0	0	0	0
1	0	0	0	0	0	0	0	0	0	0	0	0
0	0	0	0	0	1	0	0	0	0	0	0	0
0	0	0	0	0	0	0	0	0	0	0	0	0
1	0	0	1	0	0	1	0	0	0	0	0	0
1	1	0	0	0	0	0	0	0	0	0	0	0
0	0	0	0	0	0	0	0	0	0	0	0	0
0	0	0	0	0	0	0	0	0	0	0	0	0
0	0	0	0	0	0	0	0	0	0	0	0	0
1	1	0	0	0	0	0	0	0	0	0	0	0
0	1	1	0	0	0	0	0	0	0	0	0	0
0	0	0	0	0	0	0	0	0	0	0	0	0
0	1	0	0	0	0	0	0	0	0	0	0	0
0	0	0	0	1	0	0	0	0	0	0	0	0
0	0	0	0	1	0	0	0	0	0	0	0	0
0	0	0	0	1	0	0	0	0	0	0	0	0

0	1	0	0	0	0	0	0	0	0	0	0	0
0	0	0	0	1	0	0	0	0	0	0	0	0
0	1	1	0	0	0	0	0	0	0	0	0	0
0	0	0	0	0	0	0	0	0	0	0	0	0
1	1	0	0	0	0	0	0	0	0	0	0	0
0	0	0	0	1	0	0	0	0	0	0	0	1
0	0	0	0	0	0	0	0	0	0	0	0	0
0	0	0	0	0	0	0	0	0	0	0	0	0
0	0	0	0	0	0	0	0	0	0	0	0	0
0	0	0	0	0	0	0	0	0	0	0	0	0
0	0	0	0	0	0	0	0	0	0	0	0	1
0	0	0	0	1	0	0	0	0	0	0	0	0
0	0	0	0	0	0	0	0	0	0	0	0	0
0	1	0	0	0	0	0	0	0	0	0	0	0
0	1	0	0	0	0	0	0	0	0	0	0	0
0	1	0	0	1	0	0	0	0	0	0	0	0
0	0	0	0	0	0	0	0	0	0	0	0	0
0	0	0	0	0	0	0	0	0	0	0	0	0
0	1	0	1	0	0	0	0	0	0	0	0	0
0	0	0	0	0	0	0	0	0	0	0	0	0
0	1	0	0	0	0	0	0	0	0	0	0	0
0	0	0	0	0	0	0	0	0	0	0	0	0
0	0	0	0	0	0	0	0	0	0	0	0	0
0	0	0	0	1	0	0	0	0	0	0	0	0
0	0	0	0	0	0	0	0	0	0	0	0	0
0	0	0	0	0	0	0	0	0	0	0	0	0
1	1	0	0	0	0	0	0	0	0	0	0	0
0	1	0	0	0	0	0	0	0	0	0	0	0
0	0	0	0	0	0	0	0	0	0	0	0	0
0	0	0	0	0	0	0	0	0	0	0	0	0
0	1	1	0	1	0	1	0	0	0	1	0	0
0	1	0	0	1	0	0	0	0	0	1	0	1
0	1	1	0	0	0	0	0	0	0	0	0	0
1	1	0	0	0	0	0	0	0	0	0	0	0
0	1	0	0	0	0	1	0	0	0	0	0	0
0	1	0	0	1	0	0	0	0	0	0	0	0
0	1	0	0	1	0	0	0	0	0	0	0	0
0	0	0	0	0	1	0	0	0	0	0	0	0
0	1	0	0	0	0	1	0	0	0	1	0	0
0	1	1	0	0	0	0	0	0	0	0	0	0
0	0	0	0	0	0	0	0	0	0	0	0	0
0	1	0	0	1	0	1	0	0	0	0	0	0
1	1	0	0	0	0	0	0	0	0	0	0	0
0	0	0	0	0	0	0	0	0	0	0	0	0
0	1	0	0	0	0	0	0	0	0	0	0	0
0	1	0	0	0	0	0	0	0	0	0	0	0
0	1	0	1	0	0	0	0	0	0	1	0	1
0	0	0	0	0	0	0	0	0	0	0	0	0
0	1	0	0	1	0	0	0	0	0	0	0	0
0	1	0	0	1	0	0	0	0	0	0	0	0
0	0	0	0	0	0	0	0	0	0	0	0	0

0	0	0	0	0	0	0	0	0	0	0	0	0
0	1	0	0	0	0	0	0	0	0	0	0	1
0	0	0	0	1	0	0	0	0	0	0	0	1
0	0	0	0	1	0	0	0	0	0	0	0	0
0	0	0	0	0	0	0	0	0	0	0	0	0
0	0	0	0	0	0	0	0	0	0	0	0	0
0	1	1	0	0	0	0	0	0	0	0	0	0
0	0	0	0	0	0	0	0	0	0	0	0	0
0	0	0	0	1	0	0	0	0	0	0	0	0
0	0	0	0	1	0	0	0	0	0	0	0	0
0	0	0	0	0	0	0	0	0	0	0	0	0
0	1	0	0	1	0	0	0	0	0	0	0	0
0	0	0	0	0	0	0	0	0	0	0	0	0
0	0	1	0	0	0	0	0	0	0	0	0	0
0	1	1	0	1	0	0	0	0	0	0	0	0
0	0	0	0	0	0	0	0	0	0	0	0	0
0	0	0	0	1	0	0	0	0	0	0	0	0
0	0	0	0	0	0	0	0	0	0	0	0	0
1	0	0	0	0	0	0	0	0	0	0	0	0
0	0	0	0	1	0	0	0	0	0	0	0	0
0	0	0	0	1	0	0	0	0	0	0	0	0
0	0	0	1	1	1	0	0	0	0	0	0	1
0	1	0	0	0	1	0	0	0	0	0	0	1
0	0	0	1	1	1	1	0	0	0	0	0	0
0	0	0	0	0	0	0	0	0	0	0	0	0
0	0	0	0	1	0	0	0	0	0	0	0	0
0	0	0	0	0	0	0	0	0	0	0	0	0
0	0	0	0	0	0	0	0	0	0	0	0	0
0	0	0	0	0	0	0	0	0	0	0	0	0
0	1	0	0	1	0	0	0	0	0	0	0	1
0	0	0	0	1	0	0	0	0	0	0	0	0
0	0	0	0	0	0	0	0	0	0	0	0	0
0	0	0	0	0	0	0	0	0	0	0	0	0
0	0	0	0	0	0	0	0	0	0	0	0	0
0	0	0	0	0	0	0	0	0	0	0	0	0
0	0	0	0	0	0	0	0	0	0	0	0	0
0	0	0	0	0	0	0	0	0	0	0	0	0
0	0	0	0	0	0	0	0	0	0	0	0	0
0	0	0	0	0	0	0	0	0	0	0	0	0
0	0	0	0	0	0	0	0	0	0	0	0	0
0	0	0	0	1	0	0	0	0	0	0	0	1
0	0	0	0	1	0	0	0	0	0	0	0	0
0	0	0	0	1	0	0	0	0	0	0	0	0
0	0	0	0	0	0	0	0	0	0	1	0	0
0	0	0	0	0	0	0	0	0	0	0	0	0
0	0	0	0	0	0	0	0	0	0	0	0	0
0	0	1	1	1	0	0	0	1	0	0	0	1
0	1	0	0	0	0	0	0	0	0	0	0	0
0	0	0	0	0	0	0	0	0	0	0	0	0
0	0	0	0	0	0	0	0	0	0	0	0	0
0	0	0	0	0	0	0	0	0	0	0	0	0

0	0	0	0	0	0	0	0	0	0	0	0	0
0	0	0	0	1	0	0	0	0	0	0	0	0
0	1	0	0	0	0	0	0	0	0	0	0	0
0	1	0	1	1	0	0	0	0	0	1	0	1
0	0	1	1	1	0	0	0	0	0	0	0	0
0	0	0	0	0	0	0	0	0	0	0	0	0
0	1	0	1	1	0	0	0	0	0	1	0	0
0	1	0	1	1	0	0	0	0	0	0	0	0
0	0	0	0	0	0	0	0	0	0	0	0	0
0	0	0	0	0	0	0	0	0	0	0	0	0
0	0	0	0	1	0	0	0	0	0	0	0	1
0	0	0	0	1	0	0	0	0	0	0	0	0
0	0	0	0	0	0	0	0	0	0	0	0	0
0	0	0	0	0	0	0	0	0	0	0	0	0
0	0	0	0	0	0	0	0	0	0	0	0	0
0	0	0	0	0	0	0	0	0	0	0	0	0
0	0	0	0	0	0	0	0	0	0	0	0	0
0	0	0	0	0	0	0	0	0	0	0	0	0
0	0	0	0	0	0	0	0	0	0	0	0	0
0	0	0	0	1	0	0	0	0	0	0	0	0
0	0	0	1	1	0	0	0	0	0	0	0	0
0	1	1	0	0	1	1	0	0	0	0	0	0
0	0	0	0	0	0	0	0	0	0	0	0	0
0	0	0	0	0	0	0	0	0	0	0	0	0
0	0	0	0	0	0	0	0	0	0	0	0	0
0	1	0	0	1	0	0	0	0	0	1	0	0
0	1	1	0	0	0	1	0	0	0	0	0	1
0	0	0	0	0	0	0	0	0	0	0	0	0
0	0	0	0	0	0	0	0	0	0	0	0	0
0	0	1	0	1	0	0	0	0	0	0	0	1
0	0	0	0	0	0	0	0	0	0	0	0	0
0	0	0	0	0	0	0	0	0	0	0	0	0
0	0	0	0	0	0	0	0	0	0	0	0	0
0	1	1	0	0	0	1	0	0	0	0	0	1
0	0	0	0	1	0	0	0	0	0	0	0	0
0	0	0	0	0	0	0	0	0	0	0	0	0
0	1	0	0	0	0	0	0	0	0	0	0	0
0	0	0	0	0	0	0	0	0	0	0	0	1
0	1	0	0	1	0	0	0	0	0	0	0	1
0	0	0	0	1	0	0	0	0	0	0	0	0
0	0	0	0	0	0	0	0	0	0	0	0	0
0	0	0	0	0	0	0	0	0	0	0	0	0
0	0	0	0	0	0	0	0	0	0	0	0	0
0	0	0	0	0	0	0	0	0	0	0	0	0
0	0	0	0	0	0	0	0	0	0	0	0	0
0	0	0	0	1	0	0	0	0	0	0	0	0
0	1	1	0	1	0	1	0	0	0	0	0	1
0	0	0	0	1	0	0	0	0	0	0	0	0
0	1	1	0	0	1	0	0	0	0	0	0	0
0	0	0	0	0	0	0	0	0	0	0	0	0
0	1	0	0	1	0	0	0	0	0	0	0	1

0	0	0	0	0	0	0	0	0	0	0	0	0
0	0	0	0	1	0	0	0	0	0	0	0	1
0	1	0	1	1	0	0	0	0	0	0	0	1
0	0	0	0	0	0	0	0	0	0	0	0	0
0	1	0	0	1	0	0	0	0	0	0	0	1
0	0	0	0	0	0	0	0	0	0	0	0	1
0	0	0	0	1	0	0	0	0	0	0	0	0
0	0	0	0	0	0	0	0	0	0	0	0	1
0	0	0	0	1	0	0	0	0	0	0	0	0
0	0	0	0	0	0	0	0	0	0	0	0	0
0	1	1	0	1	0	1	0	1	0	0	0	0
0	0	0	0	0	0	0	0	0	0	0	0	0
0	0	0	0	0	0	0	0	0	0	0	0	0
0	0	0	0	0	0	0	0	0	0	0	0	0
0	0	0	0	0	0	0	0	0	0	0	0	0
0	0	0	0	0	0	0	0	0	0	0	0	0
0	0	0	0	0	0	0	0	0	0	0	0	0
0	0	0	0	0	0	0	0	0	0	0	0	0
0	1	0	0	0	0	1	0	0	0	0	0	0
0	0	0	0	0	0	0	0	0	0	0	0	0
0	0	0	0	1	0	0	0	0	0	0	0	0
0	1	1	0	0	0	0	0	0	0	0	0	0
0	0	0	0	1	0	0	0	0	0	0	0	1
0	0	0	0	0	0	0	0	0	0	0	0	0
0	1	1	1	1	1	0	0	0	0	0	0	0
0	0	0	0	0	0	0	0	0	0	0	0	0
0	1	1	0	0	0	0	0	0	0	0	0	0
0	0	0	0	1	0	0	0	0	0	0	0	0
0	1	0	0	0	1	0	0	0	0	0	1	1
0	1	1	0	1	0	0	0	0	0	0	0	0
0	0	0	0	0	0	0	0	0	0	0	0	0
0	0	0	0	0	0	0	0	0	0	0	0	0
0	1	0	0	0	0	0	0	0	0	0	0	0
0	0	0	0	1	0	0	0	0	0	0	0	0
0	1	1	0	1	1	0	0	0	0	1	0	1
0	0	0	0	0	0	0	0	0	0	0	0	0
0	0	0	0	0	0	0	0	0	0	0	0	0
0	0	0	0	0	1	0	0	0	0	0	0	0
0	1	0	0	1	0	1	0	0	0	1	0	0
0	0	0	0	1	0	0	0	0	0	0	0	0
0	0	0	0	0	0	0	0	0	0	0	0	0
0	1	1	0	1	0	1	0	0	0	0	0	0
0	1	0	0	1	0	0	0	0	0	0	0	0
0	0	0	0	0	0	0	0	0	0	0	0	0
0	0	0	0	0	0	0	0	0	0	0	0	1
0	1	0	1	1	0	0	0	0	0	0	0	1
0	1	0	0	0	1	1	0	0	0	0	0	0
0	1	0	0	0	0	0	0	0	0	0	0	0
0	1	1	0	0	1	1	0	0	0	1	0	0
0	0	0	0	1	0	0	0	0	0	0	0	0
0	0	0	0	0	0	0	0	0	0	0	0	0

0	1	0	0	0	1	0	0	0	0	0	0	1
0	1	0	0	0	0	0	0	0	0	0	0	1
0	1	1	1	1	1	1	0	0	0	0	0	0
0	1	0	0	1	1	1	0	0	0	0	0	0
0	0	0	0	1	0	0	0	0	0	0	0	0
0	0	0	0	0	0	0	0	0	0	0	0	0
0	0	0	0	0	0	0	0	0	0	0	0	0
0	0	0	0	0	0	0	0	0	0	0	0	0
0	0	0	0	1	0	0	0	0	0	0	0	0
0	1	0	0	0	1	1	0	0	0	0	0	0
0	0	0	0	0	0	0	0	0	0	0	0	0
0	1	0	0	1	1	1	0	0	0	0	1	0
0	0	0	0	0	1	0	0	0	0	0	0	1
0	0	0	0	1	0	0	0	0	0	0	0	0
0	1	0	1	0	0	0	0	0	0	0	0	0
0	1	0	1	1	0	0	0	0	0	0	0	1
0	0	0	0	0	0	0	0	0	0	0	0	0
0	0	0	0	0	0	0	0	0	0	1	0	0
0	1	0	0	0	0	0	0	0	0	0	0	1
0	1	0	0	1	1	0	0	0	0	1	0	1
0	1	0	0	0	0	0	0	0	0	0	0	0
0	1	0	0	0	0	0	0	0	0	0	0	0
0	1	0	0	0	0	0	0	0	0	0	0	1
0	0	0	0	0	0	0	0	0	0	0	0	0
0	0	1	0	0	1	0	1	0	0	1	1	0
0	0	0	0	0	0	0	0	0	0	0	0	1
0	0	0	0	1	0	1	1	0	0	0	0	1
0	1	1	0	0	0	1	1	1	0	0	1	0
0	0	0	0	0	0	0	0	0	0	0	0	0
0	0	0	0	0	0	0	0	0	0	0	0	0
0	0	0	0	0	0	0	0	0	0	0	0	0
0	1	1	1	0	0	1	0	1	0	1	0	1
0	1	1	1	1	1	1	0	0	0	0	0	1
0	1	0	0	1	0	0	0	0	0	0	0	1
0	0	0	1	1	1	0	1	1	0	0	1	1
0	0	0	0	0	0	0	0	0	0	0	0	0
0	0	0	0	0	0	0	0	0	0	0	0	0
0	0	0	0	0	0	0	0	0	0	0	0	0
0	0	0	0	1	0	0	0	0	0	0	0	0
0	1	0	1	1	1	1	0	0	0	1	0	0
0	1	0	0	0	0	0	0	0	0	0	0	0
0	0	0	0	0	1	0	0	0	0	0	0	0
0	0	0	0	1	0	0	0	0	0	0	0	0
0	1	1	1	0	0	1	0	1	0	0	1	1
0	0	0	0	1	0	0	0	0	0	0	0	0
0	0	0	0	0	0	0	0	0	0	0	0	0
0	1	1	0	1	0	0	0	0	0	0	0	1
0	1	0	0	1	0	0	0	0	0	0	0	1
0	0	0	0	1	0	0	0	0	0	0	0	0
0	1	0	1	1	1	1	0	0	0	0	0	1
0	1	0	0	0	0	0	0	0	0	0	0	0

0	0	0	0	0	1	0	0	0	0	0	0	0
0	0	0	0	0	0	0	0	0	0	0	0	0
0	0	0	0	0	0	0	0	0	0	0	0	0
0	1	0	0	1	0	1	0	0	0	0	0	1
0	1	0	0	1	0	1	0	0	0	0	0	0
0	0	0	0	1	0	0	0	0	0	0	0	0
0	0	0	0	0	0	1	0	0	0	0	0	0
0	1	0	0	0	0	0	0	0	0	0	0	1
0	1	1	1	1	0	1	0	0	0	0	1	1
0	0	0	0	0	0	0	0	0	0	0	0	1
0	0	0	0	0	0	0	0	0	0	0	0	0
0	0	0	0	0	0	0	0	0	0	0	0	0
0	1	0	0	1	1	0	0	0	0	0	0	1
0	0	0	0	0	0	0	0	0	0	0	0	0
0	0	0	0	0	0	0	0	0	0	0	0	0
0	0	0	0	0	0	0	0	0	0	0	0	1
0	1	0	0	0	0	1	0	0	0	0	0	0
0	0	0	0	0	0	0	0	0	0	0	0	0
0	1	0	0	0	0	1	0	0	0	0	0	1
0	0	0	0	0	0	0	1	0	0	0	0	0
0	0	0	1	1	0	0	0	0	0	0	1	0
0	0	0	0	0	0	0	0	0	0	0	0	0
0	0	0	1	0	0	0	0	0	0	0	1	0
0	0	0	0	1	0	0	0	0	0	1	0	0
0	1	1	0	0	0	0	0	0	0	1	1	0
0	0	0	1	1	0	0	0	0	0	0	1	0
0	0	0	0	0	0	0	0	0	0	0	0	0
0	0	0	0	1	0	0	0	0	0	0	0	0
0	1	1	1	1	0	0	0	0	0	0	0	0
0	1	0	0	1	0	0	0	0	0	0	0	0
0	0	0	0	0	0	0	0	0	0	0	0	0
0	1	1	0	0	0	0	0	0	0	0	0	0
0	1	1	0	1	1	1	0	0	0	0	0	1
0	1	0	1	1	1	1	1	1	0	1	0	0
0	0	0	0	0	1	0	1	0	0	0	0	0
0	0	0	0	0	1	0	0	0	0	0	0	0
0	0	0	0	0	0	0	0	0	0	0	0	0
0	0	0	0	0	1	1	1	0	0	0	0	0
0	1	1	0	1	0	0	0	0	0	0	0	1
0	1	0	0	1	1	0	0	0	0	1	1	1
0	0	0	0	0	0	0	1	0	0	0	0	0
0	1	1	1	1	0	1	1	1	0	0	1	1
0	0	0	0	0	1	0	1	0	0	0	0	0
0	0	0	0	1	1	0	0	1	1	1	0	0
0	0	0	0	1	0	0	1	0	0	0	0	0
0	0	0	0	0	0	0	1	0	0	0	0	0
0	0	0	0	1	0	0	0	0	0	0	0	0
0	0	1	0	0	0	0	1	1	0	0	0	1
0	0	0	0	0	1	0	1	0	1	0	0	0
0	0	0	0	1	0	1	0	0	0	1	0	1
0	0	1	1	0	1	0	1	0	0	0	0	0

0	0	0	1	1	0	0	1	1	0	0	0	0
0	1	0	0	0	1	0	0	0	0	0	0	0
0	0	1	0	1	0	0	0	0	0	1	1	0
0	0	1	1	0	0	0	1	1	0	1	1	0
0	1	0	0	1	0	0	0	0	0	0	0	0
0	0	0	0	0	0	0	1	0	1	0	0	1
0	1	1	0	0	1	1	0	1	0	0	1	0
0	0	0	0	0	0	0	1	0	0	0	0	0
0	0	0	0	0	1	0	1	0	1	0	0	0
0	0	0	0	0	1	0	0	0	0	0	0	0
0	0	0	0	0	1	1	1	0	0	1	0	0
0	0	0	0	0	0	0	1	0	0	0	0	0
0	0	0	0	0	0	0	1	0	0	0	0	0
0	0	0	0	1	0	0	1	1	0	1	1	0
0	0	0	0	1	0	0	1	1	0	0	0	0
0	0	0	0	1	1	0	1	0	0	0	0	0
0	0	0	0	0	1	0	1	0	0	0	0	0
0	1	0	1	1	0	1	1	1	0	1	1	1
0	0	0	0	0	1	0	0	0	0	0	0	0
0	0	0	0	0	1	0	0	0	0	0	0	0
0	0	0	0	1	0	0	0	0	0	0	0	0
0	0	0	0	0	1	0	1	0	0	0	0	0
0	0	0	0	1	0	0	1	1	0	1	0	0
0	0	0	0	0	1	0	0	0	0	0	0	1
0	0	0	0	0	1	0	1	0	0	0	0	0
0	0	0	0	0	1	1	1	0	0	0	0	0
0	0	0	0	0	1	0	1	0	0	0	0	0
0	0	0	0	0	0	0	0	0	0	0	0	0
0	0	0	0	0	0	0	0	0	0	0	0	0
0	0	0	0	0	1	0	1	0	0	0	0	0
0	1	1	1	1	0	0	0	0	0	1	0	0
0	0	0	0	0	1	0	1	1	0	0	0	0
0	0	0	0	0	1	0	1	0	0	0	0	0
0	0	0	0	0	1	0	1	0	0	0	0	0
0	0	0	0	1	0	0	1	0	0	0	0	0
0	0	0	0	1	0	0	1	0	0	0	0	0
0	0	0	0	0	0	0	0	0	0	0	0	0
0	0	0	0	0	0	0	0	0	0	0	0	0

Viral load	Leukoc	Neutro	lympho	erythr	Hemogl	Platel	ALT (U	AST (U	CK (U/
1.22*10^6	2.5	2.1	0.3	4.22	125	80	71	185	474
3.14*10^3	0.8	0.6	0.2	4.26	144	45	66	107	805
1.71*10^5	4.9	4.2	0.5	4.23	134	36	128	328	509
1.43×10^5	2.2	1	0.9	5.46	173	33	118	358	2624
5.21*10^3	4.3	1.8	0.8	4	124	56	263	580	1022
1.37*10^4	7.4	6.5	0.6	5.49	176	51	114	141	154
2.1*10^2	1.8	0.4	1	3.59	112	23	192	234	320
1.76*10^3	8.7	4.9	2.3	4.28	122	34	161	408	581
5.96*10^4	11.4	4.7	0.7	2.5	71	55	165	968	308
1.05*10^5	1.7	1.2	0.5	4.36	139	24	229	577	1498
1.6*10^5	2.3	1.7	0.5	5.27	161	15	87	304	2952
4.8*10^3	19.2	12	6.9	2.79	85	27	92	205	677
1.5*10^4	4.1	2.7	0.9	4.16	131	38	97	276	1384
3.8*10^4	4.2	4.5	1.6	3.91	117	58	147	268	235
3.5*10^4	5.8	5.2	0.4	5.15	156	21	86	296	3123
4.9*10^4	14.1	12.6	0.8	4.49	140	33	100	214	965
5.0*10^2	2.1	0.3	1.3	4.35	125	21	161	495	1971
1.63*10^2	6.1	2.9	2.4	3.66	111	36	88	57	29
3.5*10^2	9.8	9	0.5	4.13	137	13	71	123	230
7.2*10^2	1.9	0.8	0.9	4.38	138	60	136	149	165
7.0*10^2	3.6	3.2	0.3	3.02	98	50	139	156	893
8.2*10^3	1	7.1	1.1	4.26	131	42	149	69	355
1.2*10^6	1.8	0.9	0.7	4.39	129	27	126	370	1825
2.4*10^4	1.3	0.6	0.6	4.27	137	44	100	97	376.6
4.8*10^4	1.7	1.3	0.4	4.06	120	64	79	142	249
6.1*10^5	4.4	3.2	1.1	4.32	133	30	140	416	2297
1.8*10^3	1.2	0.6	0.6	4.39	142	51	64	122	1135
7.7*10^4	2	1.2	0.8	4.68	138	15	136	413	6707
4.2*10^2	1.5	0.7	0.8	4.86	154	56	69	65	785
3.3*10^4	2.5	2	0.4	4.82	144	81	175	273	399
4.85*10^2	1.4	0.5	1.2	4.39	119	54	99	160	707.9
1.08*10^5	2.6	1.8	0.7	4.88	140	39	398	702	680
4.6*10^3	2.5	2.27	0.23	4	114	39	46	78	118
2.58*10^3	4.7	4.2	0.43	4.2	129	53	56	123	435
9.44*10^3	6.3	2.07	3.22	4.6	140	31	137	337	217
1.5*10^2	1.2	0.54	0.59	4.1	121	55	107	225	599
7.1*10^4	2.4	2.25	0.72	4.2	125	36	26	31	370.7
1.1*10^2	2	1.17	0.71	5.2	153	55	38	61	232
6.1*10^2	2.6	1.27	1.11	4.3	128	35	114	179	570
2.1*10^5	1.1	0.48	0.58	4.8	136	22	81	350	1246
1.5*10^3	1.03	0.76	0.24	3.85	113	58	46	141	1630
1.0*10^3	1.69	1.19	0.33	5.18	161	56	47	74	2019
1.5*10^4	1.95	0.8	0.98	4.4	134	23	107	159	1493
1.18*10^3	5.17	4.34	0.8	3.93	115	28	90	301	2139
1.1*10^7	1.12	0.79	0.03	4.98	153	62	265	335	318
1.6*10^3	6.48	5.73	0.68	4.95	143	30	301	386	706
3.8*10^5	3.11	1.76	1.22	3.45	101	72	330	1109	886
9.0*10^3	2.61	1.85	0.64	4.44	136	62	57	228	1168
1.5*10^2	5.46	4.35	0.88	4.09	116	33	34	88	246
6.04*10^2	1.37	0.83	0.47	4.57	132	53	46	88	445

5.83*10^4	1.17	0.77	0.34	4.4	140	50	295	858	2825
1.83*10^2	1.27	0.8	0.34	5.11	158	50	56	98	304
8.6*10^4	1.5	0.91	0.52	4.21	131	30	216	679	1749
2.1*10^2	5.46	2.02	2.56	4.12	107	56	112	205	721
9.0*10^3	4.23	3.63	0.51	4.35	131	80	40	119	370
5.3*10^2	1.46	0.93	0.48	4.87	148	30	60	149	1022
1.5*10^2	4.29	1.23	2.08	4.92	136	38	135	366	765
9.0*10^3	7.04	5.98	0.88	4.62	139	27	80	201	2225
3.1*10^3	2.96	2.32	0.52	4.14	136	83	134	135	400
8.7*10^2	0.95	0.7	0.21	4.33	128	77	19	36	282
1.3*10^4	0.72	0.42	0.25	4.78	142	34	108	81	190
4.6*10^2	1.24	0.68	0.46	4.64	136	48	56	158	428.9
2.3*10^2	10.07	8.86	0.99	4.79	136	44	91	227	3167
1.0*10^3	4.29	3.79	0.44	4.64	140	85	102	303	570.1
1.8*10^6	5.01	3.13	1.53	4.67	134	54	157	900	2213
4.7*10^6	8.21	4.61	2.47	3.88	119	59	125	990	3348
3.9*10^3	6.51	5.18	1.05	4.14	130	80	63	105	231
1.3*10^3	1.78	1.38	0.38	4.64	133	78	47	66	96
3.2*10^5	11.96	8.36	2.57	5.49	158	37	552	890	743
2.9*10^2	1.98	1.26	0.64	4.25	126	68	30	52	74
4.9*10^5	1.12	0.81	0.29	4.18	124	38	72	165	249
7.0*10^2	3.2	2.53	0.5	3.22	104	54	62	102	132
1.9*10^4	1.06	0.67	0.31	5.02	163	26	133	284	590
1.0*10^4	1.72	1.28	0.38	4.47	138	60	60	118	822
6.2*10^3	1.38	0.6	0.65	4.37	130	92	47	65	160
3.0*10^3	2.55	2.07	0.38	4.3	92	61	49	110	181
4.8*10^2	4.76	1.21	2.81	4.87	148	84	123	110	440
1.2*10^3	1.62	6.7	1.3	4.71	147	42	147	352	1797
3.6*10^2	9.42	7.22	1.51	5.71	176	31	221	329	329
1.7*10^2	1.58	1.18	0.33	4.84	148	60	50	154	1032
1.5*10^3	1.03	0.69	0.26	4.83	160	27	39	171	2019
9.9*10^2	4.21	3.3	0.47	2.02	66	16	38	82	976
5.3*10^3	1.55	1.14	0.34	4.61	139	43	38	83	343
5.8*10^3	2.08	1.64	0.36	4.33	129	82	45	78	86
4.6*10^3	4.41	1.51	2.03	4.95	140	30	152	325	195
2.7*10^2	1.8	1.35	0.34	4.78	146	92	32	28	124
6.0*10^3	12.95	11.61	0.99	5.15	152	57	233	495	2988
2.4*10^2	1.53	0.63	0.71	4.18	122	68	184	374	700
3.9*10^4	3.04	2.53	0.4	5.65	165	56	277	1165	2817
1.2*10^6	9.46	6.71	1.26	5.29	153	60	203	1244	367
2.2*10^2	7.5	6.72	0.39	4.37	130	35	28	32	47
5.6*10^2	2	2.16	0.52	4.35	129	53	49	70	301
8.2*10^2	1.72	1.35	0.27	3.42	113	14	76	296	2274
6.5*10^2	4.72	5.82	1.57	4.91	143	75	89	115	709
3.7*10^5	5.14	2.05	0.72	3.72	106	92	524	497	2433
1.4*10^5	1.91	1.15	0.62	4.93	146	35	152	336	412
3.6*10^5	6.09	2.16	1.89	4.1	119	70	131	408	362
7.2*10^3	3.32	1.35	1.38	4.28	125	46	132	577	2863
1.3*10^3	1.09	0.76	0.26	3.44	109	50	74	196	2847
6.4*10^4	11.3	3.73	1.23	4.44	130	78	105	155	324
9.4*10^2	5.65	4.97	0.52	4.89	160	35	142	335	369

1.2*10^4	15.05	5.08	0.67	4.59	142	53	59.7	61.5	47
3.1*10^6	1.79	1.35	0.35	5.4	166	33	176.9	349	1228
3.4*10^5	1.29	0.79	0.37	4.06	115	45	45.3	108.1	1707
1.8*10^5	2.62	1.62	0.63	5.58	178	26	34.7	19.2	65
1.3*10^4	11.74	1.89	5.56	4.48	131	98	108.9	137.7	877
5.4*10^3	1.03	6.67	1.98	3.85	117	35	105.9	426.6	911
1.7*10^2	1.1	1.17	0.45	4.11	122	31	45.9	123.6	217
1.0*10^3	2.24	1.07	0.87	5	150	75	36.7	112.8	2473
7.3*10^3	4.48	0.74	0.47	4.38	141	30	133.5	275.5	1640
7.4*10^3	4.66	3.8	1.51	4.37	136	50	46.8	159	1767
5.2*10^3	2.85	5.32	1.44	3.87	130	40	42.8	79.4	206
3.1*10^2	3.85	6.22	1.1	4.86	140	38	326.8	51.8	145
6.5*10^2	2.76	1.57	0.82	4.21	123	23	492.5	1008	145
1.6*10^3	3.87	2.92	0.9	3.69	113	52	63.2	169.5	422
2.98*10^4	12.46	1.02	0.25	4.25	121	92	121.7	206	2666
1.03*10^2	2.02	0.91	0.99	3.8	117	64	16.4	50.9	734
1.76*10^2	5.53	3.63	1.19	4.36	137	53	32.6	71	220
6.69*10^4	3.82	2.59	0.9	5.8	167	31	135.4	262.1	240
2.4*10^6	7.05	5.66	0.72	3.57	76	60	168.4	640.2	854
3.3*10^6	7.73	1.45	0.94	4.39	131	20	67	507.7	1246
7.9*10^3	1.04	1.77	0.42	4.74	141	30	150.6	444.6	809
4.8*10^5	15.01	2.2	1.69	5.06	150	63	73	295.7	2545
4.1*10^5	6.61	1.37	0.77	4.83	146	54	74.4	533.4	3827
7.0*10^3	7.29	4.96	0.27	3.8	113	51	47.8	210.2	1285
2.1*10^3	2.64	1.37	0.68	5.01	158	27	227.1	280	364
6.1*10^4	1.97	1.39	0.46	4.84	156	45	115.5	424.5	3579
2.7*10^3	2.53	3.74	1.52	4.9	151	39	25.5	44.9	672
1.1*10^2	1.69	0.48	1	4.75	153	78	25.9	50.9	562
3.7*10^2	3.23	0.54	2.3	4.08	129	81	39.2	54.9	218
7.9*10^4	1.93	1.01	0.78	5.89	183.1	40	41.2	180.2	1984
2.01*10^3	3.02	1.66	1.15	5.1	156	30	130	296.6	1710
8.35*10^2	3.26	2.58	0.62	4.01	135	83	21.3	68.5	214
5.75*10^3	2.77	2.35	0.37	4.05	102	70	28	52.8	91
9.88*10^2	1.59	0.81	0.63	4.61	133	65	11.5	34.7	75
8.5*10^3	1.93	1.27	0.55	4.57	135	98	29.3	34.3	43
2.5*10^4	3.8	2.75	0.13	4.82	138	40	56	110	459.3
2.2*10^3	1.92	0.95	0.51	6.12	177	49	17.2	41.6	100
7.6*10^5	6.18	3.67	1.62	4.44	124	43	65.2	218.6	450
2.0*10^3	1.55	1.03	0.4	4.76	155	62	38.7	49.1	762
1.6*10^5	4.04	3.18	0.53	4.25	130	52	108.7	306	2072
4.7*10^3	1.78	1.13	0.59	4.45	140	72	74.4	232.9	1754
5.1*10^4	4.38	2.93	0.82	4.16	123	21	72.9	115.9	357
8.2*10^3	1.55	0.88	0.52	5.62	165	65	297.8	515.8	2578
2.7*10^2	2.11	1.64	0.41	4.18	145	41	19.7	48	844
1.2*10^2	1.15	0.81	0.22	4.09	127	76	15.6	22.5	80
3.7*10^4	1.28	0.93	0.29	3.96	121	63	30.8	42.5	327
1.8*10^2	7.16	4.85	1.69	4.66	144	41	59	166.1	587
8.5*10^2	2.76	0.89	0.25	4.88	151	52	330.9	1212	1646
1.2*10^2	7.44	6.28	0.52	4.22	122	88	38.6	27.3	28
3.3*10^2	5.33	4.53	0.63	3.9	118	77	41.9	91.3	315
9.2*10^3	1.42	1.13	0.16	3.75	116	15	64.7	38.4	95

1.9*10ˆ3	1.52	1.03	0.42	5.21	161	74	138.3	251.8	258
7.4*10ˆ3	6.96	7.82	0.98	4.91	142	34	369	273.2	347
1.4*10ˆ5	2.64	0.97	1.16	4.18	127	20	122.4	324	245
1.6*10ˆ4	0.82	0.52	0.25	4.93	149	13	72.3	272	3727
9.3*10ˆ3	19.46	3.81	1.5	4.54	142	80	32.8	27.5	135
5.4*10ˆ4	0.58	0.15	0.38	4.47	136	64	69.2	296	2363
1.1*10ˆ3	1.38	1	0.3	4.3	120	99	18.9	35.3	657
6.6*10ˆ4	19.17	5.28	0.34	5.62	162	38	103.4	358.7	5634
7.9*10ˆ1	4.4	1.72	1.64	5.05	155	33	36.2	133	282.7
1.8*10ˆ2	3.13	4.23	0.37	4.4	136	22	37.3	73.7	71
1.4*10ˆ3	3.76	6.82	0.32	4.68	142	54	339.8	506.1	2155
3.1*10ˆ4	0.94	0.66	0.23	4.19	129	72	47.7	120.3	264
1.1*10ˆ3	2.13	1.61	0.41	3.95	122	55	31.2	90.5	97
1.6*10ˆ4	6.95	1.43	0.45	4.44	131	52	30.1	25.5	60
4.3*10ˆ4	1.61	0.94	0.61	4.28	125	68	95.1	211.3	1318
4.0*10ˆ3	3.12	4.09	1.47	4.32	136	86	134.7	398.7	1620
3.3*10ˆ3	2.8	1.62	0.97	5.33	158	75	64.1	156.5	565
8.9*10ˆ4	4.02	2.38	0.9	5.01	136	33	188.2	440.2	2168
2.2*10ˆ2	6.05	4.51	1.2	4.46	138	75	35.2	73	574
8.2*10ˆ2	2.1	1.22	0.8	3.68	109	70	18.9	43.4	578
8.9*10ˆ2	1.48	1	0.41	3.8	115	80	26.2	46.1	513
1.0*10ˆ2	1.29	0.82	0.41	4.52	130	53	25.3	46.4	126
2.0*10ˆ1	5.84	4.39	0.96	4.95	157	35	38.3	141.4	4497
1.0*10ˆ4	4.45	1.64	2.57	2.8	83	38	225.6	853	4500
2.5*10ˆ2	1.62	1.25	0.26	4.63	144	82	155	325	587
4.3*10ˆ4	0.63	0.29	0.29	4.64	136	24	72.2	234.2	3260
3.0*10ˆ3	3.03	1.96	0.83	5.47	153	63	63.7	203.2	1388
1.5*10ˆ7	9.42	8.75	0.52	4.63	128	40	59.7	131.7	323
1.5*10ˆ7	4.28	3.37	0.61	5.43	167	35	91.8	213	724
7.6*10ˆ3	4.81	3.99	0.71	4.35	129	69	106	220.7	595
>10ˆ8	5.01	2.11	2.21	4.72	145	25	123.7	757.7	2084
3.1*10ˆ6	3.71	1.97	1.42	4.65	134	23	113.7	488.8	904
2.3*10ˆ3	1.01	0.47	0.39	3.65	109	79	21.7	59.1	209
5.0*10ˆ5	4.4	4.05	0.3	4.21	126	83	37.8	72.5	97
1.0*10ˆ4	1.49	1.06	0.37	4.39	134	95	31.8	45	192.8
1.2*10ˆ5	0.87	0.54	0.29	4.32	129	97	39.4	64.6	197
1.6*10ˆ2	2.01	0.97	0.97	4.43	133	68	54.5	92.2	395
2.3*10ˆ2	4.12	3.13	0.9	4.12	120	60	27.2	45.1	82
1.5*10ˆ7	12.31	10.8	0.63	4.46	136	62	176.2	1075	1154
1.7*10ˆ2	3.76	3.14	0.57	4.54	137	76	25.7	44.5	174
5*10ˆ4	2.35	1.73	0.48	5.16	160	30	75.3	372.6	5793
1.2*10ˆ4	1.98	1.22	0.61	5.35	172	58	122.9	552	7670
2.6*10ˆ4	8.13	6.48	0.98	4.47	128	69	86.1	139.4	308
1.4*10ˆ3	7.6	6.05	1.21	4.4	137	63	55.5	118.7	1395
1.1*10ˆ3	1.47	1.01	0.36	4.45	136	79	45.3	50.2	226
2.6*10ˆ2	4.15	3.26	0.75	4.24	127	95	38.8	87.1	516
3.3*10ˆ3	2.13	1.57	0.5	4.5	129	58	47.9	86.4	198
1.3*10ˆ4	1.69	1.32	0.26	3.96	119	31	61.2	316.2	6664
8.9*10ˆ3	1.37	1	0.29	4.41	132	57	21.9	49.6	254
2.1*10ˆ3	0.62	0.33	0.25	4.4	125	77	34.7	66.8	141
3.4*10ˆ3	1.5	0.88	0.35	5.19	158	42	54.6	98.5	534

1.1*10^4	4.07	3.34	0.65	4.19	134	49	53.9	140.9	223
1.4*10^4	6.7	5.71	0.67	4.16	109	26	65.5	175.5	234
3.8*10^2	5.9	4.96	0.78	4.08	130	69	24.5	46.6	226
1.2*10^4	7.62	6.89	0.57	5.41	168	43	33.3	211.7	1886
7.37*10^2	1.26	0.87	0.33	4.21	147	38	28.6	67.5	723
2.22*10^4	2.65	2.12	0.48	3.93	86	63	38.3	130.9	437
1.78*10^4	2.22	1.18	0.97	4.44	127	94	120.7	232.8	606
6.6*10^5	2.99	2.1	0.78	4.54	153	23	108.4	260.8	452
3.3*10^4	2.69	1.91	0.64	3.62	110	47	48.1	87.7	169
4.7*10^4	7.2	6.49	0.46	3.72	122	38	211	375.5	383
7.9*10^3	6.21	5.25	0.78	4.13	129	67	46.9	105.3	1352
8.9*10^3	1.6	1.13	0.35	4.79	137	63	109.5	206.9	116
3.1*10^4	1.76	1.44	0.28	4.61	149	20	52.1	287	1993
2.6*10^3	2.39	1.22	1.59	4.28	119	70	66.4	91.7	590
5.0*10^4	1.84	1.08	0.72	3.77	114	32	76.8	292.7	7006
4.1*10^4	5.93	2.29	2.64	3.79	110	33	52.9	105.1	453
1.8*10^2	0.86	7.97	0.59	4.71	149	51	28	48.1	271
1.2*10^4	7.87	2.08	1.41	5.54	159	63	309.3	428.3	902
1.7*10^4	1.48	1.07	0.45	5.13	160	78	64.7	212.4	444
1.7*10^2	0.86	3.38	0.61	4.1	126	87	24.6	37.1	157
2.2*10^5	1.9	2.54	0.24	4.82	140	69	48	147.6	284
3.3*10^3	2.61	1.34	0.75	5.06	149	64	110.3	80.9	62
1.1*10^3	1.61	0.68	0.39	4.28	127	62	57	146.5	853
1.7*10^5	2.79	2.62	1.32	4.85	149	31	159.3	478.3	1103
4.2*10^3	7.05	2.68	0.34	4.22	140	68	22.8	41.2	384
1.4*10^3	9.71	1.72	1.7	4.46	137	80	46.8	77.3	83
6.35*10^6	1.75	0.64	0.98	4.16	141	13	158.8	545.7	1778
2.0*10^4	2.29	1.41	0.78	4.59	137	57	52.2	151.9	859
9.5*10^3	1.73	1.03	0.63	4.64	135	54	26.9	85.1	1346
4.1*10^2	1	0.75	0.24	3.67	115	51	17.1	36.7	56
6.3*10^6	16.45	7.96	5.2	4.29	143	74	331.7	1407	2315
5.8*10^4	0.84	0.52	0.26	5.26	153	22	53.6	170.2	389
5.3*10^2	1.19	0.44	0.71	3.47	110	79	18.3	39.3	127
1.7*10^3	0.6	0.39	0.17	4.12	137	38	73.4	187.2	649
8.5*10^4	3.87	2.62	1.06	3.71	114	24	99.7	405.2	4932
1.1*10^3	4.87	4.22	0.52	4.7	145	60	115.5	213	1047
1.5*10^3	4.61	3.28	1.13	5.23	148	28	144.2	491.7	1150
1.7*10^4	2.56	0.63	1.19	3.59	120	35	50.4	191.3	901
3.9*10^4	2.8	1.4	1.24	5.11	160	34	42.1	134	1455
2.1*10^3	7.65	6.3	1.04	4.07	124	49	43.2	155.5	878
6.7*10^6	2.5	1.36	0.42	5	155	39	623	592	1057
1.4*10^4	3.12	2.55	0.45	3.82	120	67	439	1676	1182
1.2*10^2	3.44	1.64	1.2	5.06	156	76	274.9	330.3	1817
2.5*10^4	2.04	1.01	0.95	4.69	145	42	113.9	219.8	1761
2.5*10^4	2.02	1.42	0.57	3.35	103	31	30.5	83.5	229
1.3*10^2	1	0.33	0.5	4.13	118	72	39.8	101.5	251
1.0*10^2	4.11	3.58	0.39	4.01	115	45	21.8	49.6	219
1.0*10^2	5.86	4.92	0.78	4.8	145	29	47.2	105.3	2410
6.2*10^2	2.3	1.71	0.4	4.48	145	45	69	88	188.5
1.0*10^3	3.35	2.67	0.58	3.19	101	47	20.6	58.6	226
1.0*10^2	2.2	1.23	0.71	4.63	132	42	40.9	48	182

1.7*10^5	3.13	2.68	0.29	4.61	127	24	97.8	352.2	3802
6.7*10^4	3.77	1.8	1.42	4.56	134	62	35.4	124.4	245
2.2*10^3	1.49	0.74	0.66	3.53	113	50	62.6	87.6	108
7.6*10^4	2.14	1.53	0.54	4.27	137	35	74	219.3	943
1.5*10^4	1.39	0.86	0.43	3.5	107	57	14.7	44.7	327
9.6*10^3	3.44	3.15	0.26	3.92	129	85	18.4	42.9	376
2.3*10^3	1.14	0.87	0.2	4.83	144	92	63.4	111.7	782
1.2*10^4	5.86	5.23	0.54	4.67	141	62	42.2	127.6	234
8.0*10^2	1.51	0.83	0.47	3.99	129	79	29.1	38.5	428
1.4*10^4	1.27	0.89	0.32	4.3	129	33	26.9	97.7	251
2.1*10^2	5.49	4.62	0.64	4.42	116	85	27	40.1	45
1.1*10^6	2.47	1.93	0.38	4.71	140	44	119.2	182.7	1980
1.7*10^2	3.26	1.27	1.59	4.39	135	53	32.8	38.2	122
3.3*10^5	1.64	1.17	0.36	4.08	102	47	100.1	383.7	5331
5.8*10^3	3.45	2.85	0.51	4.44	133	56	59.4	150.3	880
4.3*10^6	1.54	0.92	0.57	4.58	142	54	123.6	251.8	1385
6.2*10^2	2.34	1.75	0.36	4.78	150	39	52.8	135.5	648
7.2*10^4	3.55	2.7	0.71	3.84	123	54	71.8	296.6	4696
4.3*10^2	2.99	1.01	1.54	4.61	147	63	35	76.4	145
9.6*10^2	5.41	4.76	0.42	2.1	63	31	17.7	51	218
1.4*10^4	2.94	2.48	0.4	4.81	142	44	82.3	228.5	562
3.8*10^2	4.73	3.94	0.71	4.75	144	78	81.5	144.7	683
1.1*10^6	4.96	3.17	2.51	4.89	145	52	86.8	620	1880
2.7*10^3	1.34	1.02	0.22	4.95	149	51	48.8	62.4	225
6.0*10^3	2.69	5.64	1.37	3.24	103	80	253.5	556.1	185
4.3*10^3	2.41	1.45	0.85	3.78	121	80	80.4	144.6	35.5
1.2*10^3	1.19	0.6	0.55	4.42	139	57	112.1	281.5	399.7
6.7*10^3	2	1.6	0.32	4.84	156	36	100.3	273.7	2257
6.9*10^2	9.86	7.12	1.41	4.63	143	30	21	79.5	376.2
6.4*10^4	1.76	1.49	0.66	3.34	112	72	40.5	111.9	689.1
2.2*10^2	0.84	0.57	0.2	4.17	125	63	23.2	47.1	375.1
2.4*10^2	0.79	0.39	0.36	4.9	147	66	20.6	35.1	507.2
2.2*10^2	7.23	6.73	0.35	3.82	119	70	59.9	68.5	81.4
1.9*10^6	2.24	1.43	0.69	4.6	151	26	62.6	350.4	903.9
6.8*10^2	1.78	1.5	0.21	4.17	129	40	38.6	106.7	722.8
1.3*10^3	4.5	3.5	0.88	4.88	152	51	66.1	176	4280
7.1*10^2	3.14	2.51	0.58	4.14	143	57	28.8	80.7	969.8
1.2*10^2	1.31	0.63	0.59	4.93	143	63	125.4	195.3	445.7
1.0*10^3	2.02	1.3	0.62	4.45	148	57	101.3	197	159.1
1.2*10^2	1.42	1.02	0.36	4.42	141	56	29.7	44.5	376.5
2.1*10^2	4.41	3.98	0.34	4.07	120	45	29.2	89.9	933.8
6.4*10^2	4.3	3.98	0.25	3.97	131	50	22.2	46.2	437.1
2.9*10^3	4.97	4.26	0.52	3.57	103	90	30.2	43.7	123.9
4.0*10^2	0.89	0.17	0.64	4.11	129	72	25.3	58.5	197.4
1.8*10^3	2.57	1.98	0.52	4.31	127	67	59	149.8	916.5
1.3*10^6	1.27	1.05	0.19	4.9	147	21	169.3	430.5	679.3
6.4*10^1	4.32	2.53	1.05	4.7	156	52	71.1	149.9	2254
1.6*10^3	5.3	4.58	0.54	4.44	133	33	96.8	245.6	368.9
1.6*10^3	0.96	0.36	0.52	5.01	166	23	199.5	464	205.6
2.1*10^2	3.67	2.63	0.79	4.63	149	83	26.9	69.5	410.6
3.0*10^2	0.98	0.47	0.42	3.87	111	29	21.4	59.1	177

1.0*10^2	3.05	1.3	1.29	4.51	141	41	139.6	310.4	3267
3.5*10^2	4.76	4.37	0.3	4.57	142	90	33.8	53.9	236
2.0*10^2	7.79	5.93	1.59	4.08	128	65	28.6	66	1432
5.4*10^4	1.72	2.14	0.38	4.19	132	39	116.6	286.1	327.9
2.8*10^3	1.9	1.2	0.59	4.5	140	35	88.5	282.3	1036
1.1*10^3	4.59	3.51	0.75	4.55	138	57	21.3	59.9	166.9
2.4*10^4	5.52	4.21	1.04	4.81	143	44	426.8	1073	279.2
3.4*10^2	2.88	1.38	1.09	5.19	172	47	141.2	313.5	1314
1.9*10^4	0.88	0.6	0.27	4.46	143	39	43.2	97.6	796.4
1.4*10^4	2.92	2.15	0.67	5.04	153	67	95.5	231.8	2015
1.5*10^5	2.47	1.99	0.42	2.91	107	9	25.4	79.9	765.5
1.2*10^4	2.71	1.14	1.12	5.47	175	20	88.6	207.1	431.5
1.4*10^2	1.76	1.29	0.32	4.66	144	56	37.8	43.6	149.1
6.9*10^1	5.5	4.3	0.93	4.59	145	71	17.3	28	192.2
2.7*10^5	8.04	7.49	0.41	5.13	159	69	217.4	374	2000
7.1*10^3	2.33	1.17	0.97	4.26	131	62	148.2	245.6	791.2
7.4*10^4	3.69	1.03	2.12	5.02	148	68	53.6	171	112.6
1.2*10^3	3.59	1.64	1.45	4.94	153	34	84.1	169.5	1128
1.4*10^3	2.27	1.48	0.66	3.96	126	40	40.1	45.4	91.4
1.4*10^5	1.12	0.77	0.29	4.71	136	27	65.8	88.8	85.5
9.7*10^4	1.26	0.56	0.57	4.78	137	42	90.8	125.4	152.1
1.8*10^4	1.87	1.43	0.33	4.22	129	81	40.8	48.5	69
7.4*10^4	7.37	5.3	0.81	4.59	137	35	518.7	82.6	728.8
1.9*10^4	2.27	1.27	0.94	4.84	152	25	332.3	85.2	1599
1.3*10^5	4.99	4.05	0.8	4.27	132	36	57.9	20	144
2.0*10^5	3.16	1.47	0.86	4.91	143	53	608.1	133.8	264.3
3.7*10^4	2.07	1.53	0.5	4.29	128	53	124.4	73.3	59.7
7.6*10^4	1.51	1.18	0.31	4.81	134	16	241.4	57	1500
2.6*10^2	9.46	7.79	0.52	5.03	147	61	201.4	135.3	653
1.1*10^2	4.41	2.94	1.25	4.23	124	46	82.5	35.3	457.1
5.2*10^3	1.67	1.18	0.45	3.74	118	33	88.1	33.8	1925
1.9*10^7	5.05	2.66	1.14	6.13	187	66	657.6	1923	3289
7.0*10^3	5.7	4.47	0.75	4.37	133	48	54.5	186.4	5238
7.6*10^4	7.41	1.93	5.3	4.34	121	26	58.8	211.6	2301
8.2*10^5	3.57	2.38	1.01	5.5	159	33	622.5	68.6	1977
7.4*10^3	1.44	1.01	0.38	4	116	23	55.5	91.4	284.9
6.6*10^4	1.4	1	0.35	4.36	129	40	57.2	148.3	447.2
1.3*10^3	8.59	7.39	0.76	4.05	118	52	188.3	438.1	519.6
2.4*10^3	2.91	2.23	0.57	3.92	127	33	62.1	98.5	518.8
3.0*10^3	4.98	4.08	0.71	4.7	135	31	30.3	41.5	53.3
3.2*10^2	2.04	1.45	0.53	4.86	140	87	193.9	437.2	911
1.4*10^3	1.8	1.55	0.22	4.7	141	53	17.2	41.7	267.1
1.2*10^4	3.26	2.94	0.24	3.24	111	43	90.1	174	1142
4.6*10^5	2	1.63	0.35	4.21	131	27	56.7	167.8	683.7
6.9*10^2	6.2	5.32	0.77	4.23	128	50	82.7	134.3	189.4
1.6*10^3	3.55	2.85	0.56	4.14	127	78	27.9	58.2	448.1
3.1*10^4	4.37	3.92	0.39	4.22	134	22	46.6	194.8	1740
2.1*10^2	4.7	2.17	1.71	4.3	135	34	122.1	124.1	263.2
3.4*10^7	2.67	2.13	0.5	4.48	129	49	52	317.2	900.4
2.0*10^7	1.19	0.8	0.25	3.85	121	10	118.3	456.7	690.6
2.4*10^3	2.53	2.11	0.37	4.42	137	66	171.2	316.3	649.7

1.5*10^5	2.97	1.39	0.61	5.22	154	54	127.5	725.8	588.9
9.8*10^2	2.75	1.82	0.86	4.15	117	31	34.9	143.2	219.2
2.5*10^4	2.53	1.71	0.76	4.37	129	51	50.4	188	551.5
4.6*10^4	3.61	1.14	0.87	4.11	121	40	126.7	321	1020
1.1*10^2	1.94	1.61	0.28	3.85	115	30	42.1	123.4	130.1
2.4*10^6	7.32	2.58	2.2	3.94	110	22	116.4	468.7	4010
1.7*10^4	1.73	1.22	0.44	5.36	159	74	338.2	712.1	3776
1.4*10^5	2.51	2.17	0.29	4.84	108	47	72.4	111.2	1274
2.8*10^1	2.99	1.37	0.83	5.31	157	88	145.9	317.7	870.5
5.8*10^2	3.24	0.93	1.76	4.74	140	82	117.1	156.8	2688
4.3*10^2	2.05	1.14	0.7	4.59	145	44	53.3	68.9	154.3
1.3*10^4	1	0.69	0.26	3.91	126	47	58.8	70.3	350.9
2.9*10^2	1.58	1.33	0.22	3.46	110	30	46.8	73	277.9
2.3*10^2	5.74	5.33	0.35	4.37	136	73	63.4	154.7	207.2
4.9*10^2	1.19	0.81	0.34	4.33	127	31	25.8	47.8	82.3
1.3*10^3	5.02	4.19	0.6	4.22	126	44	43.2	70.6	396.3
7.5*10^1	1.52	0.84	0.57	4.18	127	80	559.9	719.7	354.8
6.0*10^5	2.04	1.26	0.72	4.26	124	90	26.6	41.2	226.3
7.5*10^2	4.17	2.32	1.2	4.58	128	44	163.3	421.5	791.1
2.0*10^2	1.57	0.75	0.63	4.1	128	34	28.8	55	176.6
4.3*10^2	4.83	3.45	0.76	3.17	82	66	51.7	86.2	344.8
6.3*10^5	2.14	1.45	0.57	5.38	155	56	56.9	88.8	197.6
3.7*10^2	1.81	1.05	0.66	4.48	125	52	67.1	117.1	224.6
1.6*10^2	11.01	8.99	1.37	4.75	143	77	89.2	138.9	571.2
2.7*10^5	5.45	3.68	1.36	4.87	154	62	164.6	443.6	2071
1.1*10^3	2.06	1.43	0.58	4.57	129	71	61.2	199.7	787.6
2.2*10^4	1.84	1.13	0.61	4.8	154	19	138.1	532.8	682.4
2.9*10^5	1.77	1.08	0.62	4.64	152	35	77.7	239	3054
2.4*10^6	7.83	6.13	1.52	4.42	131	48	40.9	259.8	371
3.6*10^3	0.49	0.24	0.21	3.93	123	33	86.3	194.3	55.4
4.3*10^2	1.95	1.39	0.49	3.79	120	62	35.7	46.1	303.8
7.3*10^2	1.9	1.22	0.63	4.62	140	58	39.1	80.6	111.8
1.3*10^6	1.45	0.83	0.56	3.77	113	30	26.2	23.3	845
1.8*10^4	2.23	1.25	0.87	5.76	163	56	75.2	182.5	294.8
1.1*10^4	2.09	1.33	0.66	4.91	147	67	46.6	28	126.2
1.2*10^1	3.94	1.12	1.95	4.44	141	34	287.9	317.4	3060
9.4*10^2	1.65	1.12	0.46	4.28	126	62	52.3	75.2	197.5
1.0*10^4	2.71	1.9	0.74	5.16	176	90	76	234.8	4358

CK-MB	LDH (U	α-HBD	K(mmol	Na(mmo	Cl(mmo	BUN (m	SCr (m	PT (s)	APTT(s	Fib(g/	TT(s)	D-dime
8	836	567	3.9	139	106	2.44	87	14.2	52.9	1.9	18.2	2.68
4	347	267	3.1	138	102	3.67	61	13	45.2	1.7	18	3.39
2	984	733	3.2	130	98	1.82	55	12.5	58.2	1.65	20.7	21.52
101	1217	1015	4.6	133	96	14.33	179	14.9	115.2	1.34	27	7.57
23	657	428	3.4	138	102	1.78	70	10.7	36.4	1.99	17.9	2.12
6	489	361	4.11	132	91	5.6	168	11	44.5	2.61	17.1	2.56
13	305	253	2.9	133	103	5	74	15.5	56.2	1.93	19.6	0.22
10	1312	1181	4.2	139	103	13	226	12.2	38.1	2.13	23.7	2.01
2	2116	1858	4.9	135	104	3.95	89	14	41.8	2.35	23.5	9.42
45	858	535	4.33	138	102	7.24	93	14.7	38.7	2.88	20.4	1.78
117	743	734	4.1	137	102	17.2	237	14.5	56.4	2.25	34.4	1.27
55	972	1055	2.9	150	113	24.5	127	15.6	50.5	1.36	21.9	0.98
92	679	707	3.8	133	104	1.54	66	12.3	59.3	2.07	21.9	1.47
58	743	706	3.5	131	98	4	87	12.1	44.6	2.21	19.6	2.44
133	658	703	4.4	142	107	10.6	115	12.3	44.4	2.38	17.8	1.99
85	933	645	3.6	142	108	5.4	54	14.9	91.3	1.78	25.6	6.53
68	2286	1602	2.8	136	100	2.9	56	11.7	63.7	2.28	22	2.22
19	376	302	2.9	150	118	3.2	64	14	37.2	2.14	18.2	8.89
35	528	380	4	132	98	4.6	58	13.8	55.1	1.26	19	7.07
20	315	223	3.7	142	107	7.7	72	13.4	55.6	1.45	20.2	6.93
42	422	284	4.3	145	112	29.8	608	13	47.9	1.86	20.9	1.3
27	342	265	3	129	94	1.8	51	14.4	44.8	1.95	17.8	3.6
2828	926	932	4	141	107	10.6	131	14.3	66.2	1.7	23.5	3.66
43	252	150	3.5	135	106	4.7	61	11.3	58.4	2.25	19	0.98
26	369	364	4.1	130	97	10.2	99	13.2	66.3	1.93	23	8.12
125	1428	959	4.4	135	104	6.5	84	15.4	55.4	1.1	22.5	9.55
46	240	160	3.9	130	97	8.1	98	13.1	50.9	2.11	18.6	16.26
190	533	220	3.8	135	105	8.3	73	15.8	51.4	3.27	22	4.46
36	406	319	3.9	134	101	3.8	69	11.9	41.5	2.56	18.4	1.3
44	226	180	3.4	127	92	6.6	68	14.2	52.3	1.96	21.9	8.78
34	772	313	3.5	133	105	8.8	124	12.7	47.4	3.33	18.2	1.38
75	256	238	4	129	99	4.9	73	15.9	57.4	1.56	20.6	15.15
13	293	179.2	3.5	139.6	103	3.8	66.5	16.8	55.5	1.56	20.4	8.92
40	678	296.7	3.4	143.1	110	1.6	46.4	14.8	51.6	2.36	19.5	5.73
24	963	605.7	3.4	131.2	100	2.5	57.5	13.3	40.4	3.18	20.5	0.98
47	286	335	3.2	138.7	105	1.6	40.3	14.5	48.5	2.76	21.1	4.09
22.4	214	192	3.7	127	97	3.8	71.7	15.1	59.4	3.49	19.4	4.18
28	231	188	3.7	138.8	102	4.5	69	12.6	39.8	2.96	17.5	6.22
38	452	285	3.2	132.4	98	1.8	66.9	13	45.3	2.18	13	2.09
55	235	204	4.7	131	98	5.9	162.1	14.2	61.6	2.38	25.2	47.95
111	519	343.3	3.5	120.4	92	4.9	93	14.4	60.9	1.54	22.2	9.86
97	423	289.1	3.7	139.3	103	6.6	77.1	13.6	41.6	2.3	19.3	2.29
46	514	333	4.4	137.6	104	3	48.4	12.1	37.1	2.92	18.7	1.12
107	364	369	3.8	132.3	98	5.07	70.2	13.1	45.1	1.84	22.3	2.04
104	1008	640	5	139.3	103	12.33	99.1	15.1	40.5	2.66	17.9	9.22
56	1023	604.8	3.8	128.7	94	4.9	50	12.5	49.3	2.31	19.9	0.85
158	2873	1519	4.4	145.7	105	2.6	59	21	86.1	0.68	30.6	16.35
88	413	176	4.2	141.5	103	1.7	40	13	65.6	1.99	22.3	3.8
345	640	425.5	4.2	144.6	108	10.6	86.8	12.3	43.5	2.62	18.7	1.35
32	522	341.5	3.5	138	105	3.1	69.1	11.9	35.9	2.9	18.5	1.01

123	1281	731.7	3.8	138.2	103	4.3	52	13.8	76.1	2.03	19.4	5.86
60	407	273.6	4	135	102	7.6	72	13.2	35.7	2.59	18.3	2.83
127	1659	915.2	4	144.7	106	17.5	158	15.2	69.2	2.01	20.7	17.36
26	552	448.3	4.3	134	104	4.2	72.9	12.2	51.6	1.64	20.6	2.34
46	333	168	4.2	128.9	96	5.6	60	12.9	40.6	2.9	19.5	0.94
49	801	502.6	3.4	140	106	5.2	85.6	11.7	36.4	2.2	21.1	8.1
77	1077	982	3.7	137.1	104	5.3	85	12.8	52.5	2.25	20.6	0.85
60	787	477	4.3	138.8	108	6	70	12.4	47.5	2.18	19.4	6.75
43	408	272.1	4.9	135.3	101	4.1	83	13	36.8	1.97	19.1	2.9
19	239	151.3	3.3	129	95	4.4	57	13.6	33.5	2.77	17.9	0.95
17	248	226.7	3.5	125.5	92	4.5	59	14.6	48.7	1.75	20.1	2.98
50.5	909.3	357	4	129.1	96	15.5	119.8	16.9	58.1	2.28	19.9	5.67
131	916	597.3	3.3	133.9	103	5.7	65	12	32.7	2.03	22.2	1.36
66.7	709	273.2	4	135.5	103	4.3	90	14.3	56.2	2.31	19.2	2.81
122.2	2843	1054	4.1	137	102	4.9	93	14.2	77.7	1.58	26	2.75
325	4563	2908	4.7	134	103	13.6	183.4	17.6	71.9	1.53	24.6	12.96
23	428	282	4	132.1	99	1.9	50	11.9	43.4	2.55	18.1	1.12
22	356	289.9	4	134.8	102	8.9	66	14.7	44.8	2.25	18.6	10.75
83	2382	1559	3.5	133.8	98	4.6	53.2	12.1	36.6	2.46	20.2	1.33
13	253	166.6	3.8	131.7	96	3.6	68.9	14.6	37.4	2.86	17	2.38
40	630	363.7	3.8	127.9	99	5.5	70	16.4	69.3	1.95	19.8	21.92
21	329	8.8	4.1	133	99	6.3	74.4	14	35.9	2.18	20.7	2.25
165	1141	779	4.9	125	94	3.7	35	14.2	52.2	2.2	18.9	8.57
89	450	274	4.1	131.8	99	11.2	111	13.1	48.2	2.25	21.5	4.59
34	361	256.6	3.6	138	103	5.6	63.3	12.7	37.7	2.46	19.5	0.85
19	355	272.4	4.1	130.5	99	6.7	71.4	13.3	47	1.7	20.1	3.05
37	388	166	3.8	136.1	104	4.2	48	11.4	34.7	2.86	18.7	2.35
85	1007	650.7	3.4	133	101	6.2	82	12.8	53.1	1.49	21.3	5.87
26	186	771.3	3.1	134.8	97	4.3	74.2	12.8	47.3	2.9	17.3	1.51
51	686	436.6	3.1	127.9	94	4	74	15.1	43.8	2.49	18.6	16.75
73.8	365.7	159.4	4.3	126.1	93	7.8	79	13.7	47.1	2.01	19.9	11.59
35	442	283.2	3.5	125.5	93	12.5	243	15.4	51.3	1.47	20.6	19.61
28	451	312.3	4.7	127.5	95	8.6	99	16	46.1	1.82	19.2	21.79
24	722	455.8	3.8	132.8	100	5.8	79.4	13.5	48.6	2.31	19.7	0.52
44	772	527.7	3.3	132	101	3.4	42	12.7	40	2.06	19.5	0.99
20	218	155.2	3.3	135.1	96	3	68	14.8	41.3	2.99	16.6	0.54
93	1147	868.1	3.5	135.6		2.8	87.8	12.8	39.3	2.43	19.1	4.54
69	631	366.6	4	137.1	105	3.1	56	11.8	52.2	1.73	21.7	2.63
194	1659	1042	4.3	129.1	95	15.3	229.3	13	55.8	2.37	19.8	7.79
86	1852	982.7	4.2	126	90	7	101.7	16.2	118	1.52	22.3	27.33
18	212	143.8	3.7	133.3	99	7.1	113.7	14.8	49.7	5.53	16.5	3.04
25	317	210.9	3.2	133.7	97	3.2	80.1	13.4	44.6	2.18	18.6	4.71
202	2286	631.7	2.8	136	105	3.8	83.6	15.4	63.9	1.95	19.7	59.44
46	722	478.4	3.5	129	96	3.3	71	12.2	38.8	1.95	20.4	1.01
86.7	1143	383.2	3.7	135.8	104	13.6	139.7	15	53.5	0.45	33.6	11.7
128	1487	766.1	4	121.7	90	12.67	107.7	14.5	58.1	1.58	21.6	30.57
93	1730	835.6	3.7	132.6	103	6.1	77	19.3	51.6	1.05	25.8	21.1
65	1628	768.9	4.7	138.8	106	7.7	111.7	11.5	33.5	2.28	20.8	1.9
90	728	366.4	3.6	133.2	101	10.1	103.7	12.6	68.4	2.49	20.6	1.65
35	644	456.5	3.9	136.5	102	6.28	88.5	12.3	34.8	4.01	18.3	1.24
41	968	658.7	4	133	100	4.9	63.3	12.1	40.5	2.13	20.7	9.09

8.2	319	288	3.86	131	93	3.3	55	26.2	52.2	3.68	15.7	0.51
67.2	1130	837	5.28	131	97	8	98	14	42.6	1.8	18.8	8.89
39.3	579	475	3.23	130	96	4.3	91	12.6	43.6	1.99	19.9	2.28
8.8	272	250	4.3	128	97	3.9	66.7	11.1	35.9	2.25	19.4	1.66
35.3	845	648	4.09	147	110	5	60	13.5	31.9	2.18	19.1	2.07
36.8	1134	813	3.82	126	92	3.9	73	12.8	47.1	1.57	20.1	3.76
24	623	437	3.4	137	100	3.2	63	14.1	51.8	1.65	21.1	15.17
48.3	597	464	3.66	129	95	3.8	68	12.7	37	2.59	16.6	1.97
48.2	1533	1022	4.15	138	103	2.8	46	12.6	57.6	1.64	22.1	3.07
46.6	955	610	3.41	127	98	1.8	42	11	36.8	2.01	19.7	1.64
22.2	347	222	3.8	138.5	103	8.3	77	24.2	35.5	2.9	18.1	1.11
12.6	252	210	4.33	133	97	3.1	49	11.1	41.4	2.34	20	1.28
22.7	766	445	4.17	134	100	1.5	40	12.3	44.3	1.64	21.5	0.97
18.1	641	410	3.81	123	94	4.2	58	11.8	43.3	1.77	19.9	5.66
62.6	476.3	181.1	3.7	126	90	5.8	48.7	28.6	113.1	0.55	33.8	7.16
13.6	504	406	3.62	140	103	6.2	75	12.7	41.7	2.01	18.8	2.87
15.3	304	291	4.1	140	101	3.1	59	10.9	50.7	2.18	19	0.82
26	924	687	4.35	129	94	4.5	71	11.9	36.4	2.37	21.5	2.51
61.7	853	650	3.9	137	105	6.6	101.7	13.1	69.6	1.67	22.6	16.58
40.4	1983	1464	4	133	99	5	62	13.1	77.3	1.8	21.4	3.51
34.1	953	673	4.24	133	100	4.3	63	12.9	47.6	1.68	19.5	4.82
64.1	1853	1446	4.14	134	100	6	80	12.6	47.7	2.13	20.6	5.56
117.1	1889	1271	2.8	134	97	13	194.6	15.4	53.1	1.72	21.2	14.11
32.2	1649	1255	3.16	141	102	2.2	54	13.3	47.1	1.5	23.7	3.52
26.7	940	776	4.01	130	94	7.1	68	11.5	28.2	2.15	17.6	1.83
98.6	1293	966	4.19	136	101	4.1	60	12.2	48.7	2.56	17.8	11.23
22.3	385	303	4.2	140	104	8.7	116	12.3	48.9	2.49	18.5	3.02
19.2	322	268	4.19	139	99	3.4	74	11.6	39.4	2.86	17.3	0.94
16.1	335	251	3.77	136	98	2.9	55	11.1	38.2	2.15	19.1	1.11
46	569	317	4.39	127	93	7.9	97	14.2	54.9	1.68	20.4	35.33
35.2	912	690	3.78	128	95	4.1	48	12.5	45.8	1.75	19.9	5.7
15	337	277	4.19	131	105	3.3	64	13.5	53.1	2.34	18.1	3.02
23.7	242	202	4.06	138	106	4.4	52	11.9	28.1	1.75	20.2	1.25
15.4	287	243	3.53	136	104	2.3	48	12.5	35.3	1.87	17.3	0.81
11.2	351	296	4.38	134	98	3.9	58	12.5	31.4	3.86	16.8	5.76
34.6	523.6	215.6	3.2	136	100	4.8	71.7	12.3	51.1	2.23	20.2	2.1
21.5	436	278	4.47	135	100	8.7	114	12.2	33.7	1.75	21.5	0.9
38.8	1739	950	3.08	132	99	3.5	61	11.1	35.5	2.39	20	2.48
28.2	322	238	3.43	137	95	5.5	66	13.1	39.4	2.49	17.9	3.32
51.3	1750	824	3.38	130	96	2.4	44	12.1	38.7	1.73	21.3	4.94
36.3	695	520	4.11	135	90	12.8	117	12	37	2.49	18.7	2.17
25.3	1298	927	3.4	144	100	3.2	57	12.3	46.6	2.25	19.1	3.52
56.1	786	405	4.83	131	96	15.5	131	13.1	40.7	2.23	18	3.73
19.9	156	155	3.53	143	108	2.6	54	12.4	47.6	1.99	19.5	13.04
11.4	178	121	3.5	140	101	2.1	55	13.8	48.4	2.7	17.8	0.68
14.4	241	206	3.85	141	106	5.1	37	12.1	45.4	1.82	21.1	5.49
55.3	1886	1003	4.09	136	102	5.7	69	12.3	38.6	2.7	21.4	4.8
102.1	2241	901	4.25	137	95	5.8	53	14.1	128.5	1.82	21.9	17.19
5.2	130	70	3.22	139	103	5.4	73	16.4	32.7	5.68	16.5	1.23
30.1	518	240	3.27	132	96	4.2	39	12.1	45.5	1.57	19.8	11.32
5.2	241	135	4.24	145	107	4.6	57	13	38.6	4.01	16.7	3.17

19.7	641	461	4.54	140	98	3	72	12.6	41	2.18	18.4	5.51
23.5	449	373	4.3	136	102	3.8	54.3	11.5	38.4	2.52	18.8	0.62
23.8	589	444	3.7	138	104	2.6	44	11.9	37.1	1.99	18.9	0.38
102.8	736.3	360.2	3.7	126	95	3.6	53.6	13.2	60.4	1.99	18	23.86
13.2	389	327	5.15	134	102	8.1	97	15.9	44.7	4.95	15.4	1.34
54.1	1425	1020	2.98	133	95	3.9	64	12.3	43.5	2.01	20.1	155.6
32.2	340	227	2.9	138	103	3.9	83.6	13.7	45.8	1.49	19.1	11.2
108.7	1545	996	3.91	144	101	4.9	73	14	38.6	1.6	24.2	0.83
25.3	764.4	663.3	3.3	137	100	7.6	71.9	11.9	22.3	2.34	18.5	1
27	290	204	3.41	134	99	6.8	63	12.3	39.5	1.99	18.8	3.42
84.1	1281	832	3.86	135	97	5.2	68	12.2	48.2	1.54	20.4	41.08
24.7	636	266	3.91	134	96	5	45	19.1	85.6	2.06	19.7	1.52
20.8	501	355	3.67	137	102	4.6	46	12.1	43	1.95	20.4	12.3
13.2	309	270	3.76	135	100	5.3	46	12.8	42.4	1.83	19.8	2.36
33.1	790	541	3.32	136	101	4.4	63	12.5	40.6	2.08	20.8	14.42
76.3	1074	714	4.23	139	101	3.9	56	11.7	27.5	1.75	18.3	7.03
83.9	712	574	4.25	137	97	3.1	61	11.8	46.2	2.43	18.7	20.52
54.4	895	614	4.16	134	96	5.7	56	12	45	1.93	22.2	2.36
23.1	349	334	4.31	134	94	4.4	63	11.1	32.7	2.08	18.9	0.86
18.4	308	273	4.39	142	107	4.2	51	13	44.5	2.1	18.4	1.74
21.8	601	465	3.62	132	96	3.2	45	11.2	40	3.13	18.6	1.22
10.2	269	232	3.85	133	97	5.2	65	11.2	31.5	2.39	18.7	1.32
77.6	633	564	4.03	132	96	3.7	46	10.8	32	3.34	18.3	0.81
1166	811.8	1088	4.5	127	92	18.5	228.4	15.5	138.7	2.43	151.8	5
27	874	667	3.72	131	93	4.2	60	11.2	39.1	2.31	19.9	2.32
51.7	753	568	2.52	140	96	8.1	122	11.8	38.5	1.77	19.5	6.45
47.2	934	589	4.05	137	99	4	48	11.4	39.8	2.08	20.3	5.41
30.8	2326	1396	4.09	141	99	4	77	12.5	45.1	1.3	23.4	3.65
41.6	1323	989	4.43	140	105	8.5	110	12.1	52.4	2.2	22.2	7.05
24.4	848	649	3.84	133	94	4.4	63	12.2	45.3	1.95	19.2	3.4
102.2	3500	1716	4.71	141	99	5	66	13.8	40.7	1.31	22.8	8.82
57.1	2196	1592	3.5	139	103	4.3	62.2	12.4	46.8	1.78	19.4	5.88
8.5	253	216	3.3	141	97	2.5	67.5	11.9	30.8	2.09	20.2	1.92
18.1	360	288	4.75	142	100	5.8	51	13.1	39.7	2.24	18.8	5.59
24.3	185.5	157	4	131	96	6.9	88	13.4	34.2	1.87	17.5	0.53
15.1	237	200	4.85	139	97	4.7	69	15.5	39.1	2.46	19.1	6.06
19.6	315	259	4.71	134	98	4.6	71	14	41.9	2.24	19.5	1.42
11.4	245	221	4.76	140	101	3.9	66	13.8	48.4	2.32	19.4	4.07
145.2	3500	1077	5.14	146	97	26.5	342	14.8	140	1.56	25.4	2.72
14.9	285	240	3.74	133	97	5.6	70	12.4	31	2.46	19	1.15
132.9	1410	1081	4.88	132	94	15.6	161	14	50.4	2.12	21.6	17.84
176.4	1396	1078	4.05	126	88	8.7	109	13.4	57.6	1.6	22.2	62.5
25	802	484	4.05	142	100	5.3	76	11.7	40.7	1.76	19.8	0.75
40.3	459	285	4.37	132	91	4.1	74	11.1	43.6	2.24	19	0.78
14.5	279	218	3.98	133	93	6	61	13	41.7	2.36	17.7	1.37
15.7	347	219	3.84	138	102	2.7	48	12.1	32.6	1.96	19.4	2.01
17.5	373	263	4.01	134	94	7	65	13	44.6	2.21	18.6	3.42
85.7	628	473	3.79	134	99	10.7	93	13.5	57.8	2.08	20.9	4.53
19.1	304	243	3.63	133	97	5.8	60	13.5	39.7	2.27	18.3	4.29
10.9	508	369	3.76	128	89	4.8	70	12.9	41	2.16	19.1	1.89
22.6	497	377	3.3	136	96	9	103	12.6	58.4	1.72	18.6	6.7

22.1	724	490	3.1	132	93	0.1	72.2	13.4	50.8	1.45	20.9	7.77
35.4	705	464	4.1	138	104	4	59.7	11.6	49.7	1.88	21.2	1.92
11.2	422	313	3.18	131	93	3.4	54	12.1	46.4	2.13	19.9	0.89
44.3	744	537	3.66	134	95	5.2	89	11.7	66.1	1.99	19.7	8.13
20.3	321	224	3.51	127	90	6.5	73	13.1	44.6	1.78	17.3	6.27
16.8	726	482	3.97	134	94	3.3	70	12.8	44.7	1.5	19.9	18.47
31	673	423	3.79	132	93	5.2	78	12.9	42.8	2.19	19.2	8.67
31.4	1471	964	4.23	129	96	6.8	40	12.6	56.6	1.74	20.8	59.92
19.4	456	331	4.3	137	102	8.1	94	12.1	41.2	2.06	20	2.04
63.6	591	463	4.53	140	98	7.3	61	14.4	49.7	1.28	22.6	4.47
42.9	583	449	3.17	130	89	2.8	51	12.7	51.9	2.36	19.3	7.37
36.6	721	645	3.4	143	101	10	81.4	12.3	48.7	1.94	19.8	1.19
97.2	712.7	472	3.7	131	102	23.8	195	12.8	62.6	1.64	20.4	11.77
26.8	576	467	3.77	134	98	5.7	52	11.7	37.1	1.67	20.5	1.02
109.1	973	655	4.15	138	101	5	85	12.8	53.7	1.82	20.6	8.53
40.7	1171	833	5	132	98	5.2	50	11.1	40.5	2.6	19.3	0.79
19	359	267	3.42	134	96	4.6	70	12.7	36.4	2.56	17.4	3.07
46.4	1212	764	4.07	135	98	5.1	78	11.4	40.9	1.86	18.4	5.3
57.2	787	526	3.19	132	94	4.8	65	13.1	43.4	1.95	19.6	12.8
14.2	235	169	3.24	138	1.2	4.2	56	12.8	34.3	2.49	17.5	2.36
60.8	1039	694	3.98	135	99	12.7	131	12.3	45.3	2.3	18.1	5.12
4.7	335	272	3.38	131	92	5.3	68	12.1	37.5	2.17	16	1.07
36.1	1154	764	3.89	135	98	4.6	52	12.5	32.7	1.92	17.7	3.5
96.6	1748	1138	4.17	126	93	8.7	94	12.2	50.3	1.71	19.6	22.56
14.5	255	193	3.7	136	102	2.8	81.9	12.6	37.6	2.66	16.8	1.28
19.3	579	416	3.27	132	96	1.9	55	11.7	30.3	1.79	18.3	2.33
79.8	2071	53	3.97	133	94	5.3	80	13.9	48.3	1.07	23.1	41.04
41.9	953	931	3.5	137	101	9.5	125.9	12.7	46.9	2.32	18	1.53
36.3	510	485	3.66	131	93	5.4	92	12.4	42	1.99	17.3	8.31
16.6	286	204	3.49	138	100	4.6	65	12.3	38.4	2.03	16.6	3.77
418.1	4373	3846	6.8	144	100	18.4	207.4	17.8	146.6	0.98	29.4	20.44
57.1	1088	711	3.79	131	97	3.6	58	12.1	46.9	1.64	19.7	19.74
15.3	259	195	2.93	141	103	2.4	40	12.9	54.2	1.78	17	2.33
26.8	328	223	3.93	126	93	2.6	48	12.6	48	1.92	18.9	3.37
130	1739	1275	4.12	136	104	11	143	13.8	67.1	1.18	22.8	10.67
33.9	759	503	3.37	133	96	2.8	71	13.1	40.1	1.68	17.6	6.78
74.3	1695	1127	4.42	130	99	6.1	67	13.2	52	1.53	19.8	29.23
36.6	1475	1099	2.99	131	97	2.1	43	11.7	41.3	2.14	18.1	3.07
41	837.9	686	4	136	100	21	238.6	12	37.8	3.6	16.2	1.2
29.5	1041	748	3.36	126	91	2.3	45	11.7	39.1	2.24	17.9	1.34
110	3193	2179	5	131	100	14	98.5	14.6	120.8	1.17	25.2	2.98
76.6	1531	950	3.98	125	94	3.4	66	14.8	52.7	1.51	18.5	9.79
57.3	763	568	4.26	132	97	4.6	61	11	32.8	2.06	18.7	2.55
55.2	631	408	4.12	122	88	5	68	13.4	48.9	1.24	19.8	10.83
22.1	655	471	3.24	130	98	2	54	14.1	49.7	1.32	20.7	28.46
16.4	502	386	3.99	138	99	8.1	76	11.4	33	2.03	17.9	1.07
18.9	285	208	3.24	131	100	2.6	84	13.5	41.3	1.88	19.2	4.37
54.1	561	419	3.93	135	97	9	113	12.6	53	1.86	18.4	0.97
18.2	210.7	158.6	3.83	128.4	93.1	7.57	87.5	13.4	39.8	2.13	18.5	0.81
12.3	281	257	3.24	134	101	2.9	40	14.5	41.9	1.39	19.5	1.62
28.3	360	258	3.66	133	101	9	45	10.9	31.3	2.26	17.8	1.19

132.4	1205	907	4.39	128	93	6.6	57	13.3	66	1.28	21.9	15.6
53.4	1082	669	3.05	131	95	2.7	68	12.1	44.2	2.09	19.2	5.12
13.3	312	188	2.98	136	101	5	77	12.5	37	2	18.8	5.56
57.6	867	633	3.71	132	100	4.8	65	11.6	34	1.74	20.2	3.41
12.9	251	192	3.97	130	94	10.8	147	13.8	36.1	2.09	18.2	11.43
19.8	316	255	3.15	131	96	6	75	14.2	42.1	1.86	17.7	3.81
18.4	462	341	4.2	128	91	8.5	99	13.1	47.2	2.68	17.2	4.48
33.8	519	345	3.81	131	94	3.7	63	12.2	35.5	2	18.8	15.8
18.4	292	219	3.32	139	100	3.1	75	12.5	30.2	2.44	17.3	0.75
16.6	673	660	3.9	134	98	5	80	13.5	48.2	1.75	18.9	22.66
16.6	216	159	3.86	141	104	3.5	61	11.3	25.7	2.35	17.5	0.52
58.8	665	482	4.15	138	104	7.6	72	11.7	27.6	1.94	17.7	2.46
19.9	290	227	3.67	137	106	3.3	48	11.3	27.7	2.38	16.6	0.94
122.5	1173	938	3.29	119	89	1.6	33	13.2	66.6	1.21	22.9	7.69
34.4	757	549	3.42	128	91	4	74	11.6	40	2.11	18.8	3.2
59.4	782	578	3.8	128.6	95	9.33	114.7	12.7	39	1.87	19.4	3.91
25	610	449	3.4	133	94	5.3	84	12	40	2.26	17.9	1.44
89.2	1695	1214	3.53	134	100	12.1	136	12.5	45.4	1.55	20.9	14.99
23.9	543	404	3.59	140	103	4.4	52	11.3	33.6	2.13	18.1	1.34
12.6	273	189	5.27	141	99	38.7	878	13.4	55.2	1.8	23.7	0.71
33.6	738	493	3.69	140	107	6.9	84	12.7	50.3	1.56	20.2	8.57
42.1	615	381	3.58	138	99	8	83	13.2	41.3	1.53	20.4	6.91
129.9	4157	3119	3.88	143	102	6.5	50	14.8	89.8	1.66	23.2	9.52
25.8	318	233	4.07	131	94	12.4	82	11.1	31.1	2.32	18.7	4.61
29.2	777	469	3.37	140	140	4.2	63	12.9	41.8	1.98	18.7	21.43
13.9	318	222	4.36	135.2	96.9	5	57.8	11.6	41.4	2.14	18.1	1.8
27.7	1071	707	4.5	136.8	105.3	6.2	73.9	13.9	48.1	1.7	20.8	63.53
53.4	847.6	582	3.84	139.5	103.6	4.5	61.5	12.3	47.6	2.21	19	1.04
24.3	837.1	681	4.18	137	108.4	3.7	61	12.7	40.2	2.52	18.9	2.54
23.8	565.7	391	4.05	138.6	95.4	4	63.4	14.4	55.1	1.52	20.8	130.3
23.4	237.1	185	3.38	134.8	100.6	6	59.8	12.9	38	2.1	17	0.78
20.6	221	187	4.01	136.2	104.8	4.9	102.1	12.9	36.5	1.61	18	2.08
7.8	250.5	181	2.95	136.7	102.1	4.5	50.4	12.9	34.1	1.85	18.2	5.83
67.5	2162	1496	3.91	135.3	103.7	3.78	85.3	14.3	56.4	1.37	22.1	8.73
22.1	559.9	405	4	138.4	102.8	12.4	113.3	12.6	40	2.24	19	13.08
60.5	1507	692	4.22	137.2	104.6	6.2	86.8	12.5	41.7	1.62	20.8	8.02
21.1	272.8	192	3.98	136.7	105	5.9	65.6	12.2	45	2.19	17.1	1.98
19.1	450.1	307	3.82	137.2	102.8	7	61.9	12.7	41.8	1.85	19.2	1.8
25.6	550.7	366	4.78	140.8	105.6	9.1	71.1	11.7	51.3	1.74	19.1	5.09
12.1	245.5	209	3.54	132.3	99.3	6.4	68.5	13.6	37.2	1.82	17	2.25
19.6	470.8	344	4.08	138.6	101.8	10.5	122.2	13.3	37.3	2.34	19	3.58
13.7	257.3	198	4.08	129.3	96.7	8.9	117.2	12.9	31.8	2.34	17.6	3.16
18.3	249.6	209	3.1	140.5	102.7	5.1	52.2	13	30.5	2.48	17	2.69
29.5	556.7	480	3.62	140.5	106	4.3	74.4	12.7	37	1.95	18.6	3.55
19.8	634.4	556	3.21	136.3	101.6	5.9	80.8	11.4	40.8	1.8	19.4	4.37
32.5	801.7	586	3.97	128.5	94.2	4	42.4	14.4	46.8	1.82	18.7	14.4
43.3	883.1	713	3.63	122.9	91	7.64	100.7	11.4	30.6	2.37	18.1	1.52
26.3	619.2	456	3.89	136.8	95.7	5.4	42.1	11.5	38.7	2.23	19.3	2.14
33.4	721.5	458	3.88	131.1	96	7.9	74.7	12.6	38.9	1.52	20.9	7.17
20.6	465.7	387	3.98	134.4	100	3.1	67.4	12.1	32.6	2.34	17.2	3.37
20.5	433.9	295	2.89	137.5	94.9	6.6	71.2	12.1	31.2	1.65	20.5	2.03

61.5	1404	1030	3.61	139.1	105.3	2.8	79.2	11.3	35.5	2.42	18.8	3.81
12.4	379.2	262	3.15	130.6	94.8	4.2	59.7	13	32.8	2.26	16.9	4.37
52	672.4	599	3.63	128	95	4.42	79	12.3	37.8	2.01	18.8	1.41
33.7	949.6	689	4.77	130.5	98.3	12.64	149.7	13	42.9	1.59	20.8	4.43
30.9	556.6	345	3.85	134.6	102.2	14.85	192.1	12.4	36.9	2.06	20.6	1.85
13.3	200.6	151	4.57	136.9	107	22.2	138	11.9	35.9	1.78	20.6	0.76
55.3	1252	706	3.89	139.1	101.9	2.9	43.4	12.7	38.7	1.85	19.2	10.19
63.9	918.1	615	4.15	135.3	101.1	6.8	56.5	11.3	40.7	2.05	18.7	3.78
44	405.4	299	3.94	127.8	95.4	6	68	11.8	39.9	1.9	18.2	3.06
66.4	1021	732	4	132.5	102.2	6.1	78.9	12.8	35	2.58	18.3	6.46
34.9	612.4	472	3.45	137.2	102	9.6	88	13.9	42.1	2.61	17.5	2.38
39.4	652.2	443	3.97	134.4	98.4	7.8	91	11.4	33	1.94	20.5	1.15
14.9	242.6	195	2.75	133.8	97.9	7.2	107.1	12.6	33.7	2.4	16.7	1.19
23	228.5	170	3.72	136.4	101.8	5	53.2	11.6	25.2	2.24	17.8	1.83
66.6	900.6	581	4.78	122	90.4	10.61	146.5	12	37.5	2.29	19.9	3.01
48.2	677.9	462	3.51	138.1	102.8	3.3	65.4	12.9	54	1.77	20.4	3.83
36.6	1052	696	3.47	134.5	101.7	2.4	39.8	11.2	37.6	2.29	19.6	3.93
43.6	576.7	423	4.42	133.2	98.2	4.4	83.5	11.4	43.1	1.81	19.6	1.8
24	317.3	396	3.21	134.7	95.9	4.1	65.1	12.7	35.7	2.24	18.1	2.05
41.7	315.6	243	3.89	130.8	94.2	6.2	46.5	12.9	35.2	2.1	18.1	2.36
30.5	288.6	191	4.28	128.8	97.3	4.1	64.7	12	40.5	1.99	18.6	3.31
28.2	332.4	236	3.42	136.1	98.7	4.2	59.4	11.8	34.4	2.45	17	1.13
38.7	1383	1070	3.26	145.8	102.3	5.4	80.2	103	43.1	1.66	21.5	9.17
67.8	774.3	586	4.07	136.9	105.7	6.6	70.7	75	62.5	1.68	20.8	25.82
17.1	716.4	529	3.17	155.1	115.7	12.4	156.1	100	51.8	1.94	21.4	0.69
55	3121	2272	4.01	134	102.6	6.8	78.3	84	53.8	1.81	23.2	7.54
23.8	474.7	355	4.3	136.1	106.5	7.61	72.2	118	30	2.48	18.9	3.62
50.7	876	696	4.33	129.6	92.1	5.2	79.1	16.3	71.4	1.42	19.9	63.33
37.9	636.4	417	5.44	138.4	103.5	21.97	150.3	124	34.5	2.99	18.5	0.95
18.9	394.9	249	3.98	143.9	110.9	9.4	80.4	98	36.3	2.96	17.7	2.54
42.9	675.8	524	2.98	135.6	105	2.15	77	77	45	1.63	20.7	9.97
233.3	5714	4166	5.75	140.8	104	25.8	261.2	67	113.3	0.93	36.2	9.58
64	605.1	474	2.91	137.5	94	2.8	57.1	93	48.7	2.34	18.2	0.8
50.6	1547	1289	3.55	136.7	97	3.9	61.7	90	38.7	1.73	21.7	2.18
94.4	1455	1105	3.64	138.1	105.2	12.94	154	83	81.6	1.77	23.5	5.98
71.4	879	587	3.21	123.4	86.1	5.15	54.7	81	45.5	1.51	18.4	18.74
27	482	247	2.75	132.7	100.5	3.88	63.1	82	49.8	1.95	19	36.12
26.5	523.8	385	2.93	134.6	100.2	2.2	39.6	90	45.3	1.69	18.2	5.7
21.5	403.6	321	3.81	130.9	94.2	3	63.7	82	31.6	1.87	17.5	4.25
7.8	248.5	192	2.95	128.4	95.9	3.11	79.1	71	37.3	1.52	18	13.13
40.6	805.7	567	4.4	134.7	109.3	2.8	54.2	90	42.3	2.37	19.6	1.43
22.7	244.7	184	3.34	132	95.8	8.1	76.3	77	53.3	2.21	18.5	5.46
32.2	904.7	555	3.89	125.7	94	5.37	88.4	87	39.6	1.49	22.5	4.78
18	636.4	248	3.58	139.2	105.5	4.6	60.5	80	49.8	1.43	21.4	20.63
15.2	412.9	305	3.44	135.6		9.12	122.8	103	37.3	1.29	22.5	5.61
25.2	377.6	303	3.75	128.5	96.7	2	44.8	98	42.6	1.46	20.9	1.05
49.7	775.3	429	3.03	137.6	99.8	9.8	142.9	77	53.3	1.34	21.8	20.69
19.2	966	772	3.97	138.5	105	2.1	51.8	107	33.3	2.87	17	0.64
67.8	885.3	464	4.31	138.6	107.6	7.27	96.1	84	48.6	1.57	23.2	18.93
47.8	907.5	563	4.14	137	107.5	6.39	104.2	63	113.9	1.19	25.7	33.97
31.3	568.8	321	3.8	133.9	94.7	3.5	55	74	42.3	1.97	18.5	4.71

81.4	3756	2335	3.74	128.2	90.4	35	49.1	101	53	1.7	20.1	8.77
20.5	258.4	195	3.34	143.1	100.6	4	66.7	87	47.8	1.11	28.8	2.18
21.4	790.2	550	3.06	136.6	93.8	4.5	94.8	72	44.2	1.47	21.3	3.22
34.4	1489	1207	4.52	137.6	100.6	2.2	48.2	82	54.1	1.59	21.2	3.26
21.7	242.4	165	4.29	141.6	108.2	4.28	96.7	53	59.5	1.69	20.5	18.84
123	1774	1151	4.26	134.1	103.5	10.4	101.4	69	64.5	1.8	21.5	9.57
88.8	1233	724	4.01	131.3	101.9	6.98	144.5	87	43.9	2.16	19.7	4.22
32	631.3	805	2.96	137	104.7	5.72	64.7	93	39.6	1.68	21	3.75
54.7	207.5	156	3.97	131.4	99.9	6.17	45.6	106	30.6	3.82	16.5	0.97
37.5	832.4	514	4.23	139	102.6	2.2	72.4	100	34.6	2.87	17.8	1.48
11.9	192	155	3.55	128.9	99.1	3.84	65.3	106	35.3	2.23	18.3	1.3
14.3	370.8	245	2.96	125	94.8	5.38	74.9	81	43.1	2.03	18.5	6.68
21.3	208	167	3.34	130.2	103.3	4.62	56	68	49.5	2.1	19.8	7.83
18.2	270.9	156	2.8	131.7	96.5	3.2	60.4	93	45.5	1.92	19.8	3.4
11	2113	159	3.65	133.3	104.2	2.8	54.7	84	39.8	2.14	18.4	6.43
22.3	369.7	259	3.59	136.3	103.8	6.8	116.3	93	41.6	2.01	19.6	2.95
30.6	1326	845	3.29	134.7	99.6	8.13	94.1	104	34.9	1.85	20	2.5
12.1	320.1	233	3.37	131.6	97.3	5.89	67.7	87	38.8	1.23	17.8	3.7
39.5	2041	1263	3.9	127.2	92.6	4.03	59.1	95	37	2.07	19.9	26.49
14	148.2	105	3.32	129.2	101	4.61	71.8	93	44.2	1.61	18	2.04
18.2	536.8	321	3.72	138.6	105.1	6.6	74.3	100	42.1	2.24	19.4	2.77
16.8	538.2	403	3.12	122	90.4	5.32	65.4	90	36.9	2.01	19.4	5.88
17.4	405.6	375	3.56	127.7	100.8	3	28.7	104	38.1	2.45	18	2.04
27.5	347.1	189	3.45	139.4	110.6	3.2	50.7	100	37.6	2.19	19.1	1.1
56.2	1327	1011	3.69	137.5	3.88	11.61	145.2	86	57.5	1.11	23.9	2.44
42.9	846.7	529	3.33	130.3	98.5	4.6	50.3	94	51.6	2.14	19.3	11.68
39.7	1964	1023	3.31	131.9	103.2	6.1	87	83	49.6	1.53	18.2	10.82
60.6	1024	872	4.32	134.4	105.7	5.8	71.1	103	39.1	1.9	20.1	4.63
35.9	1568	1322	3.69	128.3	97.7	4.34	63.7	79	69.2	1.46	18.2	11.7
16.9	340.8	220	3.53	132	101.7	4.06	62	93	45.4	2.08	19.7	5.29
16.2	323.8	289	4.33	129.9	99.1	9.8	91.6	93	36.2	1.74	17.4	1.16
7.9	216.3	154	3.73	133.3	96.6	8.02	99.3	73	43.1	2.03	20.3	4.01
41.2	1420	1128	3.87	138.7	102.6	5.4	66.5	82	56.4	1.41	24.2	3.71
16.1	356	299	3.65	130.4	93.4	14.07	164.3	84	42.3	2.84	19.7	1.15
3.6	202.2	163	3.24	133	93.6	4.95	126.7	79	34.4	1.94	16.6	32.64
48.7	799.8	512	3.47	131.9	97.6	7.42	120.4	95	41.4	2.95	17.2	2.34
9.6	206.7	177	3.54	139.4	103.6	5.56	62.7	87	42.7	2.21	18.2	1.98
98.9	537.2	402	4.66	136.1	98.5	7.2	83.3	71	30.4	1.78	18.6	24.49

r(ug/L)
